# Transcriptome, microRNA, and degradome analyses of the gene expression of Paulownia with phytoplamsa

**DOI:** 10.1186/s12864-015-2074-3

**Published:** 2015-11-04

**Authors:** Guoqiang Fan, Xibing Cao, Suyan Niu, Minjie Deng, Zhenli Zhao, Yanpeng Dong

**Affiliations:** Institute of Paulownia, Henan Agricultural University, Zhengzhou, Henan 450002 P. R. China; College of Forestry, Henan Agricultural University, Zhengzhou, Henan 450002 P. R. China

**Keywords:** Paulownia witches’ broom, Transcriptome, microRNAs, Degradome, Gene expression

## Abstract

**Background:**

Paulownia witches’ broom (PaWB) is a fatal disease of Paulownia caused by a phytoplasma. In previous studies, we found that plants with PaWB symptoms would revert to a healthy morphology after methyl methane sulfonate (MMS) treatment. To completely understand the gene expression profiles of the Paulownia-phytoplasma interaction, three high-throughput sequencing technologies were used to investigate changes of gene expression and microRNAs (miRNAs) in healthy *Paulownia tomentosa* plantlets, PaWB-infected plantlets, and PaWB-infected plantlets treated with 60 mg · L^−1^ MMS.

**Methods:**

Transcriptome, miRNAs and degradome sequencing were performed to explore the global gene expression profiles in the process of Paulownia tomentosa with phytoplasma infection.

**Results:**

A total of 98,714 all-unigenes, 62 conserved miRNAs, and 35 novel miRNAs were obtained, among which 902 differentially expressed genes (DEGs) and 24 miRNAs were found to be associated with PaWB disease. Subsequently, the target genes of these miRNAs were predicted by degradome sequencing. Interestingly, we found that 19 target genes of these differentially expressed miRNAs were among the 902 DEGs. The targets of pau-miR156g, pau-miR403, and pau-miR166c were significantly up-regulated in the *P. tomentosa* plantlets infected with phytoplasma. Interaction of miRNA -target genes mediated gene expression related to PaWB were identified.

**Conclusions:**

The results elucidated the possible roles of the regulation of genes and miRNAs in the Paulownia-phytoplasma interaction, which will enrich our understanding of the mechanisms of PaWB disease in this plant.

**Electronic supplementary material:**

The online version of this article (doi:10.1186/s12864-015-2074-3) contains supplementary material, which is available to authorized users.

## Background

Paulownia is a fast-growing tree with lightweight, soft, and straight-grained wood. It is native to China where it is used for house construction, solid biofuel, furniture, cellulose pulp, and Chinese herbal medicine [1–3]. Additionally, Paulownia can be used as an environmental protection tree because of its deep root system and ability to grow on nutrient-poor soil [1, 4].

Phytoplasmas are plant pathogenic bacteria, previously called mycoplasma-like organisms, which primarily infect plant phloem sieve cells. Phytoplasmas have different classifications and each classification has evolved diverse strains [5]. In nature, phytoplasmas are transmitted by leafhoppers and planthoppers. Hundreds of plants can be infected, and phytoplasmas cause a wide range of disease symptoms, including witches’ broom, stunting, yellowing of leaves, and proliferation of axillary buds, which can result in serious losses in agriculture, forest, and horticulture [6–9].

Paulownia witches’ broom (PaWB) is a disease caused by a phytoplasma. The PaWB phytoplasma was first found in 1967 [10], and since then numerous researchers have investigated the mechanisms of PaWB disease, while other researchers have examined the PaWB pathogen including the mechanism of infection [11, 12], diagnosis [13–15], methods of preservation [16], seasonal variation [14, 17], and characteristic features of phytoplasma plasmids, the Sec protein, and virulence factor [18–21]. Studies on the PaWB host have referred to an array of physiological and biochemical variations [22, 23], disease control [24], and breeding of disease resistant varieties [25]. Recently, our research team found that phytoplasma-infected Paulownia had decreased DNA methylation levels, and showed that phytoplasma-infected plantlets treated with 60 mg · L^−1^ methyl methane sulfonate (MMS) returned to normal morphology in which the phytoplasma could not be detected by nested PCR [26–28]. In these studies, some genes were differentially expressed at primary and secondary metabolism, photosynthesis, cell cycle and division, cell wall biosynthesis and degradation, hormone biosynthesis, plant-pathogen interaction, circadian rhythm, phenylpropanoid metabolism, folate synthesis, and fatty acid synthesis were obtained by transcriptome analyses, at the same times, some miRNA related to hormone signaling were also obtained [29–34]. The expression levels of these genes were determined only at the transcriptional level, and changes at the post-transcriptional level have not yet been reported.

MicroRNAs (miRNAs) are non-coding RNA molecules (21–23 nucleotides) that mediate gene expressions by RNA silencing at the post-transcriptional level [35]. Mature miRNAs are processed from primary transcript (pri-miRNA) [36] and incorporated into an RNA-induced silencing complex (RISC) that can bind directly to complementary target mRNA transcripts, which are then cleaved or the RISC can reversibly inhibit the translation process [37]. Until now, increasing evidence has indicated that miRNA-mediated gene expression may play important roles in the responses of plants to abiotic and biotic stresses [38, 39]. For example, phytoplasma-responsive miRNAs that mediated gene expression levels have been reported in Mexican lime and mulberry [40, 41]. In Paulownia under abiotic stress, miRNA-mediated gene expression changes have been reported [42–44]; however, miRNAs involved in the response of Paulownia to phytoplasma infection have not yet been reported. In the present study, we explored the global gene expression profiles in *Paulownia tomentosa* at both the transcriptional and post-transcriptional levels using a combination of transcriptome, miRNAs and degradome sequencing. This knowledge will help in understanding the mechanisms of PaWB disease.

## Methods

### Plant material, treatments, and RNA isolation

Tissue culture plantlets of healthy plantlets (HP) and PaWB-infected plantlets (PIP) of *P. tomentosa* were obtained from the Institute of Paulownia, Henan Agricultural University, Zhengzhou, Henan Province, China. The plantlets were cultured for 30 days on 1/2 MS medium [45] before being clipped. After that, the terminal buds of 1.5 cm PIPs were transferred into 100-mL triangular flasks containing 1/2 MS culture medium containing 0 (PIP) or 60 mg · L^−1^ MMS (PIP-60). The terminal buds of 1.5 cm HP were transferred into the same medium without MMS as the control. All the plantlets were first cultured at 20 °C in the darkroom for 5 days, and then cultured at 25 ± 2 °C under 130 μmol · m^−2^ s^−1^ intensity light with a 16/8 h light/dark photoperiod. Twenty-five days after beginning the cultivation in the tissue culture room, the terminal buds of 1.5 cm plantlets in each treatment group were sheared, immediately frozen in liquid nitrogen, and stored at −80 °C. For each treatment an average of 60 terminal buds were planted in 20 flasks. Each treatment was performed in triplicate. Total RNAs were extracted from the terminal buds using TRIzol reagent (Invitrogen, Carlsbad, CA), according to the manufacturer’s instruction, and then treated with DNase I (RNase-free). The quality of the RNAs was assessed using a NanoDrop 2000 (Thermo Scientific, Wilmington, DE).

### Transcriptome sequencing and *de novo* assembly

Oligo (dT) magnetic beads were used to isolate the mRNA, which was mixed with fragmentation buffer to obtain short fragments. First-strand cDNA was synthesized using the short fragments as templates. Second-strand cDNA was synthesized using RNase H and DNA polymerase I. The short cDNA fragments were purified with the QIAquick PCR (Qiagen) extraction kit, and then were dissolved in EB buffer for end reparation and addition of a single nucleotide A to the 3′end to prevent them from ligating to one another during the adapter ligation reaction. A corresponding single ‘T’ nucleotide on the 3′ end of the adapter provides a complementary overhang for ligating the adapter to the fragment. The multiple indexing adapters were ligated to the ends of the dscDNA, then the suitable fragments that had adapter molecules on both ends were used as the templates for PCR amplification, the cDNA libraries were qualified using a 2100 Bioanalyzer (Agilent Technologies, Inc., Santa Clara, CA) and the quality was assessed using an ABI StepOnePlus Real-Time PCR System (ABI, New York, NY, USA). After that, the library was sequenced on an Illumina/Solexa GAIIx platform (Illumina, San Diego, CA).

Data preprocessing was carried out to obtain high quality clean reads. The raw reads were first passed through Illumina’s built-in Failed-Chastity Filter software and reads that failed were removed. Clean reads were obtained after filtering out reads with adaptors, reads with more than 5 % unknown nucleotides, and low quality reads in which more than 20 % of the bases had a quality value of ≤10. The reads from the HP, PIP, and PIP-60 transcriptome libraries were then *de novo* assembled using the Trinity software (release-20121005) to generate all-unigenes [46]. The data used in this publication have been deposited in the NIH Short Read Archive database (http://www.ncbi.nlm.nih.gov/sra) and the study accession number are SRP057771 and SRP058902.

For annotation, the all-unigene sequences were used as queries in BLASTX searches (E-value <1.0E-5) to against the NCBI non-redundant (NR) protein database (http://www.ncbi.nlm.nih.gov) [47], Swiss-Prot protein database (http://www.uniprot.org/), Cluster of Orthologous Groups (COG) database (http://www.ncbi.nlm.nih.gov/COG), and the Kyoto Encyclopedia of Genes and Genomes (KEGG) pathway database (http://www.genome.jp/kegg). All-unigene sequences that did not match any of the sequences in any of these databases were annotated using ESTScan [48]. Gene Ontology (GO) functional annotations were assigned to the all-unigenes based on the GO annotations of the sequences that shared high similarity (≥70 %) in the BLASTX searches. The COG database was searched to analyze the all-unigene functional classification, and the KEGG database was searched to determine the unigene pathway annotations.

In order to identify differentially expressed genes between PIP vs. HP and PIP-60 vs. PIP, the fragments per kilo base of transcript per million mapped reads (FPKM) method [49] was used to calculate the expression levels as:

$$ FPKM=\frac{10^6C}{NL/{10}^3} $$

Where FPKM is the expression of unigene, C is the number of fragments that aligned uniquely to unigene, N is the total number of fragments that aligned uniquely to all-unigene, and L is the number of bases in the coding sequence of unigene.

The differentially expressed unigenes (DEGs) between any two libraries (PIP vs. HP and PIP-60 vs. PIP) were calculated according to the method by Audic [50], a hypergeometric test of GO and KEGG pathway enrichment was performed. The *p*-value was calculated as follows:

$$ p=1-{\displaystyle \sum_{i=0}^{m-1}\frac{\left({}_i^M\right)\left({}_{n-i}^{N-M}\right)}{\left({}_n^N\right)}} $$

Where *N* represents the total number of genes with GO annotation; *n* represents the number of DEGs in *N*; *M* represents the total number of all genes in each GO term; and *m* represents the number of DEGs in *M*. After applying the Bonferroni correction to the calculated p-value, we selected a corrected p-value of ≤0.05 as the threshold to determine significantly enriched GO terms for the DEGs. The p-value threshold in multiple hypothesis testing and analyses was determined by manipulating the false discovery rate (FDR) value [51], and two points (|log_2_ ratio| ≥ 1 and FDR ≤ 0.001) were used to judge the significance of the DEGs, then, KEGG pathway enrichment analysis was used to retrieve significantly enriched pathways among the DEGs against the whole transcriptome background. A Q-value was defined as the FDR analog of the p-value. After multiple testing and corrections, a Q-value of ≤ 0.05 was taken to indicate a significantly enriched pathway among the DEGs.

### Small RNA sequencing, identification of miRNAs and their target genes

Three small RNA (sRNA) libraries were constructed using the TruSeq Small RNA Sample Prep Kit (Illumina, San Diego, CA), according to the instruction of this kit, most mature miRNAs have a 5′‐phosphate and a 3′ ‐hydroxyl group, then the adapters are directly, and specifically, ligated to 5′ and 3′ end of the RNA molecule and an reverse transcription reaction is used to create single stranded cDNA, which was amplified by PCR and purified by polyacrylamide gel electrophoresis (PAGE). After that, the band containing the 22 nt RNA fragment with both adapters are a total of 147 nt in length, the band containing the 30 nt RNA fragment with both adapters are 157 nt in length, after removing the end adapters, the length of small RNA was 22-30 nt, therefore, the 140–160 bp gel fragments were cut out to produce the library for cluster generation and sequenced on the GAIIx platform following the manufacturer’s standard cBot and sequencing protocols. Then the small RNA sequences were processed using Illumina’s Genome Analyzer Pipeline software to filter out the low quality reads, adaptors, and 5′ primer contaminants, then analyzed for their length distribution and mapped onto the Paulownia unigene database using miRDeep2 [52], Perfectly matched sequences were analyzed using Blastall (http://www.ncbi.nlm.nih.gov/staff/tao/URLAPI /blastall/) against the GenBank (http://www.ncbi.nlm.nih.gov/) and the non-coding RNA (ncRNA) database (Release 10; http://rfam.sanger.ac.uk/) to remove ncRNAs (including tRNA, rRNA, snoRNA, and other ncRNA). The remaining sequences were searched against the plant mature miRNA Sanger miRBase database (Release 21.0; http://www.mirbase.org/) to identify known miRNAs. sRNAs that aligned to the mature miRNA sequences with no more than two mismatches were considered as potential conserved miRNAs. The potential novel miRNAs were identified using MIREAP (http://sourceforge.net/projects/mireap/) and RNAfold (http://rna.tbi.univie.ac.at/cgi-bin/RNAfold.cgi) [53] to fold flanking sequences and predict secondary structures. If the sRNA had a perfect stem-loop structure and followed the other criteria described [54], it was considered to be a candidate novel miRNA. The expression of miRNA in PIP and HP (PIP-60 and PIP) were normalized to obtained the expression of transcript per million. Based on the normalized expression analysis, the miRNA fold-change ≥ 1.0 or the fold-change ≤ −1.0, 0.01 < *P*-value <0.05 were used to judge the significance of the differentially expressed miRNAs between PIP vs. HP and PIP-60 vs. PIP. The formula [50] was as follows:

$$ Normalized \exp ression=\left( actual\kern0.5em  miRNA\kern0.5em  count/ total\kern0.5em  count\kern0.5em  of\kern0.5em  clean\kern0.5em  reads\right)\times \kern0.5em 1,000,000 $$

$$ Fold\kern0.5em  change\kern0.5em =\kern0.5em  \log 2\kern0.5em \left( normalized\kern0.5em  read\kern0.5em  counts\kern0.5em  in\kern0.5em  treatment\kern0.5em  library/ normalized\kern0.5em  read\kern0.5em  counts\kern0.5em  in\kern0.5em  the\kern0.5em  control\kern0.5em  library.\right) $$

*P*-values:

$$ \begin{array}{l}\\ {}\begin{array}{l}P\left(x\left|y\right.\right)=\left(\frac{{}_{{\displaystyle {N}_2}}}{{\displaystyle {N}_1}}\right)\kern0.5em \frac{\left(x+y\right)!}{x!y!{\left(1+\frac{{\displaystyle {N}_2}}{{\displaystyle {N}_1}}\right)}^{\left(x+y+1\right)}}\hfill \\ {}C\left(y\le y \min \left|x\right.\right)={\displaystyle \sum_{y=0}^{y\le y \min}\mathrm{p}\left(y\left|x\right.\right)}\hfill \\ {}D\left(y\ge y \max \left|x\right.\right)={\displaystyle \sum_{y\ge y \max}^{\infty}\mathrm{p}\left(y\left|x\right.\right)}\hfill \end{array}\end{array} $$

Where N1and N2 represent the total number of clean tags in PIP and HP (or PIP-60 and PIP), respectively, x and y represent the number of miRNAs surveyed in PIP and HP (or PIP-60 and PIP), respectively, C and D can be regarded as the probability discrete distribution of the *P*-value inspection.

To predict the potential target genes of the miRNA, three degradome libraries were constructed according to the methods of Addo-Quaye and German [55, 56]. In brief, poly (A) RNA was isolated from the total RNA of each sample using an Oligotex mRNA mini kit (Qiagen, Shanghai, China). A 5′RNA adapter containing a *Mme*I recognition site was added to the poly (A) RNAs that possessed a 5′-phosphate using T4 RNA ligase. Reverse transcription using oligod (T) and PCR enrichment were performed, and the PCR products were purified and digested with *Mme*I (New England Biolabs (NEB), Ipswich, MA, USA). A double-stranded DNA adapter was then ligated to the digested products using T4 DNA ligase. The ligated products were amplified and the resulting product was sequenced using an Illumina HiSeq™ 2000 system, then the data process using PAIRFINDER (version 2.0) Hao et al. [57]. The small RNA and degradome sequencing data used in this publication have been deposited in the NIH Short Read Archive database (http://http://www.ncbi.nlm.nih.gov/sra) and the study accession number are SRP060300 and SRP060876.

### Quantitative Real Time PCR (qRT-PCR) analysis

The expression levels of potential target genes and miRNAs related to PaWB in HP, PIP, and PIP-60 were validated by qRT-PCR. The primers for the genes were designed with Beacon Designer 6.0 software (Premier Biosoft International, Palo Alto, CA), and the stem-loop primers and the forward primers were designed based on the mature miRNA sequences, the reverse primer was the universal reverse primer. First-strand cDNAs of the three samples were synthesized using a iScriptcDNA synthesis kit (Bio-Rad, Hercules, CA), and then amplified on a Bio-Rad CFX96TM Real-Time System (Bio-Rad, Hercules, CA). The PCR parameters were 95 °C for 1 min, then 40 cycles at 95 °C for 10 s and 55 °C for 15 s. 18S rRNA and U6 served as the internal reference gene for the target genes and miRNAs, respectively. The results were analyzed using the 2^-ΔΔCt^ method. Each gene was analyzed in three replicates. The statistical analyses were performed using SPSS 19.0 software (IBM Corp., Armonk, NY). The primers for qRT-PCR as shown in Additional file 1: Table S1, Additional file 2: Table S2 and Additional file 3: Table S3.

## Results

### Transcriptome sequencing and *de novo* assembly

To obtain an overview of the transcriptional information of PaWB-infected Paulownia, three cDNA libraries (HP, PIP, and PIP-60) were constructed and sequenced on an Illumina/Solexa GAIIx platform. After filtering 48,006,640 (HP), 75,244,384 (PIP), and 58,962,324 (PIP-60) clean reads were obtained with N percentages of 0.08, 0.11, and 0.03 %, respectively, and GC contents of 46.59 % (HP), 46.55 % (PIP) and 46.84 % (PIP-60), respectively (See Additional file 4: Table S4). The clean reads were assembled and a total of 98,714 all-unigenes with a mean length of 1133 bp were obtained; the N50 was 1875 bp. Among these all-unigenes, 59,973 (60.75 %) were longer than 500 bp and 41,615 (42.16 %) were longer than 1000 bp (See Additional file 21: Figure S1), demonstrating the high quality of the assembly in the three libraries. The correlation coefficient of the expression of HP was shown in Additional file 22: Figure S2. The results of duplicates showed linear correlations with the corresponding one, the pearson r values was 0.811.

To identify the functions of the all-unigenes, we used them as queries in BLASTX searches (E-value <1.0E-5) against the NR, NT, Swiss-Prot, KEGG, COG, and GO databases, and identified 65,928 all-unigenes (66.79 % of the 98,714 all-unigene) that aligned to known sequences in one or more of these databases (Table 1). The E-value distribution of the top matches against NR sequences showed that 20 % of the mapped sequences had strong similarity (0 < E-value < 1e^−100^), 20 % had some similarity (E-value = zero) (See Additional file 5: Table S5). The similarity distributions are shown in Additional file 6: Table S6 and the similarity species distribution is showed in Additional file 7: Table S7.

**Table 1:** Annotation of all-unigenes of *P. tomentosa*

Database	Number of annotated unigenes	Percentage of annotated unigenes (%)
Nr^a^	63,686	64.52
Nt^b^	57,627	58.38
Swiss-Prot^c^	41,053	41.59
KEGG^d^	38,181	38.68
COG^e^	26,245	26.59
GO^f^	52,571	53.26
All	62,928	63.75

^a^Non-redundant protein sequence database

^b^Nucleotide sequence database

^c^Swiss-Prot Protein Sequence database

^d^Kyoto Encyclopedia of Genes and Genomes database

^e^Clusters of Orthologous Groups database

^f^Gene Ontology database

**Fig. 1:**
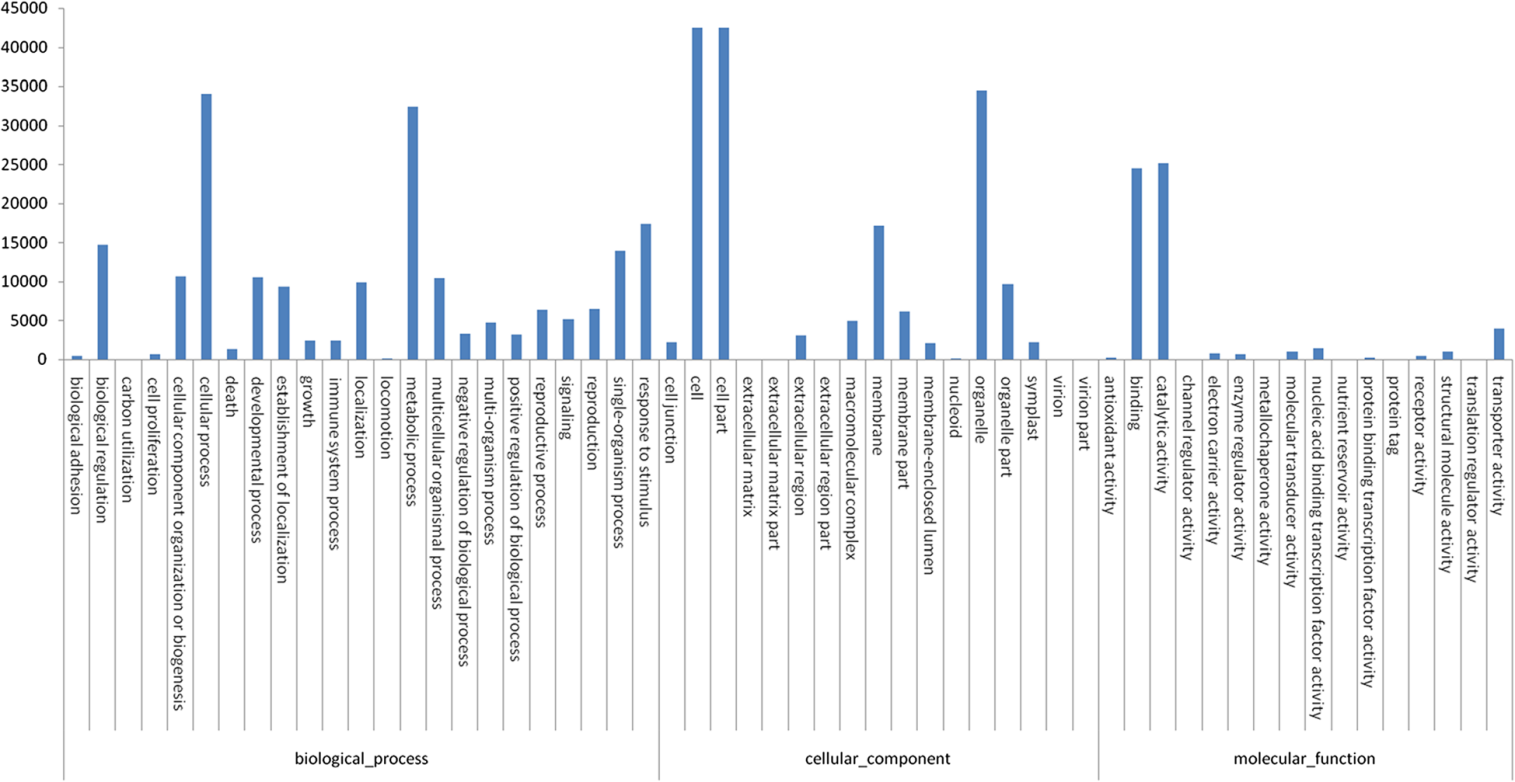
GO classification of all-unigene of *P. tomentosa*

GO functional annotation was used to assign possible functions to the 65,928 mapped all-unigenes, and 52,571 (53.26 % of the 98,714 all-unigenes) were allocated terms in 56 subgroups under the three main GO categories (biological process, cellular component, and molecular function) (Fig. 1). The results of the GO functional enrichment analysis are shown in Additional file 8: Table S8. COG functional annotation also was used to predict the functions of the all-unigene, and 51,187 (51.85 % of the 98,714 all-unigenes) were assigned to 25 COG categories (Additional file 23: Figure S3), among which “general functions prediction only” (9174; 17.92 %) was the most abundant category, while “cell motility” (341; 0.67 %) was the smallest category (See Additional file 9: Table S9). In the KEGG pathway analysis, 38,181 all-unigene (40.3 % of the 98,714 all-unigenes) were mapped to 128 KEGG pathways (See Additional file 10: Table S10), among which “metabolic pathways” (8420; 22.05 %) was the most abundant, while “nuclear structure” (2; 0 %) was the least abundant.

### Small RNA sequencing, identification of miRNAs and their target genes

The sRNA sequencing of the HP, PIP, and PIP-60 libraries generated a total of 18,927,307 (HP), 12,577,258 (PIP);15,400,049 (PIP-60) sRNA reads (Table 2), in these three libraries, the majority of the sRNAs were 20–24 nt in size, the detailed data for length distribution were shown in Additional file 11: Table S11, Additional file 12: Table S13 and Additional file 13: Table S13, among which 62 (HP), 55 (PIP), and 48 (PIP-60) conserved miRNAs belonging to 20 miRNA families were identified (See Additional file 14: Table S14). Among these families, members of the pau-miR166 family were the most abundant, representing an average of 75.29 % of the reads generated from the three libraries, while members of the pau-miR171 and pau-miR167 families were the least abundant and they were found only in the HP library (See Additional file 14: Table S14). In addition to these conserved miRNAs, 35 (HP), 32 (PIP), and 32 (PIP-60) potential novel miRNAs were detected; their sequence information and hairpin structures are shown in Additional file 15: Table S15, Additional file 24: Figure S4. The expressions of the conserved and novel miRNAs in PIP vs. HP and PIP-60 vs. PIP were compared (*P*-values < 0.05 and |log_2_ Ratio| ≥ 1), and 26 miRNAs with significantly different expressions were obtained. Five of these miRNAs might be involved in PaWB disease.

**Table 2:** sRNA category and statistics of *P. tomentosa*

Sample	Category	Total	Mapping to genome	miRNA	rRNA	snRNA	snoRNA	tRNA	Unann^a^
HP	Unique sRNAs	3,007,699	533,911	1436	139,255	3828	2317	19,995	2,840,868
	Percent (%)	100 %	17.75 %	0.05 %	4.63 %	0.13 %	0.08 %	0.66 %	94.45 %
	Total sRNAs	18,927,307	13,291,576	1,062,535	6,491,609	21,479	37,888	475,698	10,838,098
	Percent (%)	100 %	70.22 %	5.61 %	34.30 %	0.11 %	0.20 %	2.51 %	57.26 %
PIP	Unique sRNAs	3,784,746	396,525	1549	94,142	2307	1487	15,590	3,669,671
	Percent (%)	100 %	10.48 %	0.04 %	2.49 %	0.06 %	0.04 %	0.41 %	96.96 %
	Total sRNAs	12,577,258	5,983,607	1,458,656	2,177,538	8654	16,400	209,833	8,706,177
	Percent (%)	100 %	47.57 %	11.60 %	17.31 %	0.07 %	0.13 %	1.67 %	69.22 %
PIP-60	Unique sRNAs	4,186,767	453,555	1488	97,452	2941	2089	13,703	4,069,094
	Percent (%)	100 %	10.83 %	0.04 %	2.33 %	0.07 %	0.05 %	0.33 %	97.19 %
	Total sRNAs	15,400,049	7,744,503	1,310,896	3,179,539	11,170	30,220	239,874	10,628,350
	Percent (%)	100 %	50.29 %	8.51 %	20.65 %	0.07 %	0.20 %	1.56 %	69.02 %

^a^Unnanotied

To understand the functions of the PaWB-responsive miRNAs, degradome sequencing was carried out to identify the targets of the miRNAs, according to the rules used for target prediction described by Allen et al. [58] and Schwab et al. [59], a total of 70 unique target genes were cleaved by 12 conserved miRNA families and five novel miRNAs. The different sites of these 70 targets cleaved were shown in Additional file 16: Table S16.

### Identification of genes and miRNAs related to PaWB

To explore the DEGs related to PaWB, we compared changes in the gene expression levels in the PIP vs. HP and PIP-60 vs. PIP libraries, and found 902 genes that were differentially expressed in the two comparisons (267 DEGs up-regulated in HP vs. PIP and down-regulated in PIP vs. PIP-60, and 635 DEGs down-regulated in HP vs. PIP and up-regulated in PIP vs. PIP-60) (Additional file 25: Figure S5 and Additional file 17: Table S17), and these DEGs were enriched in GO and KEGG database. According to the GO analysis, cell (401, 44.46 %), catalytic activity (285, 31.60 %) and cellular process (303, 33.60 %) were the top three GO term that involved in the most DEGs, conversely, nuclear chromosome part (4, 0.44 %), cation channel activity (3, 0.33 %) and arginine transport (2, 0.22 %) were the least GO term (See Additional file 18: Table S18); Based on the KEGG analysis, the above DEGs mapped to 92 KEGG pathways, metabolic pathways (132, 14.63 %), biosynthesis of secondary metabolites (90, 9.98 %) and plant-pathogen interaction (34, 3.77 %) were the top three pathway that involved in the most DEGs, while pentose phosphate pathway (3, 0.33 %), fatty acid elongation (2, 0.22 %) and anthocyanin biosynthesis (1, 0.11 %) were the least pathway (See Additional file 19: Table S19). We also compared the gene expression levels of miRNAs in the PIP vs. HP and PIP-60 vs. PIP libraries and found 13 conserved miRNAs and three novel miRNAs that were up-regulated and 13 conserved and one novel miRNAs that were down-regulated in HP vs. PIP. We also found differences in the PIP vs. PIP-60 comparison; seven conserved and one novel miRNAs were up-regulated and three conserved and one novel miRNAs down-regulated in PIP-60 vs. PIP. Among the differentially expressed miRNAs, most of the conserved miRNAs were predicted to target several genes; for example, the miR156 and miR398 families were predicted to target 13 and 10 genes, respectively, while the novel miRNAs were predicted to target only a few genes. For instance, the pau-miR159 family targeted genes encoding the GAMYB transcription factor and auxin response factors, the miR156 family targeted genes encoding squamosa-promoter binding-like proteins, while the pau-397a/b, pau-mR2, and pau-miR403 families targeted genes encoding serine/threonine-protein kinase abkC-like, disease resistance protein RGA2, late blight resistance protein R1-A, and protein argonaute 2, respectively. The predicted functions of these target genes were associated with plant defense, growth and development, signal transduction, and transcription.

We found that 19 of the 70 unique target genes were among the 902 DEGs detected in the PIP vs. HP and PIP-60 vs. PIP libraries (See Additional file 20: Table S20), and the relationship between miRNA and its corresponding target genes were complex, for example, Pau-miR156g was up-regulated in PIP vs. HP and down-regulated in PIP-60 vs. PIP, the target gene squamosa promoter binding protein-homologue 5 was down-regulated in PIP vs. HP and up-regulated in PIP-60 vs. PIP, while the expression level of pau-miR398a was up-regulated in PIP vs. HP and down-regulated in PIP-60 vs. PIP, the expression level of the target gene predicted protein was not change in PIP vs. HP and up-regulated in PIP-60 vs. PIP. By degradome analysis, we found the functions of these target genes were involved in plant growth and development, plant-pathogen, plant hormone, plant defense, signal transduction, and antioxidant and transcription, indicating that these complex regulation of mRNAs - miRNAs may play crucial roles in the response of *P. tomentosa* to PaWB phytoplasma.

### qRT-PCR analysis

**Fig. 2:**
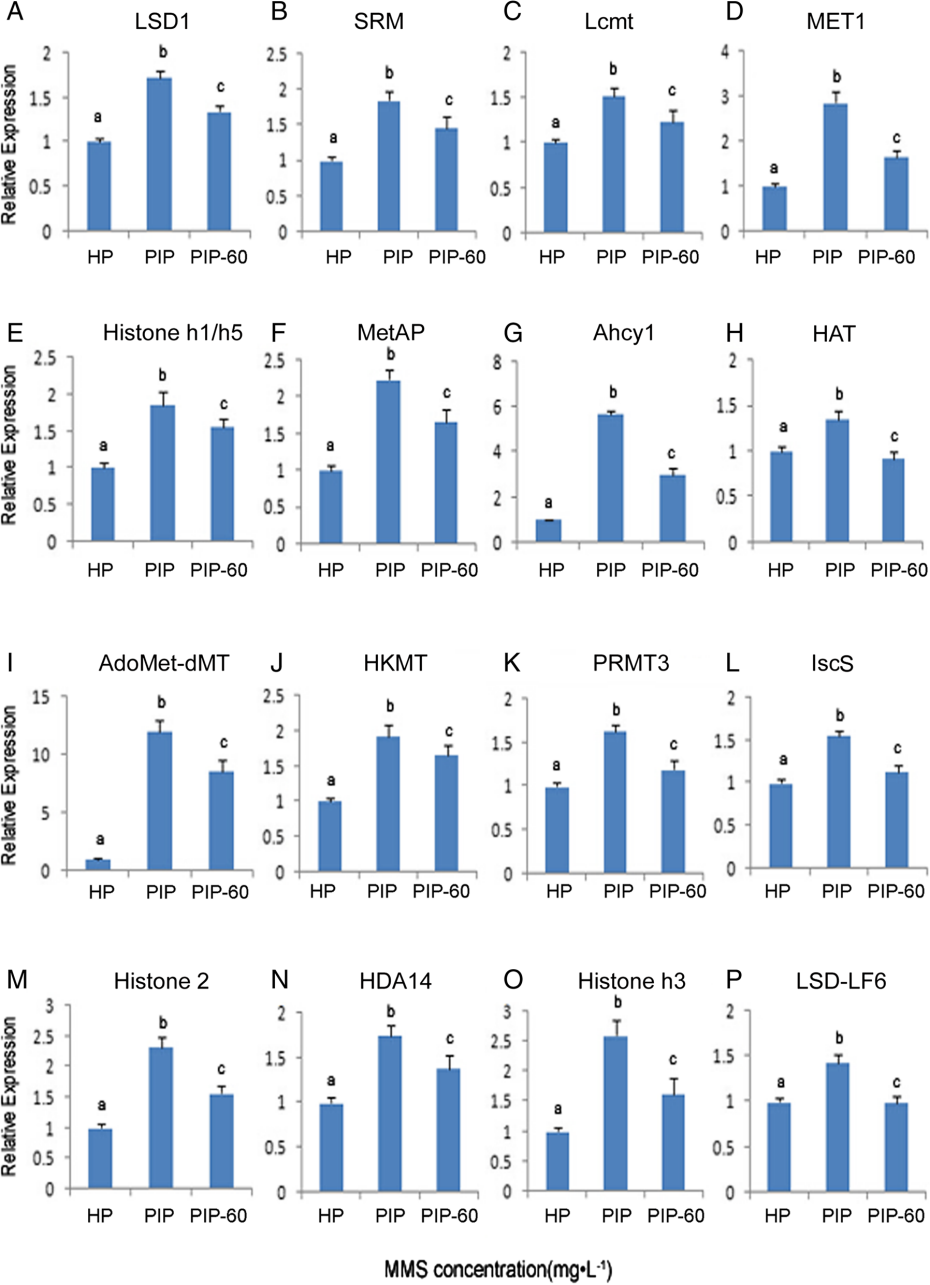
qRT-PCR analysis of *P. tomentosa* selective DEGs HP: healthy plantlets. PIP: phytoplasma-infected plantlets. PIP-60: phytoplasma infected plantlets with 60 mg · L^−1^ MMS treatment. **a**: relative expression of lysine-specific histone demethylase 1 homolog 3 (LSD1); **b**: relative expression of spermidine synthase (SRM); **c**: relative expression of leucine carboxyl methyltransferase (Lcmt); **d**: relative expression of MET1-type DNA-methyltransferase (MET1); **e**: relative expression of histone h1/h5 (Histone h1/h5); **f**: relative expression of methionine aminopeptidase (MetAP); **g**: relative expression of adenosylhomocysteinase isoform 1 (Ahcyl); **h**: relative expression of histone acetyltransferase (HAT); **i**: relative expression of S-adenosyl-L-methionine -dependent methyltransferase domain-containing protein (AdoMet-dMT); **i**: relative expression of histone-lysine N-methyltransferase (HKMT); **k**: relative expression of arginine N-methyltransferase 3 (PRMT3); **l**: relative expression of cysteine desulfurase (IscS); **m**: relative expression of histone 2 (Histone 2); **n**: relative expression of histone deacetylase 14 (HDA14); **o**: relative expression of histone H3 (Histone h3); **p**: relative expression of lysine-specific demethylase LF6-like (LSD-LF6); The different letters within a gene repression level indicate significant difference, while the same letters within a gene repression level indicate no significant differences (*p* < 0.05), the same below

**Fig. 3:**
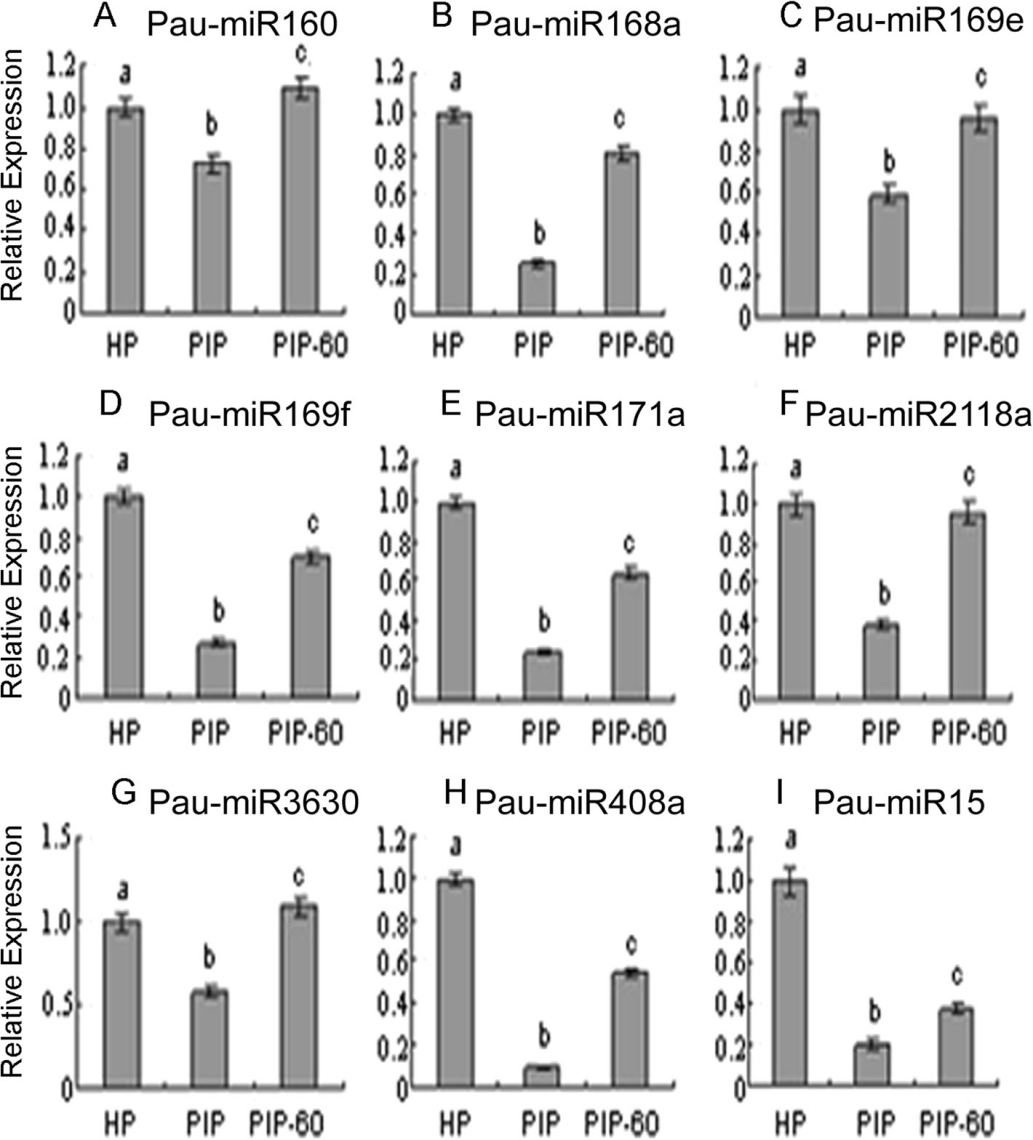
qRT-PCR analysis of *P. tomentosa* selective miRNAs. **a**: relative expression of Pau-miR160c; **b**: relative expression of Pau-miR168a; **c**: relative expression of Pau-miR169e; **d**: relative expression of Pau-miR169f; **e**: relative expression of Pau-miR171a; **f**: relative expression of Pau-miR2118a; **g**: relative expression of Pau-miR3630; **h**: relative expression of Pau-miR408a; **i**: relative expression of Pau-miR15

**Fig. 4:**
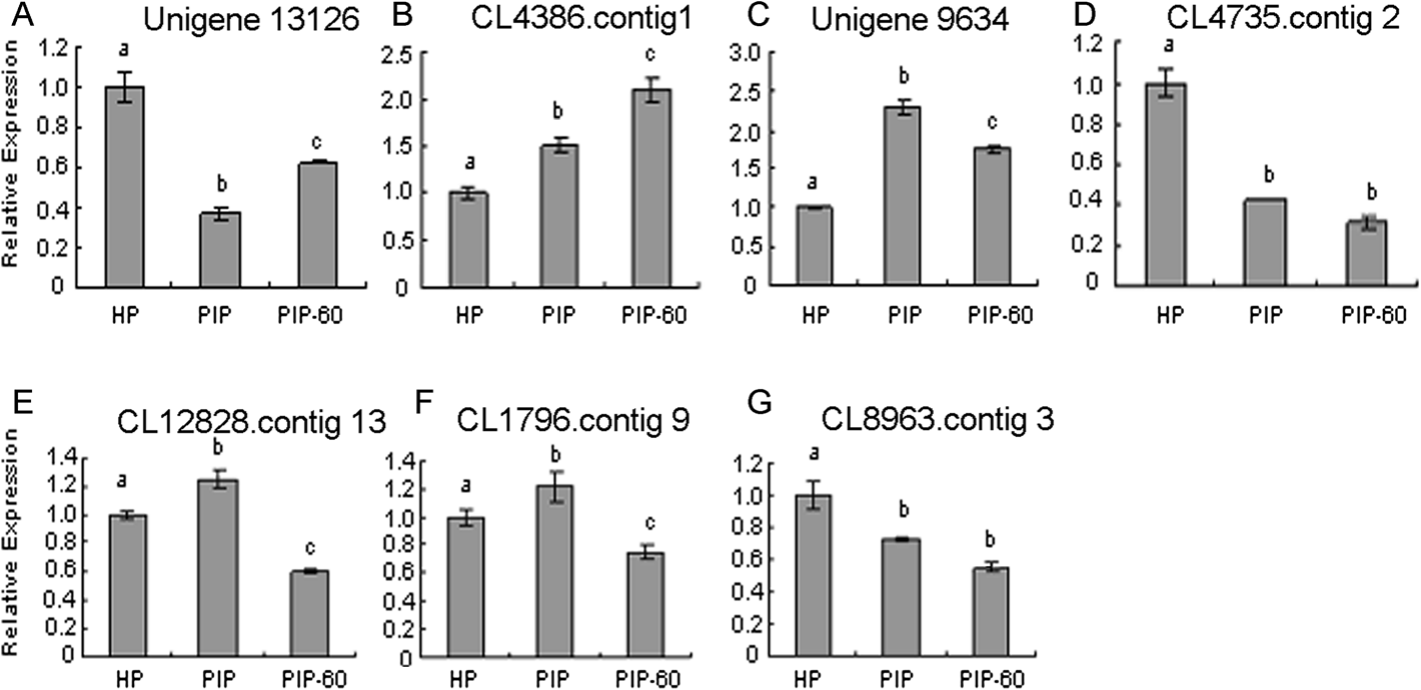
qRT-PCR analysis of *P. tomentosa* selective miRNAs target **a**: relative expression of unigene 13126; **b**: relative expression of CL4368. Contig 1; **c**: relative expression of unigene 9654; **d**: relative expression of CL4735.contig2; **e**: relative expression of CL12828.contig13; **f**: relative expression of CL1796.contig 9; **g**: relative expression of CL8963.contig 3

To verify the results of high-throughput sequencing, 16 DEGs and nine potential targets genes and the corresponding miRNAs were selected for qRT-PCR analysis. As shown in Figs. 2 and 3, the expression patterns of the DEGs and the miRNAs were consistent with those generated from high-throughput sequencing, and the expressions of seven target genes had negative correlation with the corresponding miRNAs (Fig. 4). The qRT-PCR results confirm that the transcriptomic datasets generated in this study are sufficient to assess the mRNA-miRNA interactions in the response of *P. tomentosa* to PaWB phytoplasma.

## Discussion

The mechanisms of PaWB disease may be better understood by analyzing the genome-wide gene expression profiles of healthy and infected Paulownia plants. Since this disease is difficult to control and the genomic information of paulownia has not been published, the major research work is to identify the genes response to phytoplasma infection from the host level. Currently four important achievement have been established from different varieties of paulownia, the first important achievement was our team found an effective reagent (MMS) which can recover the morphology of the seedlings with phytoplasma, and the phytoplasma specific 16SrDNA fragment cannot be detected in the seedlings with suitable MMS concentration treatment; the second is some genes showing differentially expressed in the pathway of primary and secondary metabolism, photosynthesis, cell cycle and division, cell wall biosynthesis and degradation, hormone biosynthesis, plant-pathogen interaction, circadian rhythm, phenylpropanoid metabolism, folate synthesis, and fatty acid synthesis; the third achievement is the DNA methylation level of the healthy paulownia seedlings reduced after phytoplasma infection, and the DNA methylation pattern were also changed, the fourth is some miRNA response to phytoplasma infection were obtained [26, 27, 29–34]; however, until now, integrative analyses of the regulation of mRNAs-miRNAs in Paulownia in response to phytoplasma infection have not been reported. Hence, in this study, we extended the fundamental understanding of the mechanism of PaWB by comparing mRNA- miRNA expression regulation of *P. tomentosa* plantlets infected with phytoplasma.

### Interaction of miRNAs-target genes mediated gene expression responsible for plant morphological changes

As we known, plant morphological changes involved in many regulatory factors including transcription regulation, post-transcription regulation, changes of signal transduction, modification of epigenetic, and changes of proteins. During our previous analysis, auxin efflux carrier 5NG4, as the symptom expression proteins was indentified [30], nevertheless, the miRNA associated with PaWB morphological change has not been reported. Evidence shows that miR157, which is highly conserved in plants, targets genes that encode squamosa-promoter binding proteins(SBP), and changes in the expression levels of these genes have been shown to play important roles in modifying the leaf morphology of phytoplasma- infected plants [40, 60], Another miRNA, miR156, can disturb gibberellins level and exhibit enhanced branching from axillary buds, resulting in a bushy appearance and a delay in flowering [61–63], it has also been reported miR156 combined miR157 to act together to control plant morphology by regulating the expression of the target gene encoding a DNA –binding transcription factor [60, 64]. In the present study, miR 156 was significantly induced in the PaWB-infected plantlets, and its expression level decreased in the MMS-treated plantlets (Table 3). The target gene of pau-miR156g was predicted to be squamosa-promoter binding-like protein (SPL9) by mRNA cleavage products sequencing, which are characterized by a 76-amino acid DNA-binding domain named SBP, genes of the SPL family are known to play a role in flowering regulation and phase transition [65]. Evidence showed that the interaction of miR156-SPL9 could directly activate flower- promoting MADS box genes by binding their promoters and resulted in the formation of smaller leaves, loss of apical dominance, and the initiation of more leaves with shorter plastochron lengths [66], the same regulation function was also identified in the phytoplama infected mulberry [41]. Therefore, we speculate that the miR156-SPL interaction may be one of the important regulatory factors in morphological changes, further experiment are required to verify the interaction of miR156-SPL in morphological changes observed in Paulownia in response to phytoplasma infection.

**Table 3:** Expression significantly altered miRNAs of *P. tomentosa*

Name	Fold change	*P*-value	Regulation	Fold change	*P*-value	Regulation
	PIP/HP	PIP/HP		PIP-60/PIP	PIP-60/PIP	
pau-miR156g	1.77	0.00	up*	−2.18	0.00	down*
pau-miR160a,b	1.13	0.00	up*	0.02	0.74	up
pau-miR160c	−0.65	0.22	down	1.06	0.04	up*
pau-miR166a,b	1.14	0.00	up*	−0.17	0.00	down
pau-miR166c	1.09	0.00	up*	−0.38	0.00	down
pau-miR168a,b	0.33	0.00	up	1.01	0.00	up*
pau-miR169c,d	−0.84	0.00	down	1.33	0.00	up*
pau-miR169e	−1.30	0.00	down*	1.79	0.00	up*
pau-miR169f,g	−1.48	0.00	down*	0.85	0.13	up
pau-miR171a,b	−2.58	0.06	down*	-	-	-
pau-miR390	1.20	0.00	up*	0.04	0.71	up
pau-miR396a	1.32	0.00	up*	−0.08	0.00	down
pau-miR396b	1.50	0.00	up*	−0.37	0.00	down
pau-miR397a,b	1.30	0.00	up*	−1.14	0.00	down*
pau-miR397c	−11.45	0.00	down*	-	-	-
pau-miR398a	1.06	0.00	up*	−0.79	0.00	down
pau-miR403	1.21	0.00	up*	−0.09	0.00	down
pau-miR408a-f	−1.05	0.00	down*	-	-	-
pau-miR2118a,b	−0.70	0.01	down	2.08	0.00	up*
pau-miR3630	−1.96	0.00	down*	1.81	0.00	up*
pau-mR4	5.24	0.00	up*	−1.29	0.00	down*
pau-mR9	0.59	0.00	up	1.47	0.00	up*
pau-mR15	−1.25	0.00	down*	−0.29	0.27	down
pau-mR17a,b	1.05	0.00	up*	-	0.00	-

^a-g^Representing the different miRNAs

*Indicating expression significantly changed miRNA

### Role of miRNAs-target gene mediated gene expression involved in plant defenses

Plant defenses against pathogens involve a large number of genes whose expressions are regulated at the transcriptional and post-transcriptional levels [67–70]. In the previous study of PaWB disease, we identified several genes related to plant defense, including cytochrome P450, LRR receptor-like serine/threonine-protein kinase, guanine nucleotide-exchange factor, L-ascorbate peroxidase, (S)-2-hydroxy-acid oxidase, nitric-oxide synthase, ROS, glutathione S-transferase, auxin-induced protein 5NG4, and the WRKY29 and MYC2 transcription factors [29–31]; however, the interaction of miRNAs and their corresponding target genes in the plant defense have not yet been reported. In this study, several phytoplasma- responsive miRNAs involved in defense were identified, and some of these miRNAs were predicted to target some of the previously indentified defense genes. For example miR403, was up-regulated in the PaWB-infected plantlets (PIP) and down-regulated in the HP and PIP-60 plantlets, the degradome sequencing predicted that it target a gene encoding serine/threonine-protein kinase abkC, and its expression negatively correlated with expression level of the target gene, this kinase could phosphorylate serine or threonine residues, and it can be regulated by hormones, growth factors, and cell surface receptors, as well as cellular stress. The cell surface receptors of this kinase were reported to take part in the regulation of cell proliferation and programmed cell death (apoptosis) [71, 72]. We also identified some other miRNA involved in plant defense, such as pau-mR2, although its expression levels were not obvious changed in all three libraries, target gene encoding disease resistance RPP13-like protein 1 was up-regulated in PIP, and down-regulated in the HP and PIP-60. Disease resistance RPP13-like protein 1 contains a nucleotide-binding site (NBS) and a leucine-rich repeat region (LRR), and is unique among the NBS:LRR R protein family [73], which could directly bind pathogen proteins, further modified either the host or pathogen protein and triggered conformational changes in the N-terminal and LRR domain of the NBS-LRR protein. These changes increased the conversion of ADP to ATP in the NBS domain and activated downstream signaling, further affected plant resistance [74]. The pau-miR397a, b were up-regulated in PIP and down-regulated in the HP and PIP-60. Curiously, similar expression trends also occurred in their target genes, which encodes laccase, this enzyme comprises three characteristic multicopper oxidase homologous domains, catalyzes the oxidation of a wide variety of phenolic substrates. Laccase is an oxidase that oxidizes non-phenolic substrates and to degrade lignin [75]. Lignification has been found to be one of the possible mechanisms of the active resistance of plants against pathogens [76]. Conversely, lignin degradation may play a role in the survival of some pathogens that attack plants [77]. However, the concentration of most key defense gene ROS were rapidly accumulated in the phytoplasma infected plants [30, 78], which act as direct reactive substrates to kill pathogens, to synthesize lignin and other oxidized phenolic compounds that further thicken the cell walls to forms an effective physical barrier to pathogens [79, 80], additionally, as mentioned above, the gene expression regulation of miR 156- SPL9 can negatively regulate of anthocyanin biosynthesis that are well-known to play important role in plant defense [81]. Based on analysis of the miRNAs and their targets, a potential co-regulatory network was speculated to describe the gene expression regulation in the pathological changes of paulownia seedlings with phytoplasma. There was highly complex cross-talk between diverse miRNA pathways involved in the responses of *P. tomentosa* to phytoplasma infection.

### Phytoplasma infection altered miRNAs responsive to plant hormone signaling pathways

Since the identification of changes of plant hormone as key regulatory molecules in the pathway controlling development of plant morphology, great progress has been made in the understanding of causing the development of disease symptoms in phytoplasma-infected plants [82, 83], and the research on the relationship between miRNA related to plant hormone pathways and plant witches’ broom has been reported, in phytoplasma-infected mulberry, yellow dwarf disease was found to be caused by changes in the expression of genes encoding plant hormones targeted by several differentially expressed miRNAs, including miR393a, miR160, miR164, and miR166 that regulated genes associated with the auxin signaling pathway, miR164, miR166, and miR2111a-5p that regulated genes associated with ethylene metabolism, and miR2595 and miRn20-5p that regulated genes associated with the salicylic acid pathway [41]. In phytoplasma-infected mexican lime, only changes in the expression of genes associated with auxin signal transduction changes were detected. These genes were predicted to be targeted by miR160, miR166, and miR167 [40]. In the phytoplasma- infected Paulownia plantlets, expression changes were found for genes to play important role in other plant hormones synthesis, such as *IPT*, *CYP735A*, *CISZOG*, *CRE1*, *AHP*, *PFS*, *XD, ABF*, and *CruA*, *BAK1*, *BRI1 and CYCD3* which have been shown to mediate cytokinin, brassinosteroid (BR) and abscisic acid (ABA) biosynthesis [29–31, 34].

In order to deeply understand the relationship between occurrence of PaWB and miRNA related to plant hormones, several hormones responsive-miRNAs were identified; for example, pau-miR166c was up-regulated in PaWB-infected plantlets and down-regulated in healthy plantlets. According to the degradome analysis, the predicted target gene of pau-miR166c was a homeobox-leucine zipper protein REVOLUTA (HD-ZIP) that is a transcription factor unique to plants characterized by a homeodomain and a leucine zipper motif, and belongs to HD-Zips III proteins. According to the transcriptome analysis, this target gene was also differentially expressed in the PaWB-infected plantlets and the healthy plantlets. This probably be responsible for the abnormal plant development such as meristem defects and was partially responsible for the symptoms such as dwarf and witches’ broom [41]. HD-Zips also involved in ABA signaling pathway [84]. In addition, previous observations showed that the content of ABA increased in the PaWB-infected plantlets [29–31], and the high ABA content can induce HD-Zips and reduced phosphorylation of ribosomal protein S6, meanwhile, the expression of expansin A10 (EXPA10) and DWARF4 (DWF4) also increased, which affected the leaf size [85]. Over-expression of DWF4 also was reported to affect the BR biosynthetic pathway [86]. Once the BR signal was recognized by the plant, the NADPH oxidase and ROS were induced, which initiated a protein phosphorylation cascade and a hypersensitive response in the plant [40, 68].

Beside the ABA and BR signaling pathway, some of the miRNAs identified in the present study were also involved in regulating other hormone signaling pathways. For example, differential expression of miR159 was associated with the auxin signaling pathway, nevertheless, there were no changes in the expressions of the target genes in the HP and PIP-60 plantlets, but one of the targets genes, auxin response factor 18 (ARF18), was significantly up-regulated in the PIP, which play an important role in the early auxin response [87], and activation of auxin signaling might promote susceptiability to plant disease by providing carbon and nitrogen sources for pathogens [40]. Pathogen-infected plants, also developed several counter measures to suppress auxin signaling, such as miR160 that is identified in the current study, regulated the expression of a set of signaling genes that were involved in various steps of auxin signaling and indirectly restrained the pathogen growth 88]. Therefore, the formation of plant symptoms might associate with many hormones pathways coordinately. Taken together of these findings, our observations contribute to the evidence that the formation of the PaWB symptoms may be related to the common regulation of many genes and miRNAs associated with plant hormones.

## Conclusions

In this study, we obtained integrated PaWB-responsive miRNA- target genes regulation profiles of *P. tomentosa* in the RNA samples (HP, PIP, and PIP-60) by combing three high-throughput sequencing methods. A total of 98,714 all-unigenes and 100 miRNAs were identified in these samples. Among them, 902 DEGs and 24 miRNAs responsive to PaWB phytoplasmas were discovered by comparing the changes of gene expression between PIP vs. HP and PIP-60 vs. PIP. Targets of these miRNAs were surveyed based on the transcriptome sequencing, and 19 of the target genes were found among the previously identified DEGs. The functions of the target genes indicated that they were associated mainly with morphological changes, plant defense, and plant hormones. Important miRNAs-target genes interactions, such as pau-miR156g-SPL, pau-miR403-serine/threonine- protein kinase abkC, and pau-miR166c-HD-ZIP were the main regulation associated with the occurrence of PaWB symptoms. Future studies will be focused on exploring the function of these phytoplasma-responsive miRNA, and identifying the pathways connected with the cause of witches’ broom symptom. These datasets, which include the predicted biochemical functions of the DEGs and miRNAs in the three samples, will be particularly useful for understanding the responses of Paulownia and other plant species to phytoplasma infection.

## Ethical state

This article does not contain any studies with human participants or animals performed by any of the authors.

## Availability of supporting data

All sequencing data generated in this study is available from the SRA-Archive (http://www.ncbi.nlm.nih.gov/sra) under the study accession SRP031625, SRP058902, SRP060300 and SRP060876. We performed three sequencing approaches and set up three experiments, respectively, for transcriptome sequencing, the accession number are SRX1013178, SRX1013200 and SRX1013201; for miRNA sequencing, the accession number are SRX1080538, SRX1080540 and SRX1081902; for degradome sequence, the accession number are SRX1093864, SRX1093863 and SRX1093900.

## Additional files

Additional file 1: Table S1. Primers of *P. tomentosa* DEGs for qRT-PCR analysis. (DOCX 35.5 kb) Additional file 2: Table S2. Primers of *P. tomentosa* miRNAs for qRT-PCR analysis. (DOCX 20.7 kb) Additional file 3: Table S3. Primers of *P. tomentosa* miRNA target gene for qRT-PCR analysis. (DOCX 22.6 kb) Additional file 4: Table S4 Overview of the transcriptome sequencing and assembly of *P. tomentosa*. (DOCX 29.3 kb) Additional file 5: Table S5. E-value statistic of the all-unigenes *: the number of all-unigenes that satisfied the corresponding E-value. (DOCX 19.6 kb) Additional file 6: Table S6. Similarity statistic of the all-unigenes *: the number of all-unigenes that satisfied the corresponding scope of similarity. (DOCX 19.7 kb) Additional file 7: Table S7. Species statistic of the all-unigenes *: the number of all-unigenes that hit these species and obtained the highest top scores in a blastx search. (DOCX 21.6 kb) Additional file 8: Table S8. GO function classification of all-unigenes of *P. tomentosa*. (DOCX 33.8 kb) Additional file 9: Table S9. COG function classification of all-unigenes of *P. tomentosa*. (DOCX 27.3 kb) Additional file 10: Table S10. KEGG pathway analysis of all-unigene for *P. tomentosa* *: the number of the all-unigenes involved in the corresponding pathway. (DOCX 34.7 kb) Additional file 11: Table S11. Length distribution of P. tomentosa small RNAs obtained by high-throughput sequencing in HP libraries a: Nucleotide bias at A position of sRNA tags; b: Nucleotide bias at U position of sRNA tags; c: Nucleotide bias at C position of sRNA tags; d: Nucleotide bias at G position of sRNA tags. (DOCX 29.9 kb) Additional file 12: Table S12. Length distribution of P. tomentosa small RNAs obtained by high-throughput sequencing in PIP libraries. (DOCX 30.5 kb) Additional file 13: Table S13. Length distribution of P. tomentosa small RNAs obtained by high-throughput sequencing in PIP-60 libraries. (DOCX 31.3 kb) Additional file 14: Table S14. Conserved miRNAs of P. tomentosa ath/aly: Arabidopsis; ptc: Populus trichocarp; osa: Oryza sativa; zma: Zea mays; gma: Glycine max;+: 2 mismatches; ++: 1 mismatches; +++: 0 mismatch; -: not exist. (XLS 51.0 kb) Additional file 15: Table S15. Novel miRNAs of *P. tomentosa*. (XLS 38.5 kb) Additional file 16: Table S16. Identified targets of miRNA involved in Paulownia by miRNA and degradome analysis. (XLSX 35.2 kb) Additional file 17: Table S17. Changes of gene expression of the DEGs in the three samples. (XLSX 415 kb) Additional file 18: Table S18. GO analysis of the *P. tomentosa* DEGs. (XLSX 57.7 kb) Additional file 19: Table S19. KEGG pathway analysis of the P. tomentosa DEGs a: the number of the differentially expressed unigenes that involved the corresponding pathway; b: the number of the all-unigene that involved the corresponding pathway; c: indicating a significantly enriched GO terms among the differentially expressed unigenes; d: indicating a significantly enriched pathway among the differentially expressed unigenes. (DOCX 34.0 kb) Additional file 20: Table S20. Targets of miRNA matched to DEGs. (XLSX 15.6 kb) Additional file 21: Figure S1. Length distribution of all-unigene in *P. tomentosa*. (TIF 5.5 MB) Additional file 22: Figure S2. Correlation coefficients of the gene expression of duplicate samples. Y-axis represents the logarithmic value of HP expression, while X-axis represents the logarithmic value of the corresponding duplicate samples. (TIF 3.21 MB) Additional file 23: Figure S3. COG function classification of all-unigene of *P. tomentosa*. (TIF 12.2 MB) Additional file 24: Figure S4. Hairpin structures of P. tometosa miRNAs Pau-mR1a-i have the same mature miRNA sequence; pau-mR3a/b have the same mature miRNA sequence; pau-mR14a/b have the same mature miRNA sequence; pau-mR16a-c have the same mature miRNA sequence; pau-mR17a/b have the same mature miRNA sequence; pau-mR20a/b have the same mature miRNA sequence. (DOC 1.61 MB) Additional file 25: Figure S5. DEG analysis of *P. tomentosa* a: DEGs in PIP vs. HP up-regulated and down-regulated in PIP-60 vs. PIP. b: DEGs in PIP vs. HP down-regulated and up-regulated in PIP-60 vs. PIP. (TIF 39.7 kb)
