# Supplementary material for: Transcriptome, microRNA, and degradome analyses of the gene expression of Paulownia with phytoplamsa
Source: BMC Genomics. 2015 Nov 4;16:896. doi: 10.1186/s12864-015-2074-3 (PMC4634154; doi:10.1186/s12864-015-2074-3)
Supplement: Additional file 1: Table S1. — Primers of P. tomentosa DEGs for qRT-PCR analysis. (DOCX 35.5 kb) [file 12864_2015_2074_MOESM1_ESM.docx]

**Additional file 1: Table S1 Primers of *P. tomentosa* DEGs for qRT-PCR analysis**

| Gene name | Potential gene function | Forward primer sequence (5‘ - 3’) | Reverse primer sequence (5‘ - 3’) |
| --- | --- | --- | --- |
| 18S |  | ACATAGTAAGGATTGACAGA | TAACGGAATTAACCAGACA |
| MB.Unigene16166 | Cysteine proteinase inhibitor CPI-1 | CGTTCTTCACTTCCGTTCG | TCCTCCCTTTCTTGGTAATGG |
| MB.CL2565 | MET1-type DNA-methyltransferase | CATCTGTCCTCTGGTCTG | CTATGTCTATGTGAGTCCTTAC |
| MB.CL4211 | Adenosylhomocysteinase isoform 1 | GCAAGGACCAGGCTGATTAC | TTCCCGATTCAATTTCTGTCTTTC |
| MB.CL1534 | Spermidine synthase | CGCACTCACACTCTCACTG | GCCGTTATTCACTTCCATATCG |
| MB.CL532 | Cystathionine beta-lyase | AGCACGCCTCTATATCAAACAG | CCATTCCAACAAGATGAGTAACG |
| MB.Unigene2470 | Cystathionine beta-synthase (CBS) protein | GCACATCCTGTATCTGAG | CTTGGCTTGAACACTTAC |
| MB.CL4336 | S-adenosyl-L-methionine-dependent methyltransferase domain-containing protein | GTGAGTGGAGGGCTAATG | GGAAGTTGAATGTGTCTACC |
| MB.CL3130 | Methionine aminopeptidase | TAGCCAAGGAAACACATTAGGG | GCCAGACATTTACCATTGAACC |
| MB.CL5072 | Cysteine desulfurase | GGAGAACAGGTGCTTGGAC | GGAAGAAGGCTGGCTATGC |
| MB.Unigene33695 | N6-adenosine-methyltransferase MT-A70-like | CAAGGAATGCGGCTGGTAG | AGTTATCGGTGGTGATGAAGG |
| MB.Unigene39495 | HSP26 | GCGAAAGCAAAGATACATC | ATGGCATCCTCAAATAGC |
| MB.CL8557 | Histone 2 | TTCTTGTCCTTCTCCTTGTTAGC | TCTCCTCTCCAGTTCCATTCC |
| MB.CL2878 | Histone h1/h5 | CCACTCACCCTCCTTACTTC | TTCGGCGGCAGATTCTTC |
| MB.Unigene17695 | Histone H2A | TGTGAGGAACGATGAGGAG | AACCAAATCTATCTGAAACTAAGC |
| MB.CL670 | Histone H3 | AATCGCACAGAGGTTAGTATC | GTTCCAGAGGCTTGTTCG |
| MB.Unigene26653 | Histone H4 | TCGCCGCAAGACAGTGAC | CTATGAACCTAACCGCCAAAGC |
| MB.Unigene26929 | Histone H2B | AAGAAGAAGAAGGCGAAGAAG | CGAAGATTCCTGAGCAAGC |
| MB.CL2282 | Leucine carboxyl methyltransferase | GTAACCTGAGACATCCAAG | TCCTCTACAGCAACAACC |
| MB.CL4216 | Histone acetyltransferase | TGTGACTATCGGCAGAAGAAG | CTCCTGTTCATTGACCTCTCC |
| MB.CL6179 | Histone deacetylase 14 | CCTTCACCATCTCCGCTACCTATC | GCTGTTGCTGCTCGCTATGC |
| MB.CL4350 | Histone-lysine N-methyltransferase | CCTCAACGCAACATCTTC | AGAATTTGGCTCTTACTATCG |
| MB.CL1013 | Lysine-specific histone demethylase 1 homolog 3 | AGATGATGAAGACTTGTGGCTGTG | CTGGTGGGAAGAAAGGGTTTGG |
| MB.CL6841 | Lysine-specific demethylase LF6-like | ACCGTGATGTGATTGACCAGATG | AGGAGGAGGAGGAGGAAGAGG |
| MB.CL4602 | Arginine N-methyltransferase 3 | GAACGCAATACGGCATTAC | CGCACAGTCACAGAACAG |
