# Supplementary material for: Transcriptome, microRNA, and degradome analyses of the gene expression of Paulownia with phytoplamsa
Source: BMC Genomics. 2015 Nov 4;16:896. doi: 10.1186/s12864-015-2074-3 (PMC4634154; doi:10.1186/s12864-015-2074-3)
Supplement: Additional file 2: Table S2. — Primers of P. tomentosa miRNAs for qRT-PCR analysis. (DOCX 20.7 kb) [file 12864_2015_2074_MOESM2_ESM.docx]

**Additional file 2:Table S2 Primers of *P. tomentosa* miRNA for qRT-PCR analysis**

| Gene name | | primer sequence (5‘ - 3’) |  |
| --- | --- | --- | --- |
| pau-miR160c | TCGGACCAGGCTTCATTCCCC | | |
| pau-miR168a | GGCAAGTTGTCTTTGGCTACA | | |
| pau-miR169e | TAGCCAAGGATGACTTGCCTG | | |
| pau-miR169f | TGATTGAGCCGCGCCAATATC | | |
| pau-miR171a | GCTGCCGACTCATTCATTCAA | | |
| pau-miR397a | TGTGTTCTCAGGTCGCCCCTG | | |
| pau-miR398a | TTAGATTCACGCACAAACTCG | | |
| pau-miR403 | ATGCACTGCCTCTTCCCTGGC | | |
| pau-miR408a | TTTCCAACTCCACCCATTCCTA | | |
| pau-miR2118a | TTGGGAATCTCTCTGATGCAT | | |
| pau-miR3630 | CCTGCCTTGCATCAACTGAAT | | |
| pau-mR15 | TCATTAACGCTGCATTCAATA | | |
