# Supplementary material for: Transcriptome, microRNA, and degradome analyses of the gene expression of Paulownia with phytoplamsa
Source: BMC Genomics. 2015 Nov 4;16:896. doi: 10.1186/s12864-015-2074-3 (PMC4634154; doi:10.1186/s12864-015-2074-3)
Supplement: Additional file 3: Table S3. — Primers of P. tomentosa miRNA target gene for qRT-PCR analysis. (DOCX 22.6 kb) [file 12864_2015_2074_MOESM3_ESM.docx]

**Additional file 3: Table S3 Primers of *P. tomentosa* miRNA target gene for qRT-PCR analysis**

| Gene name | Potential gene function | Forward primer sequence (5‘ - 3’) | Reverse primer sequence (5‘ - 3’) |
| --- | --- | --- | --- |
| 18S |  | ACATAGTAAGGATTGACAGA | TAACGGAATTAACCAGACA |
| CL12828 | AGO1A | TTCCTGGTGCTGGTGAGAG | TTGCGGCTGTGGTTGTTG |
| CL1796 | uncharacterized protein LOC101253139 | CCATTCCTCCATCTCTTG | AGCCTTATTCAGACTTCC |
| CL8963 | laccase | GGCTTCCTCAGTTACCAG | CACCAGTCTTGTTCCATTC |
| CL4735 | serine/threonine-protein kinase abkC | GAAGAGAGAATCCACCCAATC | GGAACAAGCATAACTAACATCG |
| Unigene13126 | Cucumber peeling cupredoxin | CAACTCCACCACAACCACTC | TTCAACTGTCCAATGTCAATGC |
| CL4368 | AGO2A2 | AAGAGGTTGTGATGGAGAC | ACTGTGAATAGCCGAAGG |
| Unigene9654 | basic blue protein | AATGTGGTGGCGGTGGAC | CAGCAGCGACAGCAATCTTC |
