# Supplementary material for: Transcriptome, microRNA, and degradome analyses of the gene expression of Paulownia with phytoplamsa
Source: BMC Genomics. 2015 Nov 4;16:896. doi: 10.1186/s12864-015-2074-3 (PMC4634154; doi:10.1186/s12864-015-2074-3)
Supplement: Additional file 4: Table S4 — Overview of the transcriptome sequencing and assembly of P. tomentosa. (DOCX 29.3 kb) [file 12864_2015_2074_MOESM4_ESM.docx]

**Additional file 4: Table S4 Overview of the transcriptome sequencing and assembly of *P. tomentosa***

| **Statistics of data production** | **HP** | **PIP** | **PIP-60** |
| --- | --- | --- | --- |
| Number of clean reads | 48，006，640 | 75，244，384 | 58，962，324 |
| Total nucleotides (nt) | 4，800，664，000 | 7，524，438，400 | 5，896，232，400 |
| Q20 percentage(%) | 0 | 0 | 0 |
| N percentage(%) | 0.08 | 0.11 | 0.03 |
| GC percentage (%) | 46.59 | 46.55 | 46.84 |
| **Contigs** | **HP** | **PIP** | **PIP-60** |
| Number of contigs | 126,285 | 134,791 | 164,905 |
| Total nucleotidesin contigs (nt) | 46,447,996 | 49,534,300 | 59,840,797 |
| N50 (bp) | 750 | 785 | 666 |
| Mean length of contigs (nt) | 368 | 367 | 363 |
| **Unigenes** | **HP** | **PIP** | **PIP-60** |
| Number of unigenes | 77,838 | 79,442 | 112,589 |
| Total nucleotides in unigenes(nt) | 60,706,392 | 67,405,338 | 106,294,005 |
| N50 (bp) | 1514 | 1618 | 1847 |
| Mean length of unigenes (bp) | 780 | 848 | 944 |
| **All unigenes** |  |  |  |
| Number of all unigenes | 98,714 |  |  |
| Total nucleotides in all unigenes(nt) | 111,888,740 |  |  |
| N50 (bp) | 1875 |  |  |
| Mean length of all unigenes (bp) | 1133 |  |  |
