# Supplementary material for: Transcriptome, microRNA, and degradome analyses of the gene expression of Paulownia with phytoplamsa
Source: BMC Genomics. 2015 Nov 4;16:896. doi: 10.1186/s12864-015-2074-3 (PMC4634154; doi:10.1186/s12864-015-2074-3)
Supplement: Additional file 5: Table S5. — E-value statistic of the all-unigenes *: the number of all-unigenes that satisfied the corresponding E-value. (DOCX 19.6 kb) [file 12864_2015_2074_MOESM5_ESM.docx]

**Additional file 5: Table S5 E-value statistic of the all-unigenes**

| E-value | Gene numbers* | Percentage |
| --- | --- | --- |
| 0 | 12759 | 20.03% |
| 0~1e-100 | 13239 | 20.79% |
| 1e-100~1e-60 | 9737 | 15.29% |
| 1e-60~1e-45 | 4840 | 7.60% |
| 1e-45~1e-30 | 6615 | 10.39% |
| 1e-30~1e-15 | 8567 | 13.45% |
| 1e-15~1e-5 | 7928 | 12.45% |

*

*: the number of all-unigenes that satisfied the corresponding E-value.
