# Supplementary material for: Transcriptome, microRNA, and degradome analyses of the gene expression of Paulownia with phytoplamsa
Source: BMC Genomics. 2015 Nov 4;16:896. doi: 10.1186/s12864-015-2074-3 (PMC4634154; doi:10.1186/s12864-015-2074-3)
Supplement: Additional file 6: Table S6. — Similarity statistic of the all-unigenes *: the number of all-unigenes that satisfied the corresponding scope of similarity. (DOCX 19.7 kb) [file 12864_2015_2074_MOESM6_ESM.docx]

**Additional file 6: Table S6 Similarity statistic of the all-unigenes**

| Similarity | Gene numbers* | Percentage |
| --- | --- | --- |
| 19%~40% | 4163 | 6.54% |
| 40%~60% | 15640 | 24.56% |
| 60%~80% | 29311 | 46.02% |
| 80%~95% | 13252 | 20.81% |
| 95%~100% | 1319 | 2.07% |

*: the number of all-unigenes that satisfied the corresponding scope of similarity.
