# Supplementary material for: Transcriptome, microRNA, and degradome analyses of the gene expression of Paulownia with phytoplamsa
Source: BMC Genomics. 2015 Nov 4;16:896. doi: 10.1186/s12864-015-2074-3 (PMC4634154; doi:10.1186/s12864-015-2074-3)
Supplement: Additional file 7: Table S7. — Species statistic of the all-unigenes *: the number of all-unigenes that hit these species and obtained the highest top scores in a blastx search. (DOCX 21.6 kb) [file 12864_2015_2074_MOESM7_ESM.docx]

**Additional file 7: Table S7 Species statistic of the all-unigenes**

| Similarity | Gene numbers* | Percentage |
| --- | --- | --- |
| Vitis vinifera | 32394 | 50.87% |
| Ricinus communis | 8192 | 12.86% |
| Populus trichocarpa | 6991 | 10.98% |
| Glycine max | 3663 | 5.75% |
| Nicotiana tabacum | 1211 | 1.90% |
| Medicago truncatula | 1150 | 1.81% |
| Solanum lycopersicum | 1127 | 1.77% |
| other | 8957 | 14.06% |

*: the number of all-unigenes that hit these species and obtained the highest top scores in a blastx search.
