# Supplementary material for: Transcriptome, microRNA, and degradome analyses of the gene expression of Paulownia with phytoplamsa
Source: BMC Genomics. 2015 Nov 4;16:896. doi: 10.1186/s12864-015-2074-3 (PMC4634154; doi:10.1186/s12864-015-2074-3)
Supplement: Additional file 8: Table S8. — GO function classification of all-unigenes of P. tomentosa. (DOCX 33.8 kb) [file 12864_2015_2074_MOESM8_ESM.docx]

**Additional file 8:** [**Table S8**](http://www.plosone.org/article/info:doi/10.1371/journal.pone.0086976#pone-0086976-g002) **GO function classification of all-unigenes of *P. tomentosa***

| Subgroup | Class | Numbers of All-Unigene |
| --- | --- | --- |
|  | biological adhesion | 445 |
|  | biological regulation | 14680 |
|  | carbon utilization | 28 |
|  | cell proliferation | 698 |
|  | cellular component organization or biogenesis | 10633 |
|  | cellular process | 34045 |
|  | death | 1286 |
|  | developmental process | 10572 |
|  | establishment of localization | 9346 |
|  | growth | 2435 |
| biological_process | immune system process | 2379 |
|  | localization | 9924 |
|  | locomotion | 64 |
|  | metabolic process | 32341 |
|  | multicellular organismal process | 10429 |
|  | negative regulation of biological process | 3324 |
|  | multi-organism process | 4668 |
|  | positive regulation of biological process | 3178 |
|  | reproductive process | 6407 |
|  | signaling | 5191 |
|  | reproduction | 6425 |
|  | single-organism process | 13965 |
|  | response to stimulus | 17344 |
|  | cell junction | 2182 |
|  | cell | 42432 |
|  | cell part | 42432 |
|  | extracellular matrix | 45 |
|  | extracellular matrix part | 11 |
|  | extracellular region | 3066 |
|  | extracellular region part | 55 |
| cellular_component | macromolecular complex | 4945 |
|  | membrane | 17135 |
|  | membrane part | 6099 |
|  | membrane-enclosed lumen | 2036 |
|  | nucleoid | 107 |
|  | organelle | 34488 |
|  | organelle part | 9703 |
|  | symplast | 2164 |
|  | virion | 1 |
|  | virion part | 1 |
|  | antioxidant activity | 248 |
|  | binding | 24517 |
|  | catalytic activity | 25150 |
|  | channel regulator activity | 10 |
|  | electron carrier activity | 715 |
|  | enzyme regulator activity | 647 |
|  | metallochaperone activity | 10 |
| molecular_function | molecular transducer activity | 1029 |
|  | nucleic acid binding transcription factor activity | 1394 |
|  | nutrient reservoir activity | 30 |
|  | protein binding transcription factor activity | 176 |
|  | protein tag | 6 |
|  | receptor activity | 408 |
|  | structural molecule activity | 979 |
|  | translation regulator activity | 20 |
|  | transporter activity | 3977 |
