# Supplementary material for: Transcriptome, microRNA, and degradome analyses of the gene expression of Paulownia with phytoplamsa
Source: BMC Genomics. 2015 Nov 4;16:896. doi: 10.1186/s12864-015-2074-3 (PMC4634154; doi:10.1186/s12864-015-2074-3)
Supplement: Additional file 9: Table S9. — COG function classification of all-unigenes of P. tomentosa. (DOCX 27.3 kb) [file 12864_2015_2074_MOESM9_ESM.docx]

**Additional file 9:** [**Table S9**](http://www.plosone.org/article/info:doi/10.1371/journal.pone.0086976#pone-0086976-g002) **COG function classification of all-unigenes of *P. tomentosa***

| Code | COG Functional-Categories | Gene-Number |
| --- | --- | --- |
| A | RNA processing and modification | 415 |
| B | Chromatin structure and dynamics | 509 |
| C | Energy production and conversion | 1274 |
| D | Cell cycle control, cell division, chromosome partitioning | 1899 |
| E | Amino acid transport and metabolism | 2225 |
| F | Nucleotide transport and metabolism | 439 |
| G | Carbohydrate transport and metabolism | 3220 |
| H | Coenzyme transport and metabolism | 978 |
| I | Lipid transport and metabolism | 1244 |
| J | Translation, ribosomal structure and biogenesis | 2805 |
| K | Transcription | 4777 |
| L | Replication, recombination and repair | 4464 |
| M | Cell wall/membrane/envelope biogenesis | 2202 |
| N | Cell motility | 341 |
| O | Posttranslational modification, protein turnover, chaperones | 3478 |
| P | Inorganic ion transport and metabolism | 1445 |
| Q | Secondary metabolites biosynthesis, transport and catabolism | 1431 |
| R | General function prediction only | 9174 |
| S | Function unknown | 2463 |
| T | Signal transduction mechanisms | 3825 |
| U | Intracellular trafficking, secretion, and vesicular transport | 1120 |
| V | Defense mechanisms | 717 |
| W | Extracellular structures | 20 |
| Y | Nuclear structure | 7 |
| Z | Cytoskeleton | 715 |
