# Supplementary material for: Transcriptome, microRNA, and degradome analyses of the gene expression of Paulownia with phytoplamsa
Source: BMC Genomics. 2015 Nov 4;16:896. doi: 10.1186/s12864-015-2074-3 (PMC4634154; doi:10.1186/s12864-015-2074-3)
Supplement: Additional file 10: Table S10. — KEGG pathway analysis of all-unigene for P. tomentosa *: the number of the all-unigenes involved in the corresponding pathway. (DOCX 34.7 kb) [file 12864_2015_2074_MOESM10_ESM.docx]

**Additional file 10: Table S10 KEGG pathway analysis of all-unigene for *P. tomentosa***

| # | KEGG Pathway | Count* | Pathway ID |
| --- | --- | --- | --- |
| 1 | [Metabolic pathways](file:///E:\cao\MB%20转录组\MB60二四倍差异\MB60\F13TSHCCKW0004\annotation\KEGG\All-Unigene.fa.htm#gene1) | 8420 | ko01100 |
| 2 | [Biosynthesis of secondary metabolites](file:///E:\cao\MB%20转录组\MB60二四倍差异\MB60\F13TSHCCKW0004\annotation\KEGG\All-Unigene.fa.htm#gene2) | 4041 | ko01110 |
| 3 | [Plant-pathogen interaction](file:///E:\cao\MB%20转录组\MB60二四倍差异\MB60\F13TSHCCKW0004\annotation\KEGG\All-Unigene.fa.htm#gene3) | 2212 | ko04626 |
| 4 | [Plant hormone signal transduction](file:///E:\cao\MB%20转录组\MB60二四倍差异\MB60\F13TSHCCKW0004\annotation\KEGG\All-Unigene.fa.htm#gene4) | 2012 | ko04075 |
| 5 | [Spliceosome](file:///E:\cao\MB%20转录组\MB60二四倍差异\MB60\F13TSHCCKW0004\annotation\KEGG\All-Unigene.fa.htm#gene5) | 1545 | ko03040 |
| 6 | [RNA transport](file:///E:\cao\MB%20转录组\MB60二四倍差异\MB60\F13TSHCCKW0004\annotation\KEGG\All-Unigene.fa.htm#gene6) | 1224 | ko03013 |
| 7 | [Glycerophospholipid metabolism](file:///E:\cao\MB%20转录组\MB60二四倍差异\MB60\F13TSHCCKW0004\annotation\KEGG\All-Unigene.fa.htm#gene7) | 1139 | ko00564 |
| 8 | [Endocytosis](file:///E:\cao\MB%20转录组\MB60二四倍差异\MB60\F13TSHCCKW0004\annotation\KEGG\All-Unigene.fa.htm#gene8) | 1107 | ko04144 |
| 9 | [Protein processing in endoplasmic reticulum](file:///E:\cao\MB%20转录组\MB60二四倍差异\MB60\F13TSHCCKW0004\annotation\KEGG\All-Unigene.fa.htm#gene9) | 1069 | ko04141 |
| 10 | [Starch and sucrose metabolism](file:///E:\cao\MB%20转录组\MB60二四倍差异\MB60\F13TSHCCKW0004\annotation\KEGG\All-Unigene.fa.htm#gene10) | 959 | ko00500 |
| 11 | [Ether lipid metabolism](file:///E:\cao\MB%20转录组\MB60二四倍差异\MB60\F13TSHCCKW0004\annotation\KEGG\All-Unigene.fa.htm#gene11) | 859 | ko00565 |
| 12 | [Ubiquitin mediated proteolysis](file:///E:\cao\MB%20转录组\MB60二四倍差异\MB60\F13TSHCCKW0004\annotation\KEGG\All-Unigene.fa.htm#gene12) | 789 | ko04120 |
| 13 | [Ribosome](file:///E:\cao\MB%20转录组\MB60二四倍差异\MB60\F13TSHCCKW0004\annotation\KEGG\All-Unigene.fa.htm#gene13) | 705 | ko03010 |
| 14 | [Purine metabolism](file:///E:\cao\MB%20转录组\MB60二四倍差异\MB60\F13TSHCCKW0004\annotation\KEGG\All-Unigene.fa.htm#gene14) | 703 | ko00230 |
| 15 | [mRNA surveillance pathway](file:///E:\cao\MB%20转录组\MB60二四倍差异\MB60\F13TSHCCKW0004\annotation\KEGG\All-Unigene.fa.htm#gene15) | 688 | ko03015 |
| 16 | [RNA degradation](file:///E:\cao\MB%20转录组\MB60二四倍差异\MB60\F13TSHCCKW0004\annotation\KEGG\All-Unigene.fa.htm#gene16) | 633 | ko03018 |
| 17 | [Pyrimidine metabolism](file:///E:\cao\MB%20转录组\MB60二四倍差异\MB60\F13TSHCCKW0004\annotation\KEGG\All-Unigene.fa.htm#gene17) | 592 | ko00240 |
| 18 | [Ribosome biogenesis in eukaryotes](file:///E:\cao\MB%20转录组\MB60二四倍差异\MB60\F13TSHCCKW0004\annotation\KEGG\All-Unigene.fa.htm#gene18) | 581 | ko03008 |
| 19 | [Phenylpropanoid biosynthesis](file:///E:\cao\MB%20转录组\MB60二四倍差异\MB60\F13TSHCCKW0004\annotation\KEGG\All-Unigene.fa.htm#gene19) | 574 | ko00940 |
| 20 | [ABC transporters](file:///E:\cao\MB%20转录组\MB60二四倍差异\MB60\F13TSHCCKW0004\annotation\KEGG\All-Unigene.fa.htm#gene20) | 514 | ko02010 |
| 21 | [Glycolysis / Gluconeogenesis](file:///E:\cao\MB%20转录组\MB60二四倍差异\MB60\F13TSHCCKW0004\annotation\KEGG\All-Unigene.fa.htm#gene21) | 483 | ko00010 |
| 22 | [Circadian rhythm - plant](file:///E:\cao\MB%20转录组\MB60二四倍差异\MB60\F13TSHCCKW0004\annotation\KEGG\All-Unigene.fa.htm#gene22) | 448 | ko04712 |
| 23 | [Pentose and glucuronate interconversions](file:///E:\cao\MB%20转录组\MB60二四倍差异\MB60\F13TSHCCKW0004\annotation\KEGG\All-Unigene.fa.htm#gene23) | 419 | ko00040 |
| 24 | [Amino sugar and nucleotide sugar metabolism](file:///E:\cao\MB%20转录组\MB60二四倍差异\MB60\F13TSHCCKW0004\annotation\KEGG\All-Unigene.fa.htm#gene24) | 404 | ko00520 |
| 25 | [Oxidative phosphorylation](file:///E:\cao\MB%20转录组\MB60二四倍差异\MB60\F13TSHCCKW0004\annotation\KEGG\All-Unigene.fa.htm#gene25) | 396 | ko00190 |
| 26 | [Galactose metabolism](file:///E:\cao\MB%20转录组\MB60二四倍差异\MB60\F13TSHCCKW0004\annotation\KEGG\All-Unigene.fa.htm#gene26) | 375 | ko00052 |
| 27 | [Phosphatidylinositol signaling system](file:///E:\cao\MB%20转录组\MB60二四倍差异\MB60\F13TSHCCKW0004\annotation\KEGG\All-Unigene.fa.htm#gene27) | 361 | ko04070 |
| 28 | [Peroxisome](file:///E:\cao\MB%20转录组\MB60二四倍差异\MB60\F13TSHCCKW0004\annotation\KEGG\All-Unigene.fa.htm#gene28) | 353 | ko04146 |
| 29 | [Nucleotide excision repair](file:///E:\cao\MB%20转录组\MB60二四倍差异\MB60\F13TSHCCKW0004\annotation\KEGG\All-Unigene.fa.htm#gene29) | 339 | ko03420 |
| 30 | [Phagosome](file:///E:\cao\MB%20转录组\MB60二四倍差异\MB60\F13TSHCCKW0004\annotation\KEGG\All-Unigene.fa.htm#gene30) | 324 | ko04145 |
| 31 | [Pyruvate metabolism](file:///E:\cao\MB%20转录组\MB60二四倍差异\MB60\F13TSHCCKW0004\annotation\KEGG\All-Unigene.fa.htm#gene31) | 300 | ko00620 |
| 32 | [Flavonoid biosynthesis](file:///E:\cao\MB%20转录组\MB60二四倍差异\MB60\F13TSHCCKW0004\annotation\KEGG\All-Unigene.fa.htm#gene32) | 299 | ko00941 |
| 33 | [RNA polymerase](file:///E:\cao\MB%20转录组\MB60二四倍差异\MB60\F13TSHCCKW0004\annotation\KEGG\All-Unigene.fa.htm#gene33) | 297 | ko03020 |
| 34 | [N-Glycan biosynthesis](file:///E:\cao\MB%20转录组\MB60二四倍差异\MB60\F13TSHCCKW0004\annotation\KEGG\All-Unigene.fa.htm#gene34) | 294 | ko00510 |
| 35 | [Inositol phosphate metabolism](file:///E:\cao\MB%20转录组\MB60二四倍差异\MB60\F13TSHCCKW0004\annotation\KEGG\All-Unigene.fa.htm#gene35) | 289 | ko00562 |
| 36 | [Carotenoid biosynthesis](file:///E:\cao\MB%20转录组\MB60二四倍差异\MB60\F13TSHCCKW0004\annotation\KEGG\All-Unigene.fa.htm#gene36) | 289 | ko00906 |
| 37 | [Aminoacyl-tRNA biosynthesis](file:///E:\cao\MB%20转录组\MB60二四倍差异\MB60\F13TSHCCKW0004\annotation\KEGG\All-Unigene.fa.htm#gene37) | 289 | ko00970 |
| 38 | [Cyanoamino acid metabolism](file:///E:\cao\MB%20转录组\MB60二四倍差异\MB60\F13TSHCCKW0004\annotation\KEGG\All-Unigene.fa.htm#gene38) | 287 | ko00460 |
| 39 | [Basal transcription factors](file:///E:\cao\MB%20转录组\MB60二四倍差异\MB60\F13TSHCCKW0004\annotation\KEGG\All-Unigene.fa.htm#gene39) | 285 | ko03022 |
| 40 | [Other glycan degradation](file:///E:\cao\MB%20转录组\MB60二四倍差异\MB60\F13TSHCCKW0004\annotation\KEGG\All-Unigene.fa.htm#gene40) | 282 | ko00511 |
| 41 | [Zeatin biosynthesis](file:///E:\cao\MB%20转录组\MB60二四倍差异\MB60\F13TSHCCKW0004\annotation\KEGG\All-Unigene.fa.htm#gene41) | 271 | ko00908 |
| 42 | [Glycerolipid metabolism](file:///E:\cao\MB%20转录组\MB60二四倍差异\MB60\F13TSHCCKW0004\annotation\KEGG\All-Unigene.fa.htm#gene42) | 271 | ko00561 |
| 43 | [Terpenoid backbone biosynthesis](file:///E:\cao\MB%20转录组\MB60二四倍差异\MB60\F13TSHCCKW0004\annotation\KEGG\All-Unigene.fa.htm#gene43) | 266 | ko00900 |
| 44 | [Arginine and proline metabolism](file:///E:\cao\MB%20转录组\MB60二四倍差异\MB60\F13TSHCCKW0004\annotation\KEGG\All-Unigene.fa.htm#gene44) | 257 | ko00330 |
| 45 | [Ascorbate and aldarate metabolism](file:///E:\cao\MB%20转录组\MB60二四倍差异\MB60\F13TSHCCKW0004\annotation\KEGG\All-Unigene.fa.htm#gene45) | 254 | ko00053 |
| 46 | [Glycine, serine and threonine metabolism](file:///E:\cao\MB%20转录组\MB60二四倍差异\MB60\F13TSHCCKW0004\annotation\KEGG\All-Unigene.fa.htm#gene46) | 253 | ko00260 |
| 47 | [Cysteine and methionine metabolism](file:///E:\cao\MB%20转录组\MB60二四倍差异\MB60\F13TSHCCKW0004\annotation\KEGG\All-Unigene.fa.htm#gene47) | 251 | ko00270 |
| 48 | [Stilbenoid, diarylheptanoid and gingerol biosynthesis](file:///E:\cao\MB%20转录组\MB60二四倍差异\MB60\F13TSHCCKW0004\annotation\KEGG\All-Unigene.fa.htm#gene48) | 251 | ko00945 |
| 49 | [Glutathione metabolism](file:///E:\cao\MB%20转录组\MB60二四倍差异\MB60\F13TSHCCKW0004\annotation\KEGG\All-Unigene.fa.htm#gene49) | 234 | ko00480 |
| 50 | [Valine, leucine and isoleucine degradation](file:///E:\cao\MB%20转录组\MB60二四倍差异\MB60\F13TSHCCKW0004\annotation\KEGG\All-Unigene.fa.htm#gene50) | 233 | ko00280 |
| 51 | [Pentose phosphate pathway](file:///E:\cao\MB%20转录组\MB60二四倍差异\MB60\F13TSHCCKW0004\annotation\KEGG\All-Unigene.fa.htm#gene51) | 225 | ko00030 |
| 52 | [Glycosylphosphatidylinositol(GPI)-anchor biosynthesis](file:///E:\cao\MB%20转录组\MB60二四倍差异\MB60\F13TSHCCKW0004\annotation\KEGG\All-Unigene.fa.htm#gene52) | 225 | ko00563 |
| 53 | [Homologous recombination](file:///E:\cao\MB%20转录组\MB60二四倍差异\MB60\F13TSHCCKW0004\annotation\KEGG\All-Unigene.fa.htm#gene53) | 220 | ko03440 |
| 54 | [Porphyrin and chlorophyll metabolism](file:///E:\cao\MB%20转录组\MB60二四倍差异\MB60\F13TSHCCKW0004\annotation\KEGG\All-Unigene.fa.htm#gene54) | 218 | ko00860 |
| 55 | [Fructose and mannose metabolism](file:///E:\cao\MB%20转录组\MB60二四倍差异\MB60\F13TSHCCKW0004\annotation\KEGG\All-Unigene.fa.htm#gene55) | 218 | ko00051 |
| 56 | [Sphingolipid metabolism](file:///E:\cao\MB%20转录组\MB60二四倍差异\MB60\F13TSHCCKW0004\annotation\KEGG\All-Unigene.fa.htm#gene56) | 216 | ko00600 |
| 57 | [Regulation of autophagy](file:///E:\cao\MB%20转录组\MB60二四倍差异\MB60\F13TSHCCKW0004\annotation\KEGG\All-Unigene.fa.htm#gene57) | 214 | ko04140 |
| 58 | [Tyrosine metabolism](file:///E:\cao\MB%20转录组\MB60二四倍差异\MB60\F13TSHCCKW0004\annotation\KEGG\All-Unigene.fa.htm#gene58) | 214 | ko00350 |
| 59 | [Natural killer cell mediated cytotoxicity](file:///E:\cao\MB%20转录组\MB60二四倍差异\MB60\F13TSHCCKW0004\annotation\KEGG\All-Unigene.fa.htm#gene59) | 212 | ko04650 |
| 60 | [Carbon fixation in photosynthetic organisms](file:///E:\cao\MB%20转录组\MB60二四倍差异\MB60\F13TSHCCKW0004\annotation\KEGG\All-Unigene.fa.htm#gene60) | 207 | ko00710 |
| 61 | [Limonene and pinene degradation](file:///E:\cao\MB%20转录组\MB60二四倍差异\MB60\F13TSHCCKW0004\annotation\KEGG\All-Unigene.fa.htm#gene61) | 205 | ko00903 |
| 62 | [DNA replication](file:///E:\cao\MB%20转录组\MB60二四倍差异\MB60\F13TSHCCKW0004\annotation\KEGG\All-Unigene.fa.htm#gene62) | 199 | ko03030 |
| 63 | Phenylalanine metabolism | 197 | ko00360 |
| 64 | Base excision repair | 190 | ko03410 |
| 65 | [Mismatch repair](file:///E:\cao\MB%20转录组\MB60二四倍差异\MB60\F13TSHCCKW0004\annotation\KEGG\All-Unigene.fa.htm#gene65) | 188 | ko03430 |
| 66 | [Fatty acid metabolism](file:///E:\cao\MB%20转录组\MB60二四倍差异\MB60\F13TSHCCKW0004\annotation\KEGG\All-Unigene.fa.htm#gene66) | 188 | ko00071 |
| 67 | [Glycosaminoglycan degradation](file:///E:\cao\MB%20转录组\MB60二四倍差异\MB60\F13TSHCCKW0004\annotation\KEGG\All-Unigene.fa.htm#gene67) | 176 | ko00531 |
| 68 | [Photosynthesis](file:///E:\cao\MB%20转录组\MB60二四倍差异\MB60\F13TSHCCKW0004\annotation\KEGG\All-Unigene.fa.htm#gene68) | 175 | ko00195 |
| 69 | [Alanine, aspartate and glutamate metabolism](file:///E:\cao\MB%20转录组\MB60二四倍差异\MB60\F13TSHCCKW0004\annotation\KEGG\All-Unigene.fa.htm#gene69) | 170 | ko00250 |
| 70 | [Propanoate metabolism](file:///E:\cao\MB%20转录组\MB60二四倍差异\MB60\F13TSHCCKW0004\annotation\KEGG\All-Unigene.fa.htm#gene70) | 168 | ko00640 |
| 71 | [Protein export](file:///E:\cao\MB%20转录组\MB60二四倍差异\MB60\F13TSHCCKW0004\annotation\KEGG\All-Unigene.fa.htm#gene71) | 167 | ko03060 |
| 72 | [Phenylalanine, tyrosine and tryptophan biosynthesis](file:///E:\cao\MB%20转录组\MB60二四倍差异\MB60\F13TSHCCKW0004\annotation\KEGG\All-Unigene.fa.htm#gene72) | 167 | ko00400 |
| 73 | [Proteasome](file:///E:\cao\MB%20转录组\MB60二四倍差异\MB60\F13TSHCCKW0004\annotation\KEGG\All-Unigene.fa.htm#gene73) | 167 | ko03050 |
| 74 | [Nitrogen metabolism](file:///E:\cao\MB%20转录组\MB60二四倍差异\MB60\F13TSHCCKW0004\annotation\KEGG\All-Unigene.fa.htm#gene74) | 163 | ko00910 |
| 75 | [Tryptophan metabolism](file:///E:\cao\MB%20转录组\MB60二四倍差异\MB60\F13TSHCCKW0004\annotation\KEGG\All-Unigene.fa.htm#gene75) | 161 | ko00380 |
| 76 | [Glyoxylate and dicarboxylate metabolism](file:///E:\cao\MB%20转录组\MB60二四倍差异\MB60\F13TSHCCKW0004\annotation\KEGG\All-Unigene.fa.htm#gene76) | 160 | ko00630 |
| 77 | [Steroid biosynthesis](file:///E:\cao\MB%20转录组\MB60二四倍差异\MB60\F13TSHCCKW0004\annotation\KEGG\All-Unigene.fa.htm#gene77) | 154 | ko00100 |
| 78 | [beta-Alanine metabolism](file:///E:\cao\MB%20转录组\MB60二四倍差异\MB60\F13TSHCCKW0004\annotation\KEGG\All-Unigene.fa.htm#gene78) | 150 | ko00410 |
| 79 | [alpha-Linolenic acid metabolism](file:///E:\cao\MB%20转录组\MB60二四倍差异\MB60\F13TSHCCKW0004\annotation\KEGG\All-Unigene.fa.htm#gene79) | 144 | ko00592 |
| 80 | [Pantothenate and CoA biosynthesis](file:///E:\cao\MB%20转录组\MB60二四倍差异\MB60\F13TSHCCKW0004\annotation\KEGG\All-Unigene.fa.htm#gene80) | 141 | ko00770 |
| 81 | [SNARE interactions in vesicular transport](file:///E:\cao\MB%20转录组\MB60二四倍差异\MB60\F13TSHCCKW0004\annotation\KEGG\All-Unigene.fa.htm#gene81) | 138 | ko04130 |
| 82 | [Citrate cycle (TCA cycle)](file:///E:\cao\MB%20转录组\MB60二四倍差异\MB60\F13TSHCCKW0004\annotation\KEGG\All-Unigene.fa.htm#gene82) | 137 | ko00020 |
| 83 | [Flavone and flavonol biosynthesis](file:///E:\cao\MB%20转录组\MB60二四倍差异\MB60\F13TSHCCKW0004\annotation\KEGG\All-Unigene.fa.htm#gene83) | 128 | ko00944 |
| 84 | [Cutin, suberine and wax biosynthesis](file:///E:\cao\MB%20转录组\MB60二四倍差异\MB60\F13TSHCCKW0004\annotation\KEGG\All-Unigene.fa.htm#gene84) | 125 | ko00073 |
| 85 | [Lysine degradation](file:///E:\cao\MB%20转录组\MB60二四倍差异\MB60\F13TSHCCKW0004\annotation\KEGG\All-Unigene.fa.htm#gene85) | 123 | ko00310 |
| 86 | [Butanoate metabolism](file:///E:\cao\MB%20转录组\MB60二四倍差异\MB60\F13TSHCCKW0004\annotation\KEGG\All-Unigene.fa.htm#gene86) | 121 | ko00650 |
| 87 | [Ubiquinone and other terpenoid-quinone biosynthesis](file:///E:\cao\MB%20转录组\MB60二四倍差异\MB60\F13TSHCCKW0004\annotation\KEGG\All-Unigene.fa.htm#gene87) | 114 | ko00130 |
| 88 | [Fatty acid biosynthesis](file:///E:\cao\MB%20转录组\MB60二四倍差异\MB60\F13TSHCCKW0004\annotation\KEGG\All-Unigene.fa.htm#gene88) | 113 | ko00061 |
| 89 | [Diterpenoid biosynthesis](file:///E:\cao\MB%20转录组\MB60二四倍差异\MB60\F13TSHCCKW0004\annotation\KEGG\All-Unigene.fa.htm#gene89) | 113 | ko00904 |
| 90 | [Glycosphingolipid biosynthesis - ganglio series](file:///E:\cao\MB%20转录组\MB60二四倍差异\MB60\F13TSHCCKW0004\annotation\KEGG\All-Unigene.fa.htm#gene90) | 107 | ko00604 |
| 91 | [Isoquinoline alkaloid biosynthesis](file:///E:\cao\MB%20转录组\MB60二四倍差异\MB60\F13TSHCCKW0004\annotation\KEGG\All-Unigene.fa.htm#gene91) | 104 | ko00950 |
| 92 | [Lysine biosynthesis](file:///E:\cao\MB%20转录组\MB60二四倍差异\MB60\F13TSHCCKW0004\annotation\KEGG\All-Unigene.fa.htm#gene92) | 100 | ko00300 |
| 93 | [Histidine metabolism](file:///E:\cao\MB%20转录组\MB60二四倍差异\MB60\F13TSHCCKW0004\annotation\KEGG\All-Unigene.fa.htm#gene93) | 99 | ko00340 |
| 94 | [Riboflavin metabolism](file:///E:\cao\MB%20转录组\MB60二四倍差异\MB60\F13TSHCCKW0004\annotation\KEGG\All-Unigene.fa.htm#gene94) | 99 | ko00740 |
| 95 | [Biosynthesis of unsaturated fatty acids](file:///E:\cao\MB%20转录组\MB60二四倍差异\MB60\F13TSHCCKW0004\annotation\KEGG\All-Unigene.fa.htm#gene95) | 94 | ko01040 |
| 96 | [Brassinosteroid biosynthesis](file:///E:\cao\MB%20转录组\MB60二四倍差异\MB60\F13TSHCCKW0004\annotation\KEGG\All-Unigene.fa.htm#gene96) | 90 | ko00905 |
| 97 | [Sulfur metabolism](file:///E:\cao\MB%20转录组\MB60二四倍差异\MB60\F13TSHCCKW0004\annotation\KEGG\All-Unigene.fa.htm#gene97) | 90 | ko00920 |
| 98 | [One carbon pool by folate](file:///E:\cao\MB%20转录组\MB60二四倍差异\MB60\F13TSHCCKW0004\annotation\KEGG\All-Unigene.fa.htm#gene98) | 90 | ko00670 |
| 99 | [Folate biosynthesis](file:///E:\cao\MB%20转录组\MB60二四倍差异\MB60\F13TSHCCKW0004\annotation\KEGG\All-Unigene.fa.htm#gene99) | 88 | ko00790 |
| 100 | [Valine, leucine and isoleucine biosynthesis](file:///E:\cao\MB%20转录组\MB60二四倍差异\MB60\F13TSHCCKW0004\annotation\KEGG\All-Unigene.fa.htm#gene100) | 87 | ko00290 |
| 101 | [Fatty acid elongation](file:///E:\cao\MB%20转录组\MB60二四倍差异\MB60\F13TSHCCKW0004\annotation\KEGG\All-Unigene.fa.htm#gene101) | 84 | ko00062 |
| 102 | [Linoleic acid metabolism](file:///E:\cao\MB%20转录组\MB60二四倍差异\MB60\F13TSHCCKW0004\annotation\KEGG\All-Unigene.fa.htm#gene102) | 81 | ko00591 |
| 103 | [Circadian rhythm - mammal](file:///E:\cao\MB%20转录组\MB60二四倍差异\MB60\F13TSHCCKW0004\annotation\KEGG\All-Unigene.fa.htm#gene103) | 81 | ko04710 |
| 104 | [Tropane, piperidine and pyridine alkaloid biosynthesis](file:///E:\cao\MB%20转录组\MB60二四倍差异\MB60\F13TSHCCKW0004\annotation\KEGG\All-Unigene.fa.htm#gene104) | 79 | ko00960 |
| 105 | [Benzoxazinoid biosynthesis](file:///E:\cao\MB%20转录组\MB60二四倍差异\MB60\F13TSHCCKW0004\annotation\KEGG\All-Unigene.fa.htm#gene105) | 72 | ko00402 |
| 106 | [Taurine and hypotaurine metabolism](file:///E:\cao\MB%20转录组\MB60二四倍差异\MB60\F13TSHCCKW0004\annotation\KEGG\All-Unigene.fa.htm#gene106) | 66 | ko00430 |
| 107 | [Photosynthesis - antenna proteins](file:///E:\cao\MB%20转录组\MB60二四倍差异\MB60\F13TSHCCKW0004\annotation\KEGG\All-Unigene.fa.htm#gene107) | 66 | ko00196 |
| 108 | [Glucosinolate biosynthesis](file:///E:\cao\MB%20转录组\MB60二四倍差异\MB60\F13TSHCCKW0004\annotation\KEGG\All-Unigene.fa.htm#gene108) | 65 | ko00966 |
| 109 | [Isoflavonoid biosynthesis](file:///E:\cao\MB%20转录组\MB60二四倍差异\MB60\F13TSHCCKW0004\annotation\KEGG\All-Unigene.fa.htm#gene109) | 64 | ko00943 |
| 110 | [Selenocompound metabolism](file:///E:\cao\MB%20转录组\MB60二四倍差异\MB60\F13TSHCCKW0004\annotation\KEGG\All-Unigene.fa.htm#gene110) | 63 | ko00450 |
| 111 | [Arachidonic acid metabolism](file:///E:\cao\MB%20转录组\MB60二四倍差异\MB60\F13TSHCCKW0004\annotation\KEGG\All-Unigene.fa.htm#gene111) | 63 | ko00590 |
| 112 | [Vitamin B6 metabolism](file:///E:\cao\MB%20转录组\MB60二四倍差异\MB60\F13TSHCCKW0004\annotation\KEGG\All-Unigene.fa.htm#gene112) | 60 | ko00750 |
| 113 | [Nicotinate and nicotinamide metabolism](file:///E:\cao\MB%20转录组\MB60二四倍差异\MB60\F13TSHCCKW0004\annotation\KEGG\All-Unigene.fa.htm#gene113) | 58 | ko00760 |
| 114 | [Monoterpenoid biosynthesis](file:///E:\cao\MB%20转录组\MB60二四倍差异\MB60\F13TSHCCKW0004\annotation\KEGG\All-Unigene.fa.htm#gene114) | 56 | ko00902 |
| 115 | [Sesquiterpenoid and triterpenoid biosynthesis](file:///E:\cao\MB%20转录组\MB60二四倍差异\MB60\F13TSHCCKW0004\annotation\KEGG\All-Unigene.fa.htm#gene115) | 50 | ko00909 |
| 116 | [Non-homologous end-joining](file:///E:\cao\MB%20转录组\MB60二四倍差异\MB60\F13TSHCCKW0004\annotation\KEGG\All-Unigene.fa.htm#gene116) | 42 | ko03450 |
| 117 | [Sulfur relay system](file:///E:\cao\MB%20转录组\MB60二四倍差异\MB60\F13TSHCCKW0004\annotation\KEGG\All-Unigene.fa.htm#gene117) | 41 | ko04122 |
| 118 | [Glycosphingolipid biosynthesis - globo series](file:///E:\cao\MB%20转录组\MB60二四倍差异\MB60\F13TSHCCKW0004\annotation\KEGG\All-Unigene.fa.htm#gene118) | 40 | ko00603 |
| 119 | [Thiamine metabolism](file:///E:\cao\MB%20转录组\MB60二四倍差异\MB60\F13TSHCCKW0004\annotation\KEGG\All-Unigene.fa.htm#gene119) | 39 | ko00730 |
| 120 | [Indole alkaloid biosynthesis](file:///E:\cao\MB%20转录组\MB60二四倍差异\MB60\F13TSHCCKW0004\annotation\KEGG\All-Unigene.fa.htm#gene120) | 35 | ko00901 |
| 121 | [Biotin metabolism](file:///E:\cao\MB%20转录组\MB60二四倍差异\MB60\F13TSHCCKW0004\annotation\KEGG\All-Unigene.fa.htm#gene121) | 32 | ko00780 |
| 122 | Other types of O-glycan biosynthesis | 31 | ko00514 |
| 123 | Synthesis and degradation of ketone bodies | 30 | ko00072 |
| 124 | [C5-Branched dibasic acid metabolism](file:///E:\cao\MB%20转录组\MB60二四倍差异\MB60\F13TSHCCKW0004\annotation\KEGG\All-Unigene.fa.htm#gene124) | 27 | ko00660 |
| 125 | Lipoic acid metabolism | 16 | ko00785 |
| 126 | Anthocyanin biosynthesis | 7 | ko00942 |
| 127 | Caffeine metabolism | 3 | ko00232 |
| 128 | Betalain biosynthesis | 2 | ko00965 |

*: the number of the all-unigenes involved in the corresponding pathway**.**
