# Supplementary material for: Transcriptome, microRNA, and degradome analyses of the gene expression of Paulownia with phytoplamsa
Source: BMC Genomics. 2015 Nov 4;16:896. doi: 10.1186/s12864-015-2074-3 (PMC4634154; doi:10.1186/s12864-015-2074-3)
Supplement: Additional file 11: Table S11. — Length distribution of P. tomentosa small RNAs obtained by high-throughput sequencing in HP libraries a: Nucleotide bias at A position of sRNA tags; b: Nucleotide bias at U position of sRNA tags; c: Nucleotide bias at C position of sRNA tags; d: Nucleotide bias at G position of sRNA tags. (DOCX 29.9 kb) [file 12864_2015_2074_MOESM11_ESM.docx]

**Additional file 11: Table S11 Length distribution of *P. tomentosa* small RNAs obtained by high-throughput sequencing in HP libraries**

| sRNA size(nt) | A^a^ | |  | U^b^ | |  | C^c^ | |  | G^d^ | |
| --- | --- | --- | --- | --- | --- | --- | --- | --- | --- | --- | --- |
|  | Number | Percentage (%) |  | Number | Percentage (%) |  | Number | Percentage (%) |  | Number | Percentage (%) |
| 18 | 939 | 12.84 |  | 5956 | 81.44 |  | 184 | 2.52 |  | 234 | 3.20 |
| 19 | 0 | 0.00 |  | 19098 | 99.83 |  | 1 | 0.01 |  | 31 | 0.16 |
| 20 | 92 | 0.02 |  | 437559 | 99.58 |  | 1657 | 0.38 |  | 115 | 0.03 |
| 21 | 87514 | 0.16 |  | 53939611 | 99.77 |  | 20080 | 0.04 |  | 16221 | 0.03 |
| 22 | 0 | 0.00 |  | 27769 | 95.71 |  | 0 | 0.00 |  | 1246 | 4.29 |
| 23 | 0 | 0.00 |  | 23 | 100.00 |  | 0 | 0.00 |  | 0 | 0.00 |
| 24 | 0 | 0.00 |  | 0 | 0.00 |  | 0 | 0.00 |  | 0 | 0.00 |

a: Nucleotide bias at A position of sRNA tags; b: Nucleotide bias at U position of sRNA tags; c: Nucleotide bias at C position of sRNA tags; d: Nucleotide bias at G position of sRNA tags.
