# Supplementary material for: Transcriptome, microRNA, and degradome analyses of the gene expression of Paulownia with phytoplamsa
Source: BMC Genomics. 2015 Nov 4;16:896. doi: 10.1186/s12864-015-2074-3 (PMC4634154; doi:10.1186/s12864-015-2074-3)
Supplement: Additional file 12: Table S12. — Length distribution of P. tomentosa small RNAs obtained by high-throughput sequencing in PIP libraries. (DOCX 30.5 kb) [file 12864_2015_2074_MOESM12_ESM.docx]

**Additional file 12: Table S12 Length distribution of *P. tomentosa* small RNAs obtained by high-throughput sequencing in PIP libraries**

| sRNA size(nt) | A^a^ | |  | U^b^ | |  | C^c^ | |  | G^d^ | |
| --- | --- | --- | --- | --- | --- | --- | --- | --- | --- | --- | --- |
|  | Number | Percentage (%) |  | Number | Percentage (%) |  | Number | Percentage (%) |  | Number | Percentage (%) |
| 18 | 736 | 12.51 |  | 3558 | 60.49 |  | 1107 | 18.82 |  | 481 | 8.18 |
| 19 | 0 | 0.00 |  | 37644 | 99.77 |  | 8 | 0.02 |  | 79 | 0.21 |
| 20 | 89 | 0.01 |  | 885538 | 99.80 |  | 1383 | 0.16 |  | 296 | 0.03 |
| 21 | 21860 | 0.03 |  | 81939073 | 99.90 |  | 34010 | 0.04 |  | 26322 | 0.03 |
| 22 | 1 | 0.00 |  | 36736 | 91.03 |  | 1 | 0.00 |  | 3620 | 8.97 |
| 23 | 3 | 0.45 |  | 0 | 0.00 |  | 0 | 0.00 |  | 663 | 99.55 |
| 24 | 0 | 0.00 |  | 0 | 0.00 |  | 0 | 0.00 |  | 0 | 0.00 |
| 25 | 5 | 100.00 |  | 0 | 0.00 |  | 0 | 0.00 |  | 0 | 0.00 |

a: Nucleotide bias at A position of sRNA tags; b: Nucleotide bias at U position of sRNA tags; c: Nucleotide bias at C position of sRNA tags; d: Nucleotide bias at G position of sRNA tags.
