# Supplementary material for: Transcriptome, microRNA, and degradome analyses of the gene expression of Paulownia with phytoplamsa
Source: BMC Genomics. 2015 Nov 4;16:896. doi: 10.1186/s12864-015-2074-3 (PMC4634154; doi:10.1186/s12864-015-2074-3)
Supplement: Additional file 13: Table S13. — Length distribution of P. tomentosa small RNAs obtained by high-throughput sequencing in PIP-60 libraries. (DOCX 31.3 kb) [file 12864_2015_2074_MOESM13_ESM.docx]

**Additional file 13: Table S13 Length distribution of *P. tomentosa* small RNAs obtained by high-throughput sequencing in PIP-60 libraries**

| sRNA size(nt) | A^a^ | |  | U^b^ | |  | C^c^ | |  | G^d^ | |
| --- | --- | --- | --- | --- | --- | --- | --- | --- | --- | --- | --- |
|  | Number | Percentage (%) |  | Number | Percentage (%) |  | Number | Percentage (%) |  | Number | Percentage (%) |
| 18 | 684 | 17.74 |  | 2752 | 71.37 |  | 31 | 0.80 |  | 389 | 10.09 |
| 19 | 0 | 0.00 |  | 29976 | 99.55 |  | 76 | 0.25 |  | 61 | 0.20 |
| 20 | 94 | 0.01 |  | 849651 | 99.83 |  | 1173 | 0.14 |  | 203 | 0.02 |
| 21 | 27241 | 0.04 |  | 75050743 | 99.89 |  | 22576 | 0.03 |  | 30458 | 0.04 |
| 22 | 0 | 0.00 |  | 41057 | 94.37 |  | 1 | 0.00 |  | 2450 | 5.63 |
| 23 | 1 | 0.10 |  | 0 | 0.00 |  | 1 | 0.10 |  | 986 | 99.80 |
| 25 | 19 | 100.00 |  | 0 | 0.00 |  | 0 | 0.00 |  | 0 | 0.00 |
| 26 | 27 | 100.00 |  | 0 | 0.00 |  | 0 | 0.00 |  | 0 | 0.00 |
| 24 | 0 | 0.00 |  | 0 | 0.00 |  | 0 | 0.00 |  | 0 | 0.00 |

a: Nucleotide bias at A position of sRNA tags; b: Nucleotide bias at U position of sRNA tags; c: Nucleotide bias at C position of sRNA tags; d: Nucleotide bias at G position of sRNA tags.
