# Supplementary material for: Transcriptome, microRNA, and degradome analyses of the gene expression of Paulownia with phytoplamsa
Source: BMC Genomics. 2015 Nov 4;16:896. doi: 10.1186/s12864-015-2074-3 (PMC4634154; doi:10.1186/s12864-015-2074-3)
Supplement: Additional file 19: Table S19. — KEGG pathway analysis of the P. tomentosa DEGs a: the number of the differentially expressed unigenes that involved the corresponding pathway; b: the number of the all-unigene that involved the corresponding pathway; c: indicating a significantly enriched GO terms among the differentially expressed unigenes; d: indicating a significantly enriched pathway among the differentially expressed unigenes. (DOCX 34.0 kb) [file 12864_2015_2074_MOESM19_ESM.docx]

**Additional file 19: Table S19 KEGG pathway analysis of the *P. tomentosa* DEGs**

| # | Pathway | DEG count^a^ | All-Unigene Count ^b^ | Pvalue^c^ | Qvalue^d^ | Pathway ID |
| --- | --- | --- | --- | --- | --- | --- |
| 1 | [Stilbenoid, diarylheptanoid and gingerol biosynthesis](file:///E:\cao\MB%20转录组\最新M2_MB_MB60\result_M2-vs-MB_MB-vs-MB60_2\pathway\hebing_id.htm#gene1) | 27 | 251 | 5.74963e-18 | 5.289660e-16 | ko00945 |
| 2 | [Biosynthesis of secondary metabolites](file:///E:\cao\MB%20转录组\最新M2_MB_MB60\result_M2-vs-MB_MB-vs-MB60_2\pathway\hebing_id.htm#gene2) | 90 | 4041 | 3.56541e-09 | 1.341950e-07 | ko01110 |
| 3 | [Flavonoid biosynthesis](file:///E:\cao\MB%20转录组\最新M2_MB_MB60\result_M2-vs-MB_MB-vs-MB60_2\pathway\hebing_id.htm#gene3) | 19 | 299 | 4.375923e-09 | 1.341950e-07 | ko00941 |
| 4 | [Limonene and pinene degradation](file:///E:\cao\MB%20转录组\最新M2_MB_MB60\result_M2-vs-MB_MB-vs-MB60_2\pathway\hebing_id.htm#gene4) | 14 | 205 | 1.986472e-07 | 4.568886e-06 | ko00903 |
| 5 | [Cutin, suberine and wax biosynthesis](file:///E:\cao\MB%20转录组\最新M2_MB_MB60\result_M2-vs-MB_MB-vs-MB60_2\pathway\hebing_id.htm#gene5) | 10 | 125 | 2.6874e-06 | 4.944816e-05 | ko00073 |
| 6 | [Fatty acid biosynthesis](file:///E:\cao\MB%20转录组\最新M2_MB_MB60\result_M2-vs-MB_MB-vs-MB60_2\pathway\hebing_id.htm#gene6) | 9 | 113 | 8.799006e-06 | 1.349181e-04 | ko00061 |
| 7 | [Isoquinoline alkaloid biosynthesis](file:///E:\cao\MB%20转录组\最新M2_MB_MB60\result_M2-vs-MB_MB-vs-MB60_2\pathway\hebing_id.htm#gene7) | 8 | 104 | 3.573527e-05 | 4.696635e-04 | ko00950 |
| 8 | [DNA replication](file:///E:\cao\MB%20转录组\最新M2_MB_MB60\result_M2-vs-MB_MB-vs-MB60_2\pathway\hebing_id.htm#gene8) | 10 | 199 | 0.0001485199 | 1.707979e-03 | ko03030 |
| 9 | [ABC transporters](file:///E:\cao\MB%20转录组\最新M2_MB_MB60\result_M2-vs-MB_MB-vs-MB60_2\pathway\hebing_id.htm#gene9) | 17 | 514 | 0.0001699809 | 1.737583e-03 | ko02010 |
| 10 | [Benzoxazinoid biosynthesis](file:///E:\cao\MB%20转录组\最新M2_MB_MB60\result_M2-vs-MB_MB-vs-MB60_2\pathway\hebing_id.htm#gene10) | 6 | 72 | 0.0002202395 | 2.026203e-03 | ko00402 |
| 11 | [Metabolic pathways](file:///E:\cao\MB%20转录组\最新M2_MB_MB60\result_M2-vs-MB_MB-vs-MB60_2\pathway\hebing_id.htm#gene11) | 132 | 8420 | 0.0002618055 | 2.189646e-03 | ko01100 |
| 12 | [Phenylpropanoid biosynthesis](file:///E:\cao\MB%20转录组\最新M2_MB_MB60\result_M2-vs-MB_MB-vs-MB60_2\pathway\hebing_id.htm#gene12) | 17 | 574 | 0.0005995565 | 4.596600e-03 | ko00940 |
| 13 | [Flavone and flavonol biosynthesis](file:///E:\cao\MB%20转录组\最新M2_MB_MB60\result_M2-vs-MB_MB-vs-MB60_2\pathway\hebing_id.htm#gene13) | 7 | 128 | 0.00088288 | 6.248074e-03 | ko00944 |
| 14 | [Isoflavonoid biosynthesis](file:///E:\cao\MB%20转录组\最新M2_MB_MB60\result_M2-vs-MB_MB-vs-MB60_2\pathway\hebing_id.htm#gene14) | 5 | 64 | 0.0009963102 | 6.296603e-03 | ko00943 |
| 15 | [Pyruvate metabolism](file:///E:\cao\MB%20转录组\最新M2_MB_MB60\result_M2-vs-MB_MB-vs-MB60_2\pathway\hebing_id.htm#gene15) | 11 | 300 | 0.001026620 | 6.296603e-03 | ko00620 |
| 16 | [Propanoate metabolism](file:///E:\cao\MB%20转录组\最新M2_MB_MB60\result_M2-vs-MB_MB-vs-MB60_2\pathway\hebing_id.htm#gene16) | 7 | 168 | 0.004124266 | 2.326736e-02 | ko00640 |
| 17 | [Tyrosine metabolism](file:///E:\cao\MB%20转录组\最新M2_MB_MB60\result_M2-vs-MB_MB-vs-MB60_2\pathway\hebing_id.htm#gene17) | 8 | 214 | 0.004299404 | 2.326736e-02 | ko00350 |
| 18 | [Glycerophospholipid metabolism](file:///E:\cao\MB%20转录组\最新M2_MB_MB60\result_M2-vs-MB_MB-vs-MB60_2\pathway\hebing_id.htm#gene18) | 23 | 1139 | 0.0102795 | 5.253967e-02 | ko00564 |
| 19 | [Endocytosis](file:///E:\cao\MB%20转录组\最新M2_MB_MB60\result_M2-vs-MB_MB-vs-MB60_2\pathway\hebing_id.htm#gene19) | 22 | 1107 | 0.01407154 | 6.813588e-02 | ko04144 |
| 20 | [Ether lipid metabolism](file:///E:\cao\MB%20转录组\最新M2_MB_MB60\result_M2-vs-MB_MB-vs-MB60_2\pathway\hebing_id.htm#gene20) | 18 | 859 | 0.01553491 | 7.146059e-02 | ko00565 |
| 21 | [Other glycan degradation](file:///E:\cao\MB%20转录组\最新M2_MB_MB60\result_M2-vs-MB_MB-vs-MB60_2\pathway\hebing_id.htm#gene21) | 8 | 282 | 0.02033346 | 8.907992e-02 | ko00511 |
| 22 | [Ubiquitin mediated proteolysis](file:///E:\cao\MB%20转录组\最新M2_MB_MB60\result_M2-vs-MB_MB-vs-MB60_2\pathway\hebing_id.htm#gene22) | 16 | 789 | 0.02820504 | 1.179483e-01 | ko04120 |
| 23 | [Glucosinolate biosynthesis](file:///E:\cao\MB%20转录组\最新M2_MB_MB60\result_M2-vs-MB_MB-vs-MB60_2\pathway\hebing_id.htm#gene23) | 3 | 65 | 0.0424469 | 1.681764e-01 | ko00966 |
| 24 | [Tryptophan metabolism](file:///E:\cao\MB%20转录组\最新M2_MB_MB60\result_M2-vs-MB_MB-vs-MB60_2\pathway\hebing_id.htm#gene24) | 5 | 161 | 0.0438721 | 1.681764e-01 | ko00380 |
| 25 | [Carotenoid biosynthesis](file:///E:\cao\MB%20转录组\最新M2_MB_MB60\result_M2-vs-MB_MB-vs-MB60_2\pathway\hebing_id.htm#gene25) | 7 | 289 | 0.05865719 | 2.039430e-01 | ko00906 |
| 26 | [Photosynthesis](file:///E:\cao\MB%20转录组\最新M2_MB_MB60\result_M2-vs-MB_MB-vs-MB60_2\pathway\hebing_id.htm#gene26) | 5 | 175 | 0.05869696 | 2.039430e-01 | ko00195 |
| 27 | [Glycosaminoglycan degradation](file:///E:\cao\MB%20转录组\最新M2_MB_MB60\result_M2-vs-MB_MB-vs-MB60_2\pathway\hebing_id.htm#gene27) | 5 | 176 | 0.05985284 | 2.039430e-01 | ko00531 |
| 28 | [Plant-pathogen interaction](file:///E:\cao\MB%20转录组\最新M2_MB_MB60\result_M2-vs-MB_MB-vs-MB60_2\pathway\hebing_id.htm#gene28) | 34 | 2212 | 0.07677009 | 2.522446e-01 | ko04626 |
| 29 | [Anthocyanin biosynthesis](file:///E:\cao\MB%20转录组\最新M2_MB_MB60\result_M2-vs-MB_MB-vs-MB60_2\pathway\hebing_id.htm#gene29) | 1 | 7 | 0.08033017 | 2.548405e-01 | ko00942 |
| 30 | [alpha-Linolenic acid metabolism](file:///E:\cao\MB%20转录组\最新M2_MB_MB60\result_M2-vs-MB_MB-vs-MB60_2\pathway\hebing_id.htm#gene30) | 4 | 144 | 0.09349626 | 2.867219e-01 | ko00592 |
| 31 | [Cyanoamino acid metabolism](file:///E:\cao\MB%20转录组\最新M2_MB_MB60\result_M2-vs-MB_MB-vs-MB60_2\pathway\hebing_id.htm#gene31) | 6 | 287 | 0.1290817 | 3.830812e-01 | ko00460 |
| 32 | [N-Glycan biosynthesis](file:///E:\cao\MB%20转录组\最新M2_MB_MB60\result_M2-vs-MB_MB-vs-MB60_2\pathway\hebing_id.htm#gene32) | 6 | 294 | 0.1398765 | 4.021449e-01 | ko00510 |
| 33 | [Cysteine and methionine metabolism](file:///E:\cao\MB%20转录组\最新M2_MB_MB60\result_M2-vs-MB_MB-vs-MB60_2\pathway\hebing_id.htm#gene33) | 5 | 251 | 0.1805622 | 5.033855e-01 | ko00270 |
| 34 | [Ascorbate and aldarate metabolism](file:///E:\cao\MB%20转录组\最新M2_MB_MB60\result_M2-vs-MB_MB-vs-MB60_2\pathway\hebing_id.htm#gene34) | 5 | 254 | 0.1865913 | 5.048941e-01 | ko00053 |
| 35 | [Pentose and glucuronate interconversions](file:///E:\cao\MB%20转录组\最新M2_MB_MB60\result_M2-vs-MB_MB-vs-MB60_2\pathway\hebing_id.htm#gene35) | 7 | 419 | 0.2334952 | 6.067287e-01 | ko00040 |
| 36 | [Starch and sucrose metabolism](file:///E:\cao\MB%20转录组\最新M2_MB_MB60\result_M2-vs-MB_MB-vs-MB60_2\pathway\hebing_id.htm#gene36) | 14 | 959 | 0.2543414 | 6.067287e-01 | ko00500 |
| 37 | [Phosphatidylinositol signaling system](file:///E:\cao\MB%20转录组\最新M2_MB_MB60\result_M2-vs-MB_MB-vs-MB60_2\pathway\hebing_id.htm#gene37) | 6 | 361 | 0.2608214 | 6.067287e-01 | ko04070 |
| 38 | [Porphyrin and chlorophyll metabolism](file:///E:\cao\MB%20转录组\最新M2_MB_MB60\result_M2-vs-MB_MB-vs-MB60_2\pathway\hebing_id.htm#gene38) | 4 | 218 | 0.2614998 | 6.067287e-01 | ko00860 |
| 39 | [Inositol phosphate metabolism](file:///E:\cao\MB%20转录组\最新M2_MB_MB60\result_M2-vs-MB_MB-vs-MB60_2\pathway\hebing_id.htm#gene39) | 5 | 289 | 0.2615606 | 6.067287e-01 | ko00562 |
| 40 | [Fatty acid elongation](file:///E:\cao\MB%20转录组\最新M2_MB_MB60\result_M2-vs-MB_MB-vs-MB60_2\pathway\hebing_id.htm#gene40) | 2 | 84 | 0.2637951 | 6.067287e-01 | ko00062 |
| 41 | [Glycosylphosphatidylinositol(GPI)-anchor biosynthesis](file:///E:\cao\MB%20转录组\最新M2_MB_MB60\result_M2-vs-MB_MB-vs-MB60_2\pathway\hebing_id.htm#gene41) | 4 | 225 | 0.2798434 | 6.209121e-01 | ko00563 |
| 42 | [Plant hormone signal transduction](file:///E:\cao\MB%20转录组\最新M2_MB_MB60\result_M2-vs-MB_MB-vs-MB60_2\pathway\hebing_id.htm#gene42) | 27 | 2012 | 0.2857328 | 6.209121e-01 | ko04075 |
| 43 | [Sulfur metabolism](file:///E:\cao\MB%20转录组\最新M2_MB_MB60\result_M2-vs-MB_MB-vs-MB60_2\pathway\hebing_id.htm#gene43) | 2 | 90 | 0.2902089 | 6.209121e-01 | ko00920 |
| 44 | [Biotin metabolism](file:///E:\cao\MB%20转录组\最新M2_MB_MB60\result_M2-vs-MB_MB-vs-MB60_2\pathway\hebing_id.htm#gene44) | 1 | 32 | 0.3181463 | 6.612770e-01 | ko00780 |
| 45 | [Riboflavin metabolism](file:///E:\cao\MB%20转录组\最新M2_MB_MB60\result_M2-vs-MB_MB-vs-MB60_2\pathway\hebing_id.htm#gene45) | 2 | 99 | 0.3295705 | 6.612770e-01 | ko00740 |
| 46 | [Ribosome](file:///E:\cao\MB%20转录组\最新M2_MB_MB60\result_M2-vs-MB_MB-vs-MB60_2\pathway\hebing_id.htm#gene46) | 10 | 705 | 0.3306385 | 6.612770e-01 | ko03010 |
| 47 | [Amino sugar and nucleotide sugar metabolism](file:///E:\cao\MB%20转录组\最新M2_MB_MB60\result_M2-vs-MB_MB-vs-MB60_2\pathway\hebing_id.htm#gene47) | 6 | 404 | 0.3492628 | 6.836634e-01 | ko00520 |
| 48 | [Arginine and proline metabolism](file:///E:\cao\MB%20转录组\最新M2_MB_MB60\result_M2-vs-MB_MB-vs-MB60_2\pathway\hebing_id.htm#gene48) | 4 | 257 | 0.3653991 | 7.003483e-01 | ko00330 |
| 49 | [Diterpenoid biosynthesis](file:///E:\cao\MB%20转录组\最新M2_MB_MB60\result_M2-vs-MB_MB-vs-MB60_2\pathway\hebing_id.htm#gene49) | 2 | 113 | 0.3894548 | 7.312213e-01 | ko00904 |
| 50 | [Pyrimidine metabolism](file:///E:\cao\MB%20转录组\最新M2_MB_MB60\result_M2-vs-MB_MB-vs-MB60_2\pathway\hebing_id.htm#gene50) | 8 | 592 | 0.4072063 | 7.492596e-01 | ko00240 |
| 51 | [Circadian rhythm - plant](file:///E:\cao\MB%20转录组\最新M2_MB_MB60\result_M2-vs-MB_MB-vs-MB60_2\pathway\hebing_id.htm#gene51) | 6 | 448 | 0.4418908 | 7.913295e-01 | ko04712 |
| 52 | [Carbon fixation in photosynthetic organisms](file:///E:\cao\MB%20转录组\最新M2_MB_MB60\result_M2-vs-MB_MB-vs-MB60_2\pathway\hebing_id.htm#gene52) | 3 | 207 | 0.4472732 | 7.913295e-01 | ko00710 |
| 53 | [Spliceosome](file:///E:\cao\MB%20转录组\最新M2_MB_MB60\result_M2-vs-MB_MB-vs-MB60_2\pathway\hebing_id.htm#gene53) | 19 | 1545 | 0.4733496 | 8.128687e-01 | ko03040 |
| 54 | [Fructose and mannose metabolism](file:///E:\cao\MB%20转录组\最新M2_MB_MB60\result_M2-vs-MB_MB-vs-MB60_2\pathway\hebing_id.htm#gene54) | 3 | 218 | 0.4809164 | 8.128687e-01 | ko00051 |
| 55 | [Citrate cycle (TCA cycle)](file:///E:\cao\MB%20转录组\最新M2_MB_MB60\result_M2-vs-MB_MB-vs-MB60_2\pathway\hebing_id.htm#gene55) | 2 | 137 | 0.4859541 | 8.128687e-01 | ko00020 |
| 56 | [Pentose phosphate pathway](file:///E:\cao\MB%20转录组\最新M2_MB_MB60\result_M2-vs-MB_MB-vs-MB60_2\pathway\hebing_id.htm#gene56) | 3 | 225 | 0.5018162 | 8.244123e-01 | ko00030 |
| 57 | [Selenocompound metabolism](file:///E:\cao\MB%20转录组\最新M2_MB_MB60\result_M2-vs-MB_MB-vs-MB60_2\pathway\hebing_id.htm#gene57) | 1 | 63 | 0.5296234 | 8.400923e-01 | ko00450 |
| 58 | [Arachidonic acid metabolism](file:///E:\cao\MB%20转录组\最新M2_MB_MB60\result_M2-vs-MB_MB-vs-MB60_2\pathway\hebing_id.htm#gene58) | 1 | 63 | 0.5296234 | 8.400923e-01 | ko00590 |
| 59 | [Taurine and hypotaurine metabolism](file:///E:\cao\MB%20转录组\最新M2_MB_MB60\result_M2-vs-MB_MB-vs-MB60_2\pathway\hebing_id.htm#gene59) | 1 | 66 | 0.5462316 | 8.517510e-01 | ko00430 |
| 60 | [Nitrogen metabolism](file:///E:\cao\MB%20转录组\最新M2_MB_MB60\result_M2-vs-MB_MB-vs-MB60_2\pathway\hebing_id.htm#gene60) | 2 | 163 | 0.5791275 | 8.879955e-01 | ko00910 |
| 61 | [Alanine, aspartate and glutamate metabolism](file:///E:\cao\MB%20转录组\最新M2_MB_MB60\result_M2-vs-MB_MB-vs-MB60_2\pathway\hebing_id.htm#gene61) | 2 | 170 | 0.6020045 | 9.079412e-01 | ko00250 |
| 62 | [Valine, leucine and isoleucine biosynthesis](file:///E:\cao\MB%20转录组\最新M2_MB_MB60\result_M2-vs-MB_MB-vs-MB60_2\pathway\hebing_id.htm#gene62) | 1 | 87 | 0.6472071 | 9.127567e-01 | ko00290 |
| 63 | [Mismatch repair](file:///E:\cao\MB%20转录组\最新M2_MB_MB60\result_M2-vs-MB_MB-vs-MB60_2\pathway\hebing_id.htm#gene63) | 2 | 188 | 0.6564814 | 9.127567e-01 | ko03430 |
| 64 | [Brassinosteroid biosynthesis](file:///E:\cao\MB%20转录组\最新M2_MB_MB60\result_M2-vs-MB_MB-vs-MB60_2\pathway\hebing_id.htm#gene64) | 1 | 90 | 0.6596713 | 9.127567e-01 | ko00905 |
| 65 | [Base excision repair](file:///E:\cao\MB%20转录组\最新M2_MB_MB60\result_M2-vs-MB_MB-vs-MB60_2\pathway\hebing_id.htm#gene65) | 2 | 190 | 0.6621519 | 9.127567e-01 | ko03410 |
| 66 | [Biosynthesis of unsaturated fatty acids](file:///E:\cao\MB%20转录组\最新M2_MB_MB60\result_M2-vs-MB_MB-vs-MB60_2\pathway\hebing_id.htm#gene66) | 1 | 94 | 0.6756094 | 9.127567e-01 | ko01040 |
| 67 | [Phenylalanine metabolism](file:///E:\cao\MB%20转录组\最新M2_MB_MB60\result_M2-vs-MB_MB-vs-MB60_2\pathway\hebing_id.htm#gene67) | 2 | 197 | 0.6814075 | 9.127567e-01 | ko00360 |
| 68 | [Glycolysis / Gluconeogenesis](file:///E:\cao\MB%20转录组\最新M2_MB_MB60\result_M2-vs-MB_MB-vs-MB60_2\pathway\hebing_id.htm#gene68) | 5 | 483 | 0.682229 | 9.127567e-01 | ko00010 |
| 69 | [RNA polymerase](file:///E:\cao\MB%20转录组\最新M2_MB_MB60\result_M2-vs-MB_MB-vs-MB60_2\pathway\hebing_id.htm#gene69) | 3 | 297 | 0.6877069 | 9.127567e-01 | ko03020 |
| 70 | [Histidine metabolism](file:///E:\cao\MB%20转录组\最新M2_MB_MB60\result_M2-vs-MB_MB-vs-MB60_2\pathway\hebing_id.htm#gene70) | 1 | 99 | 0.6944888 | 9.127567e-01 | ko00340 |
| 71 | [Natural killer cell mediated cytotoxicity](file:///E:\cao\MB%20转录组\最新M2_MB_MB60\result_M2-vs-MB_MB-vs-MB60_2\pathway\hebing_id.htm#gene71) | 2 | 212 | 0.7196477 | 9.325012e-01 | ko04650 |
| 72 | [Purine metabolism](file:///E:\cao\MB%20转录组\最新M2_MB_MB60\result_M2-vs-MB_MB-vs-MB60_2\pathway\hebing_id.htm#gene72) | 7 | 703 | 0.7324996 | 9.359717e-01 | ko00230 |
| 73 | [Glutathione metabolism](file:///E:\cao\MB%20转录组\最新M2_MB_MB60\result_M2-vs-MB_MB-vs-MB60_2\pathway\hebing_id.htm#gene73) | 2 | 234 | 0.7686857 | 9.571260e-01 | ko00480 |
| 74 | [SNARE interactions in vesicular transport](file:///E:\cao\MB%20转录组\最新M2_MB_MB60\result_M2-vs-MB_MB-vs-MB60_2\pathway\hebing_id.htm#gene74) | 1 | 138 | 0.8086673 | 9.571260e-01 | ko04130 |
| 75 | [Pantothenate and CoA biosynthesis](file:///E:\cao\MB%20转录组\最新M2_MB_MB60\result_M2-vs-MB_MB-vs-MB60_2\pathway\hebing_id.htm#gene75) | 1 | 141 | 0.815436 | 9.571260e-01 | ko00770 |
| 76 | [Ribosome biogenesis in eukaryotes](file:///E:\cao\MB%20转录组\最新M2_MB_MB60\result_M2-vs-MB_MB-vs-MB60_2\pathway\hebing_id.htm#gene76) | 5 | 581 | 0.8222498 | 9.571260e-01 | ko03008 |
| 77 | [Galactose metabolism](file:///E:\cao\MB%20转录组\最新M2_MB_MB60\result_M2-vs-MB_MB-vs-MB60_2\pathway\hebing_id.htm#gene77) | 3 | 375 | 0.824838 | 9.571260e-01 | ko00052 |
| 78 | [Zeatin biosynthesis](file:///E:\cao\MB%20转录组\最新M2_MB_MB60\result_M2-vs-MB_MB-vs-MB60_2\pathway\hebing_id.htm#gene78) | 2 | 271 | 0.8344101 | 9.571260e-01 | ko00908 |
| 79 | [Glycerolipid metabolism](file:///E:\cao\MB%20转录组\最新M2_MB_MB60\result_M2-vs-MB_MB-vs-MB60_2\pathway\hebing_id.htm#gene79) | 2 | 271 | 0.8344101 | 9.571260e-01 | ko00561 |
| 80 | [Steroid biosynthesis](file:///E:\cao\MB%20转录组\最新M2_MB_MB60\result_M2-vs-MB_MB-vs-MB60_2\pathway\hebing_id.htm#gene80) | 1 | 154 | 0.842112 | 9.571260e-01 | ko00100 |
| 81 | [Oxidative phosphorylation](file:///E:\cao\MB%20转录组\最新M2_MB_MB60\result_M2-vs-MB_MB-vs-MB60_2\pathway\hebing_id.htm#gene81) | 3 | 396 | 0.8515917 | 9.571260e-01 | ko00190 |
| 82 | [Glyoxylate and dicarboxylate metabolism](file:///E:\cao\MB%20转录组\最新M2_MB_MB60\result_M2-vs-MB_MB-vs-MB60_2\pathway\hebing_id.htm#gene82) | 1 | 160 | 0.8530906 | 9.571260e-01 | ko00630 |
| 83 | [Protein export](file:///E:\cao\MB%20转录组\最新M2_MB_MB60\result_M2-vs-MB_MB-vs-MB60_2\pathway\hebing_id.htm#gene83) | 1 | 167 | 0.8649397 | 9.587283e-01 | ko03060 |
| 84 | [Protein processing in endoplasmic reticulum](file:///E:\cao\MB%20转录组\最新M2_MB_MB60\result_M2-vs-MB_MB-vs-MB60_2\pathway\hebing_id.htm#gene84) | 9 | 1069 | 0.8910876 | 9.759531e-01 | ko04141 |
| 85 | [Nucleotide excision repair](file:///E:\cao\MB%20转录组\最新M2_MB_MB60\result_M2-vs-MB_MB-vs-MB60_2\pathway\hebing_id.htm#gene85) | 2 | 339 | 0.9129123 | 9.880933e-01 | ko03420 |
| 86 | [Homologous recombination](file:///E:\cao\MB%20转录组\最新M2_MB_MB60\result_M2-vs-MB_MB-vs-MB60_2\pathway\hebing_id.htm#gene86) | 1 | 220 | 0.928586 | 9.933711e-01 | ko03440 |
| 87 | [RNA degradation](file:///E:\cao\MB%20转录组\最新M2_MB_MB60\result_M2-vs-MB_MB-vs-MB60_2\pathway\hebing_id.htm#gene87) | 4 | 633 | 0.9443421 | 9.986146e-01 | ko03018 |
| 88 | [Aminoacyl-tRNA biosynthesis](file:///E:\cao\MB%20转录组\最新M2_MB_MB60\result_M2-vs-MB_MB-vs-MB60_2\pathway\hebing_id.htm#gene88) | 1 | 289 | 0.9688892 | 9.989985e-01 | ko00970 |
| 89 | [Phagosome](file:///E:\cao\MB%20转录组\最新M2_MB_MB60\result_M2-vs-MB_MB-vs-MB60_2\pathway\hebing_id.htm#gene89) | 1 | 324 | 0.979601 | 9.989985e-01 | ko04145 |
| 90 | [Peroxisome](file:///E:\cao\MB%20转录组\最新M2_MB_MB60\result_M2-vs-MB_MB-vs-MB60_2\pathway\hebing_id.htm#gene90) | 1 | 353 | 0.9856253 | 9.989985e-01 | ko04146 |
| 91 | [mRNA surveillance pathway](file:///E:\cao\MB%20转录组\最新M2_MB_MB60\result_M2-vs-MB_MB-vs-MB60_2\pathway\hebing_id.htm#gene91) | 3 | 688 | 0.9889103 | 9.989985e-01 | ko03015 |
| 92 | [RNA transport](file:///E:\cao\MB%20转录组\最新M2_MB_MB60\result_M2-vs-MB_MB-vs-MB60_2\pathway\hebing_id.htm#gene92) | 5 | 1224 | 0.9989985 | 9.989985e-01 | ko03013 |

a: the number of the differentially expressed unigenes that involved in the corresponding pathway; b: the number of the all-unigene that involved in the corresponding pathway; c: indicating a significantly enriched GO terms among the differentially expressed unigenes; d: indicating a significantly enriched pathway among the differentially expressed unigenes.
