# Supplementary figures and images for: Transcriptome, microRNA, and degradome analyses of the gene expression of Paulownia with phytoplamsa
Source: BMC Genomics. 2015 Nov 4;16:896. doi: 10.1186/s12864-015-2074-3 (PMC4634154; doi:10.1186/s12864-015-2074-3)

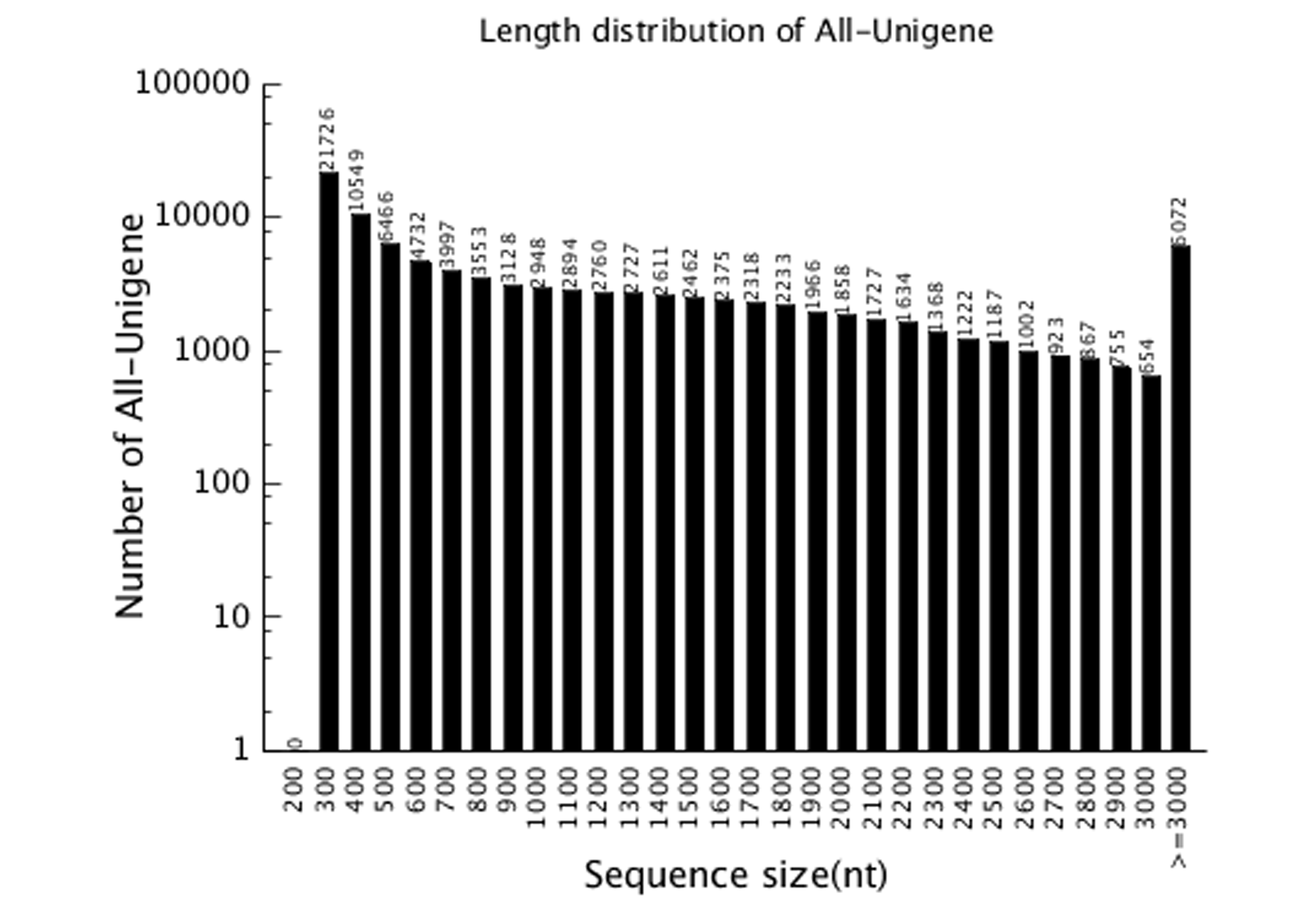

Supplement: Additional file 21: Figure S1. — Length distribution of all-unigene in P. tomentosa. (TIF 5.5 MB) [file 12864_2015_2074_MOESM21_ESM.tif]

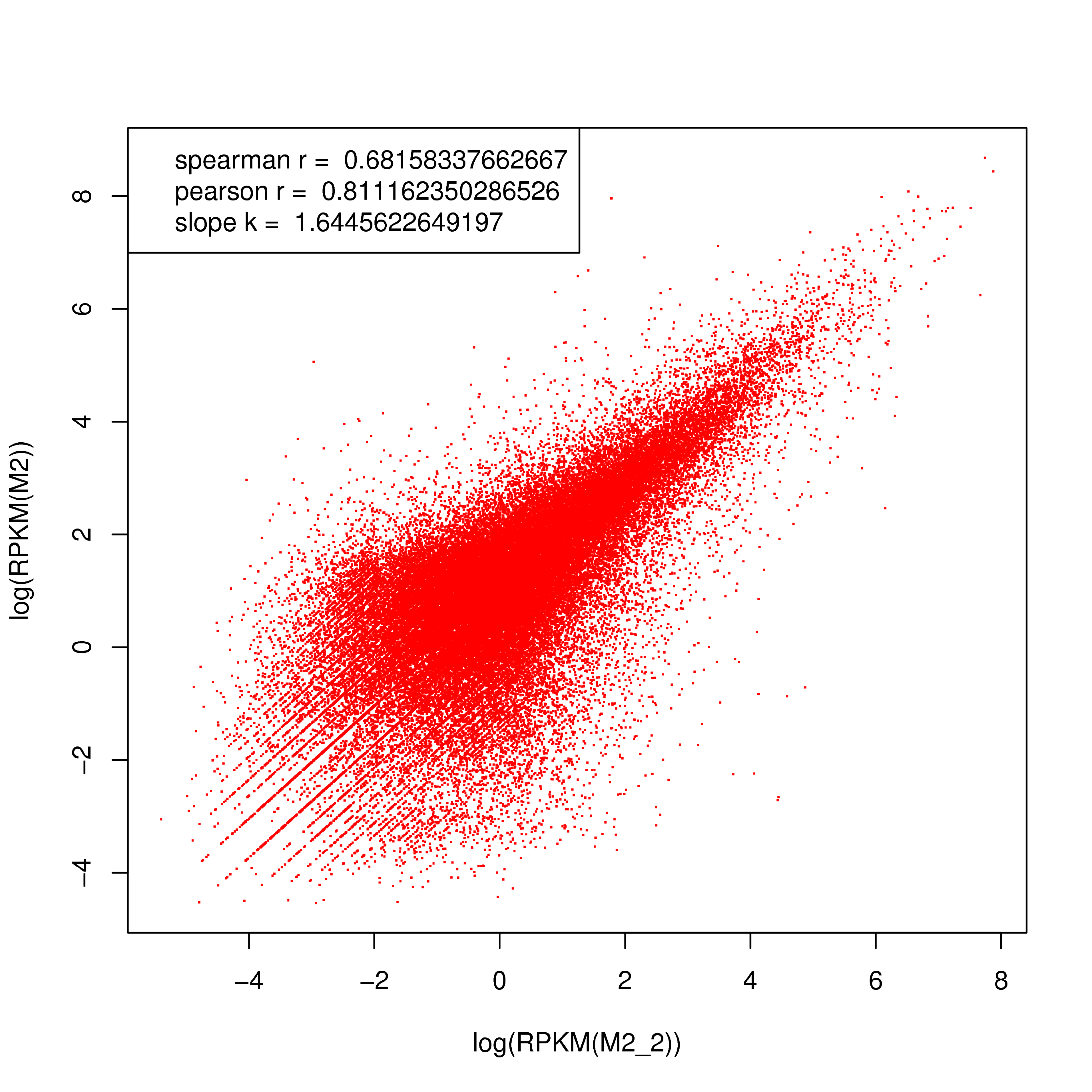

Supplement: Additional file 22: Figure S2. — Correlation coefficients of the gene expression of duplicate samples. Y-axis represents the logarithmic value of HP expression, while X-axis represents the logarithmic value of the corresponding duplicate samples. (TIF 3.21 MB) [file 12864_2015_2074_MOESM22_ESM.tif]

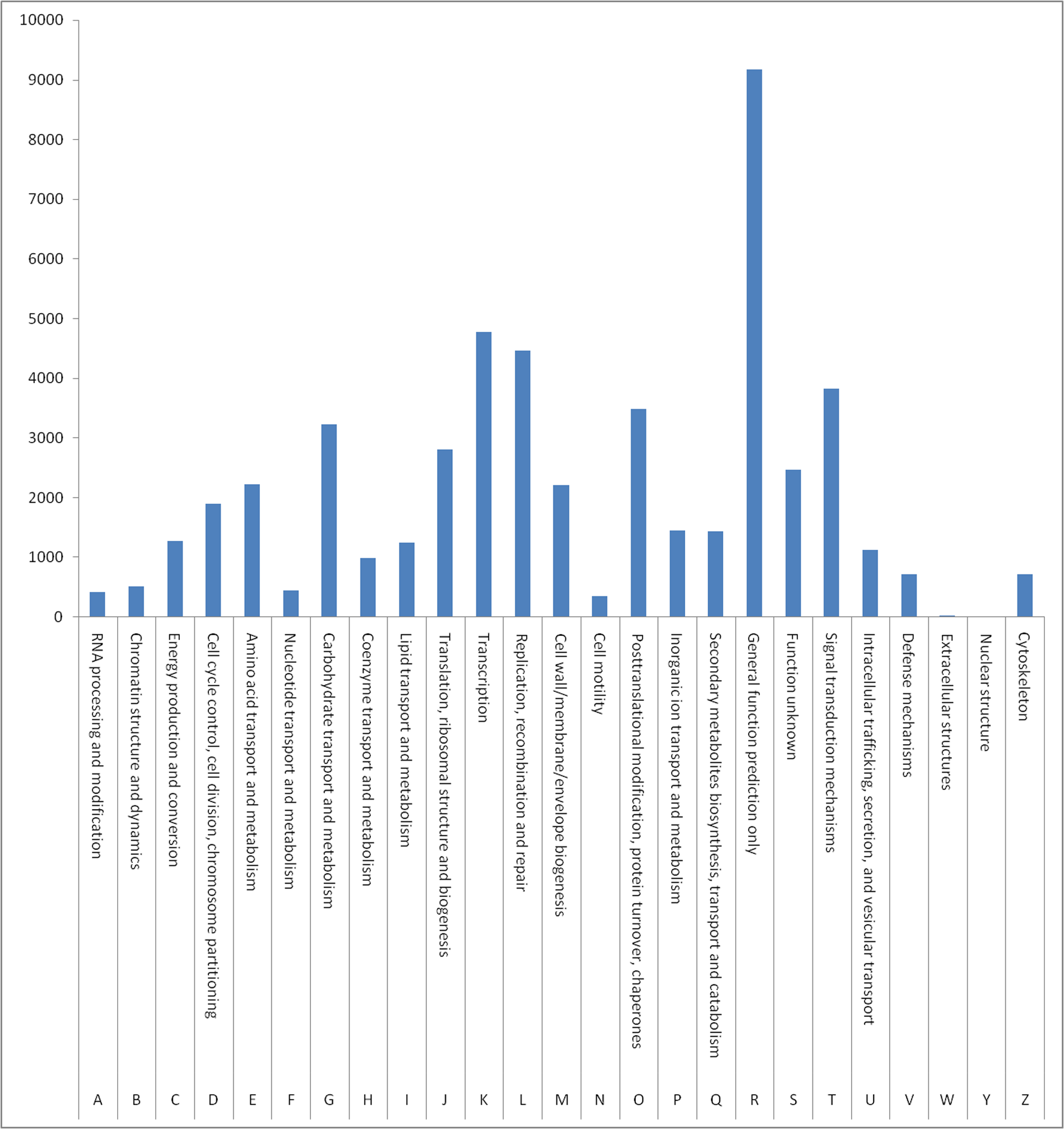

Supplement: Additional file 23: Figure S3. — COG function classification of all-unigene of P. tomentosa. (TIF 12.2 MB) [file 12864_2015_2074_MOESM23_ESM.tif]

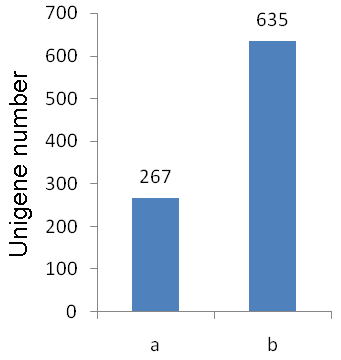

Supplement: Additional file 25: Figure S5. — DEG analysis of P. tomentosa a: DEGs in PIP vs. HP up-regulated and down-regulated in PIP-60 vs. PIP. b: DEGs in PIP vs. HP down-regulated and up-regulated in PIP-60 vs. PIP. (TIF 39.7 kb) [file 12864_2015_2074_MOESM25_ESM.tif]
