# Supplementary material for: Transcriptome, microRNA, and degradome analyses of the gene expression of Paulownia with phytoplamsa
Source: BMC Genomics. 2015 Nov 4;16:896. doi: 10.1186/s12864-015-2074-3 (PMC4634154; doi:10.1186/s12864-015-2074-3)
Supplement: Additional file 24: Figure S4. — Hairpin structures of P. tometosa miRNAs Pau-mR1a-i have the same mature miRNA sequence; pau-mR3a/b have the same mature miRNA sequence; pau-mR14a/b have the same mature miRNA sequence; pau-mR16a-c have the same mature miRNA sequence; pau-mR17a/b have the same mature miRNA sequence; pau-mR20a/b have the same mature miRNA sequence. (DOC 1.61 MB) [file 12864_2015_2074_MOESM24_ESM.doc]

**Additional file 24: Figure S4 Hairpin structures of** ***P. tometosa* miRNAs**


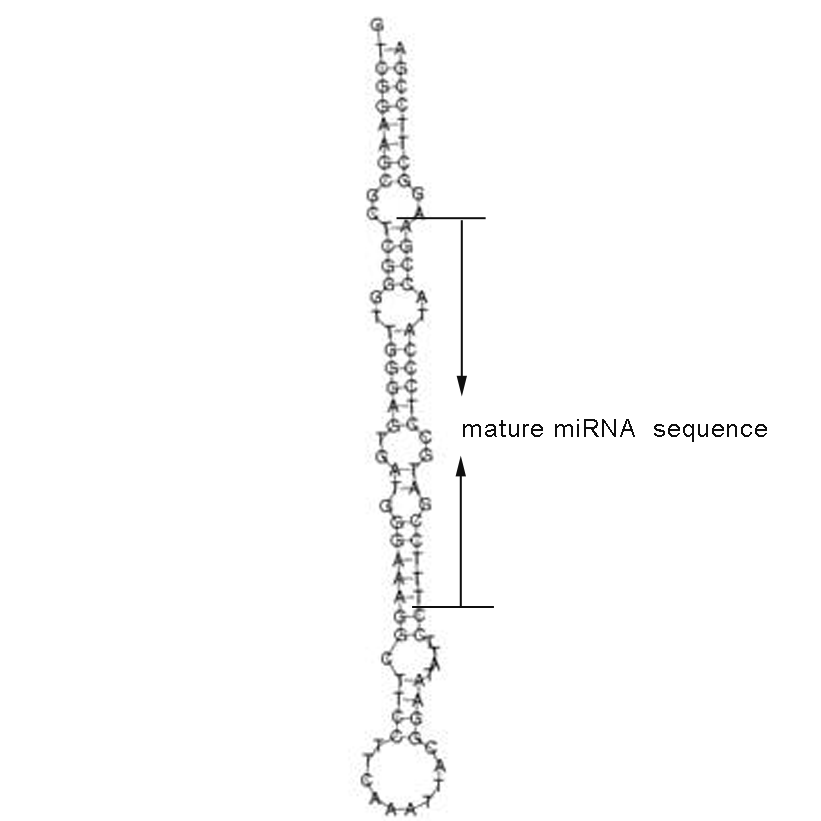

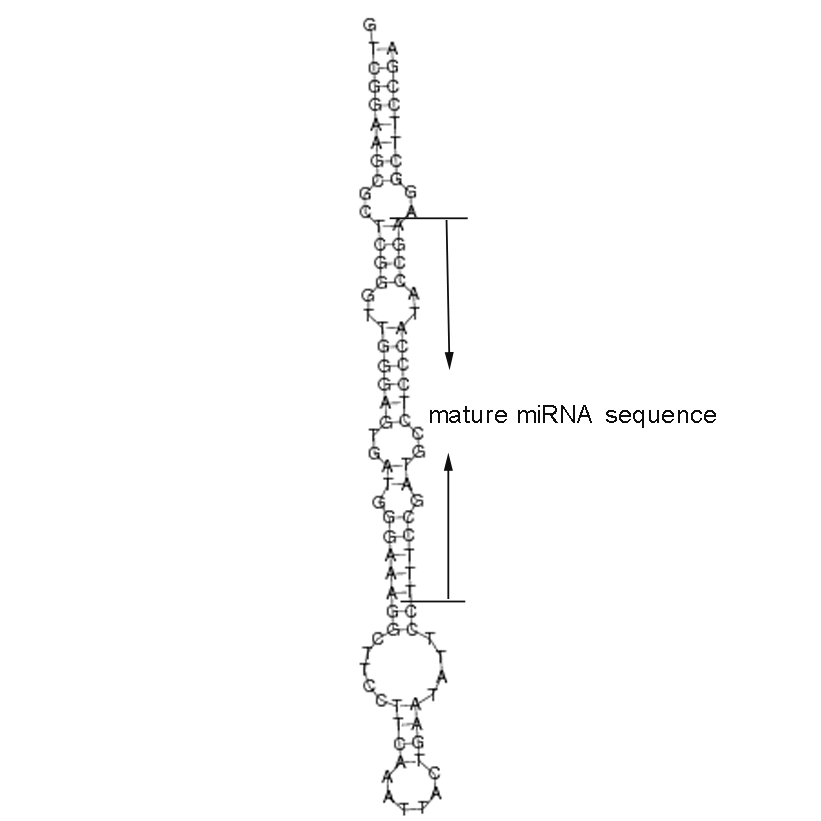


Pau-mR1a Pau-mR1b


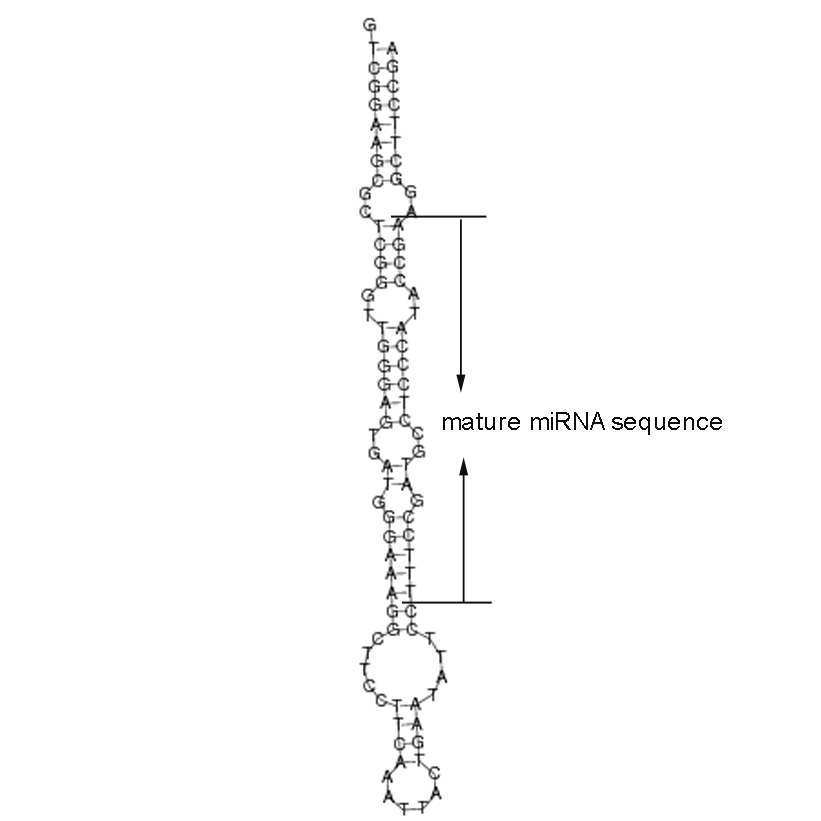

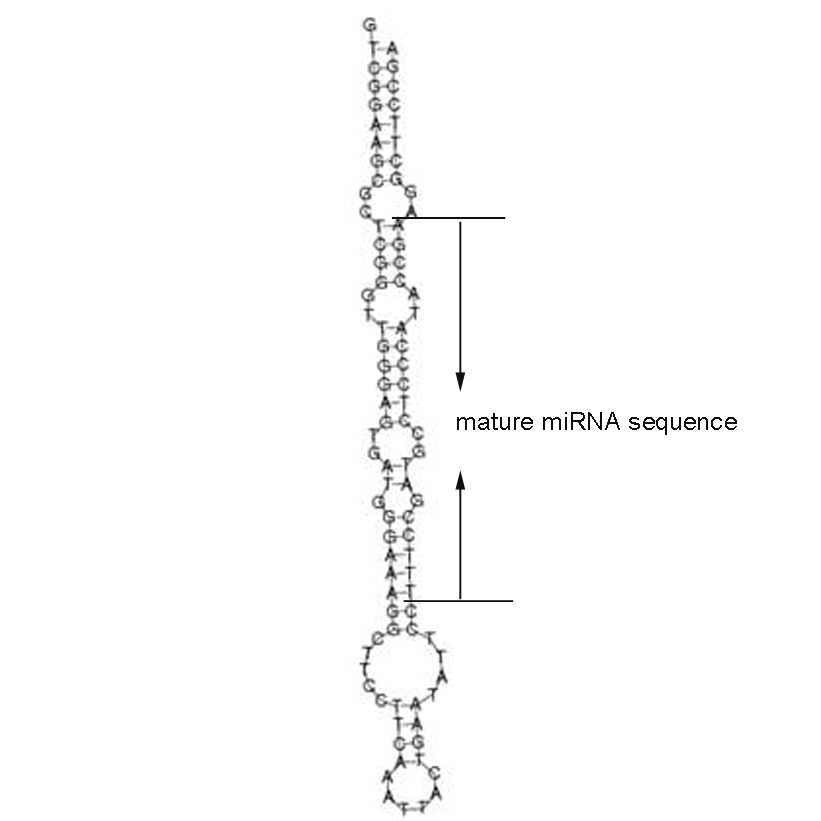


Pau-mR1c Pau-mR1d


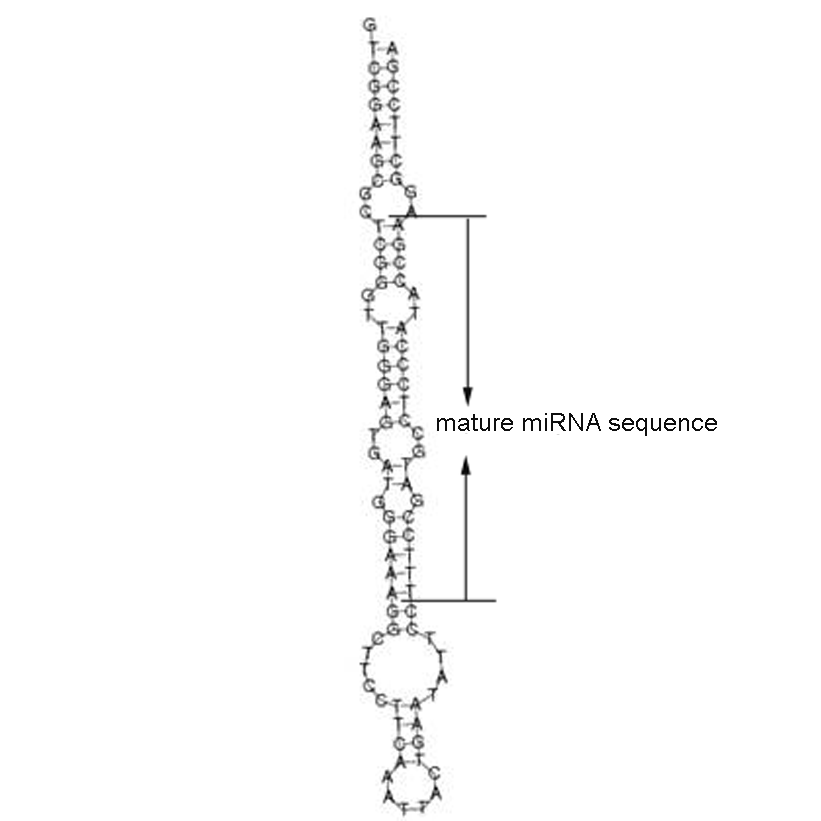

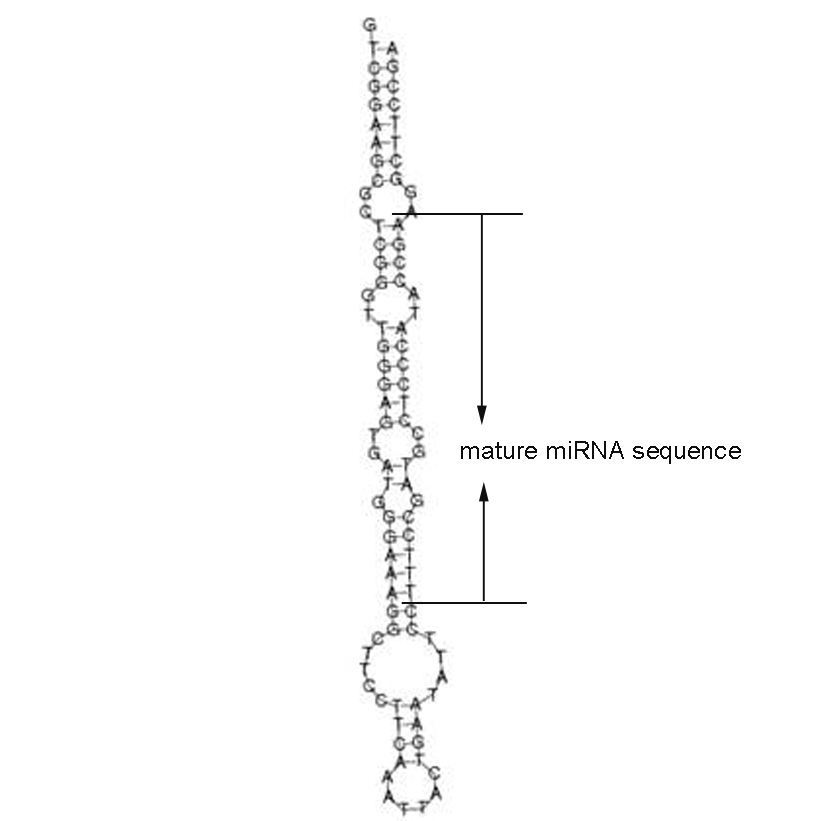


Pau-mR1e Pau-mR1f


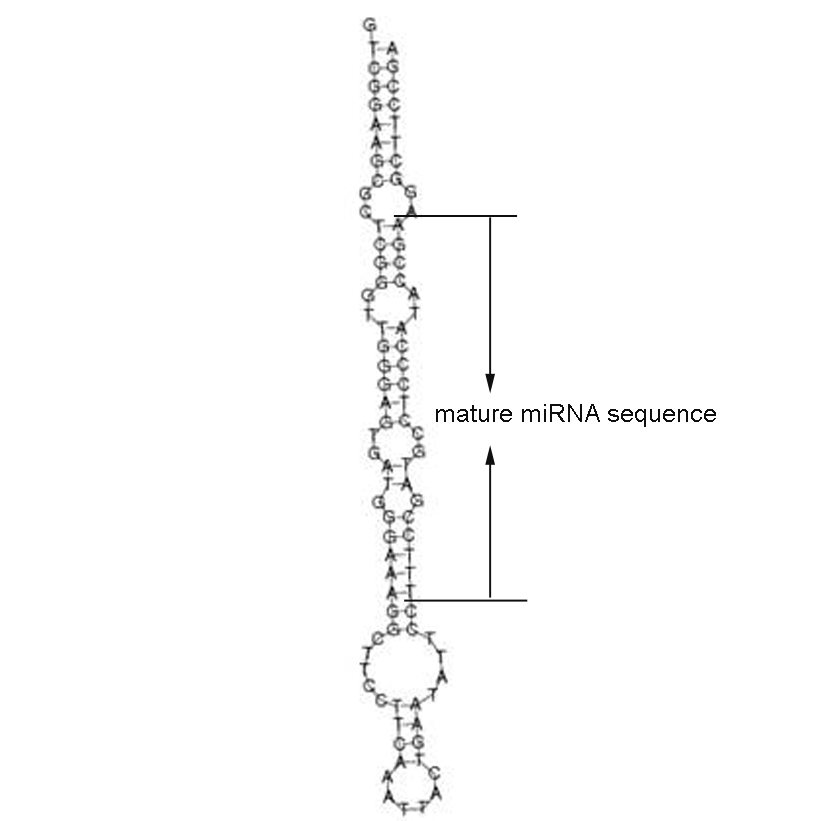

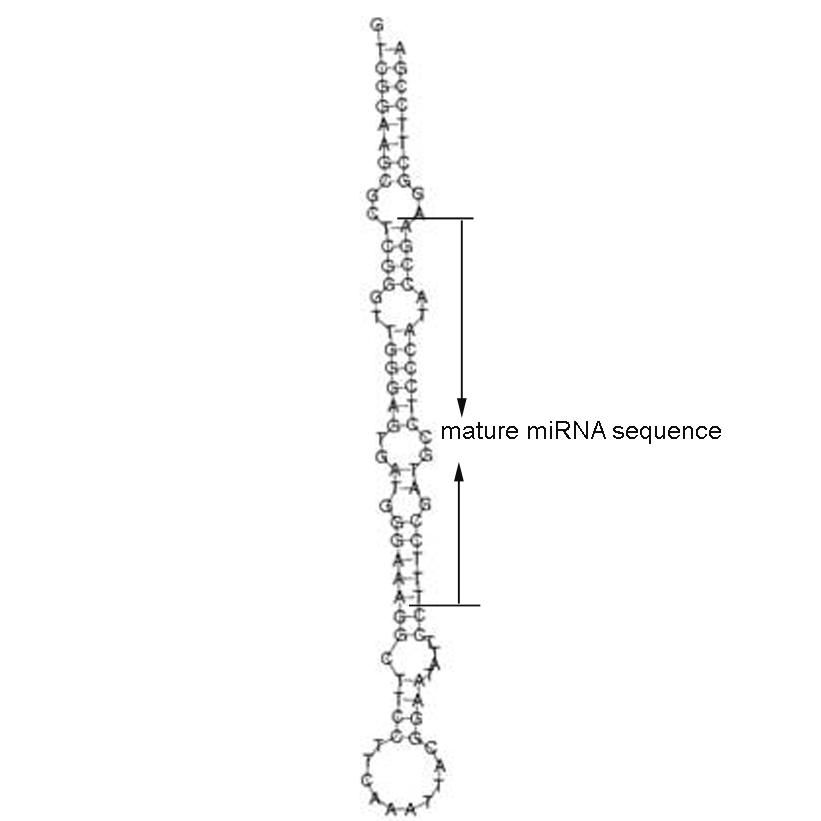


Pau-mR1g Pau-mR1h


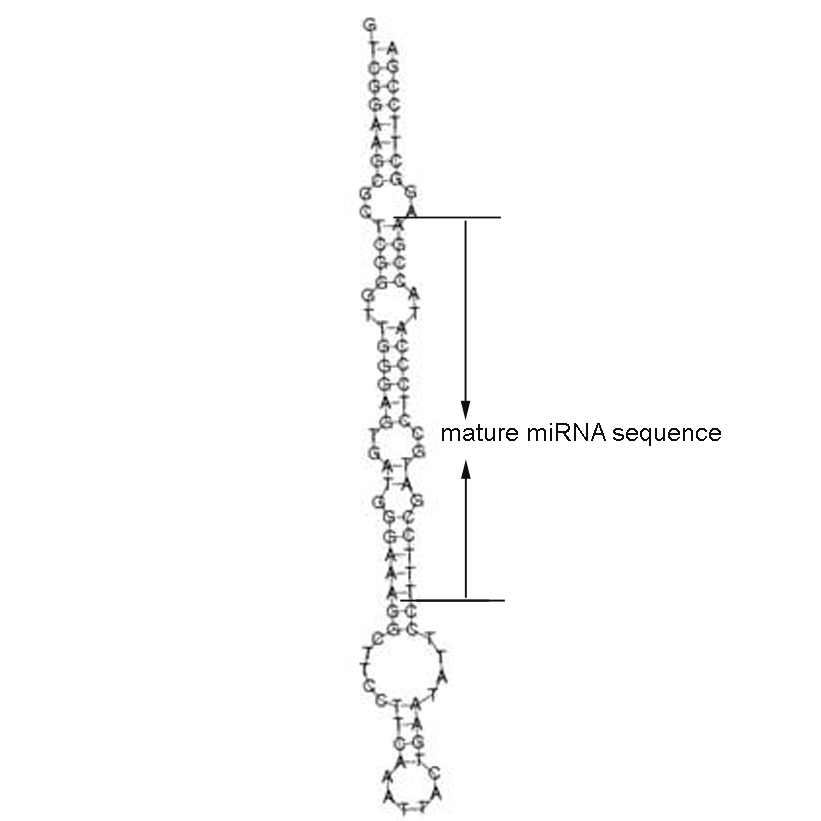

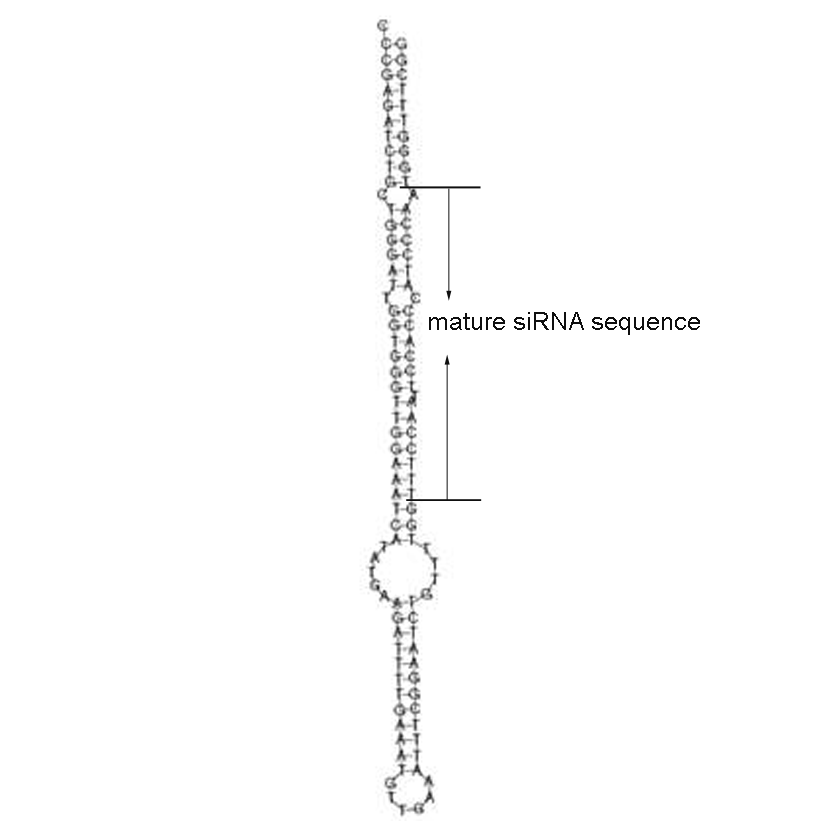


Pau-mR1i Pau-mR2


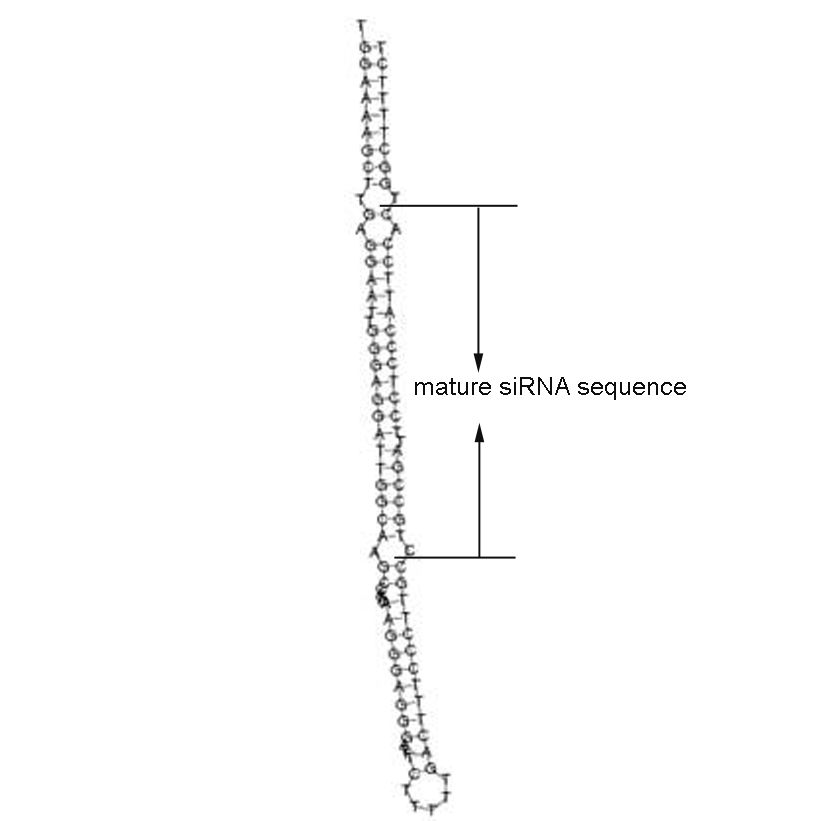

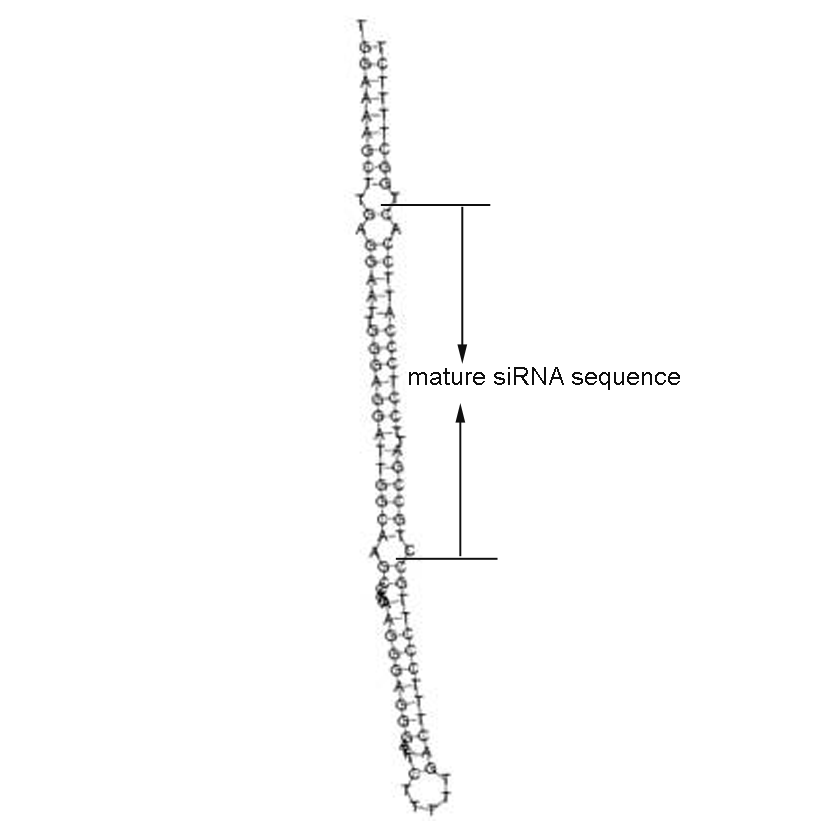


Pau-mR3a Pau-mR3b


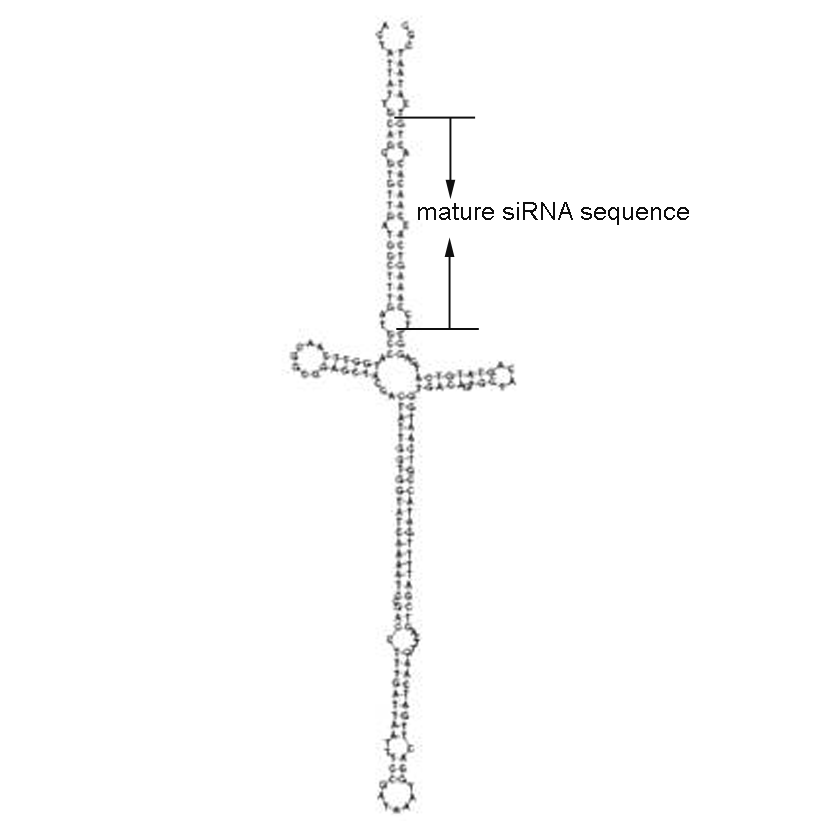

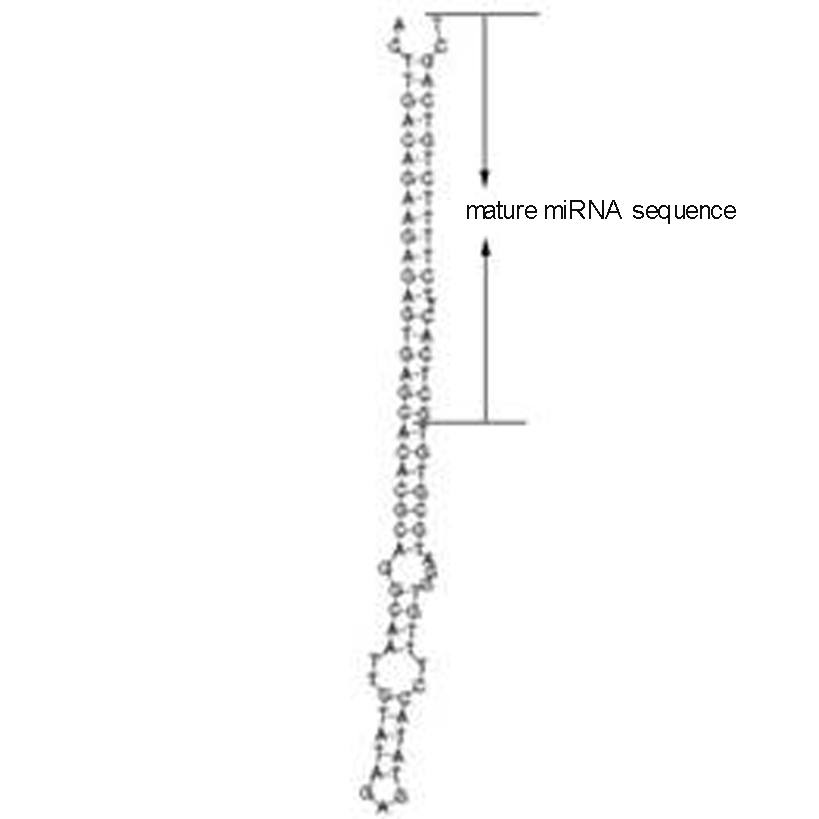


Pau-mR4 Pau-mR5


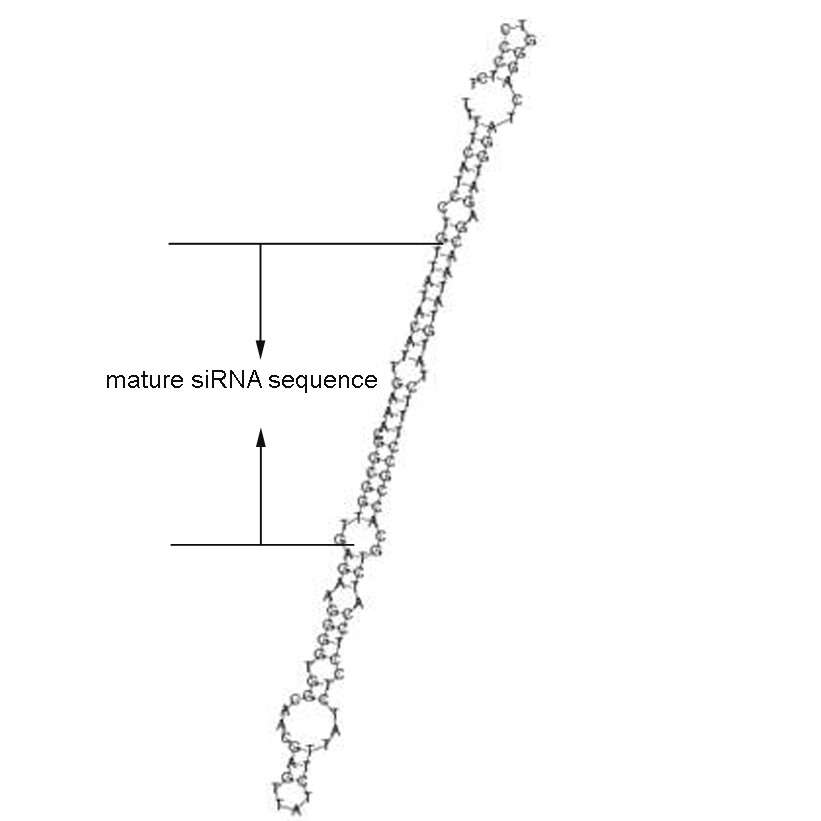

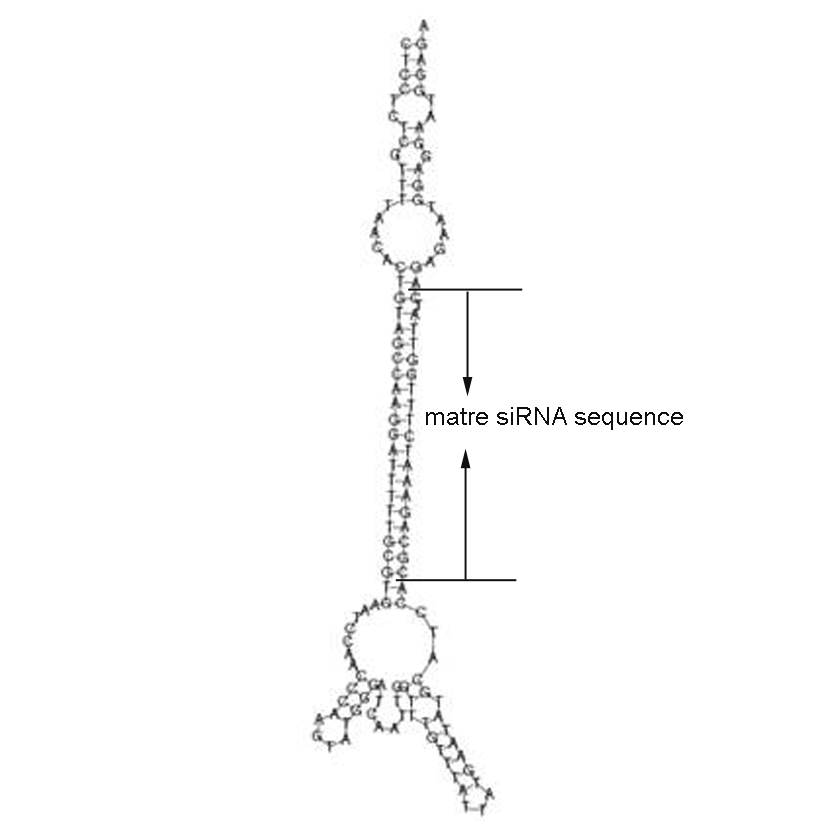


Pau-mR6 Pau-mR7


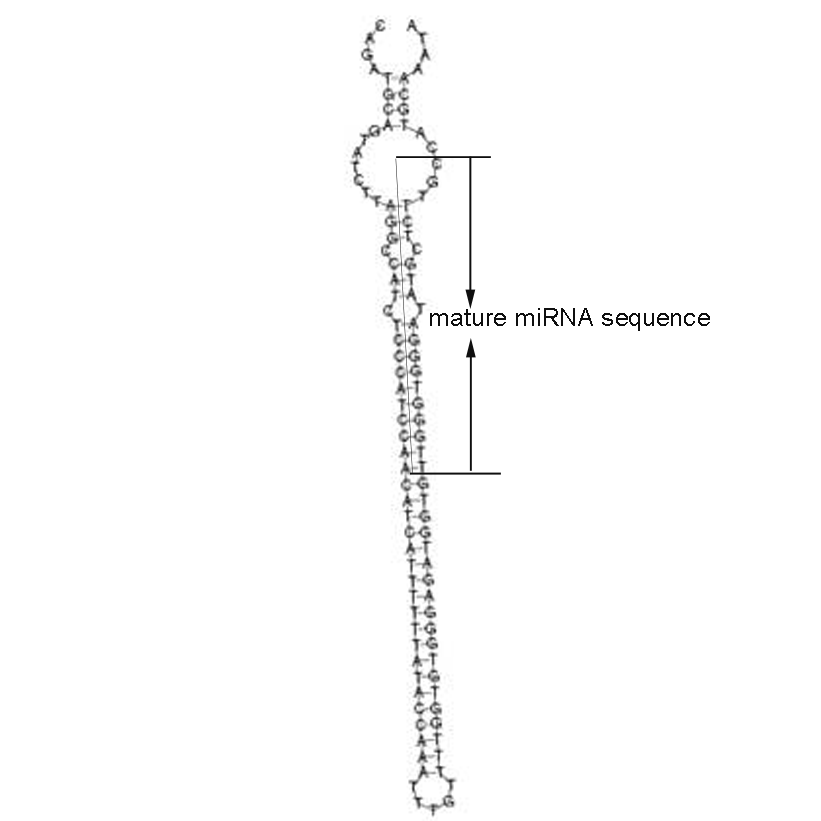

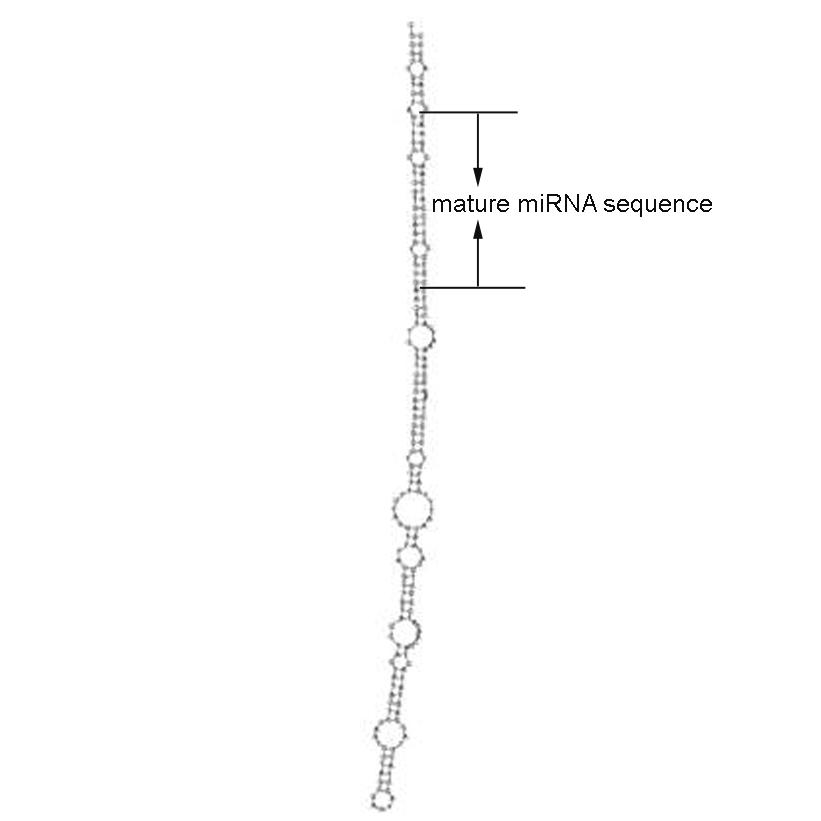


Pau-mR8 Pau-mR9


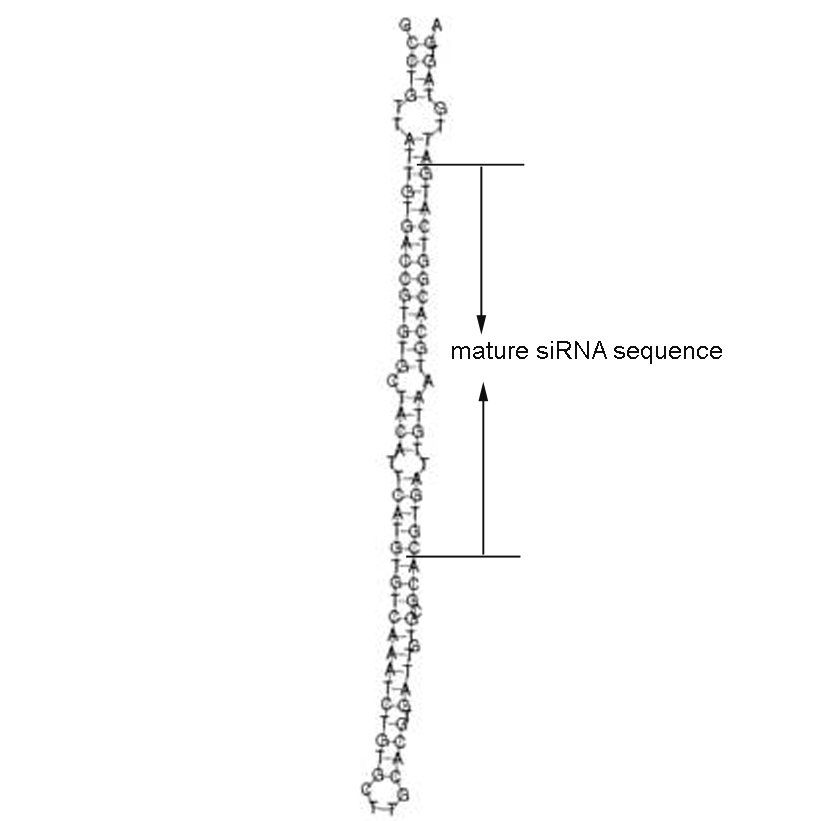

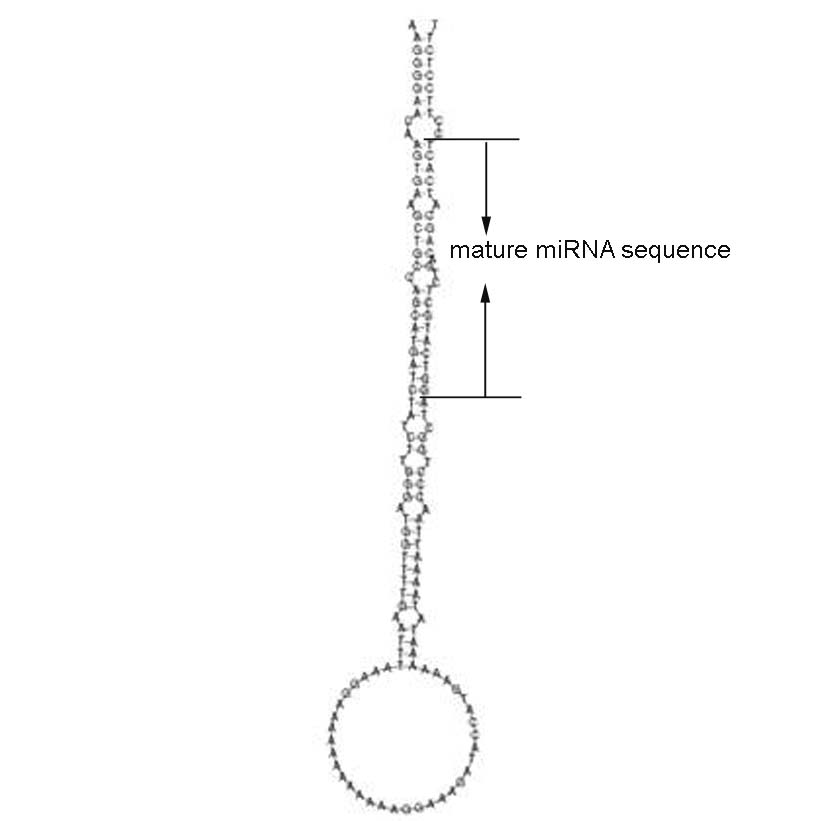


Pau-mR10 Pau-mR11


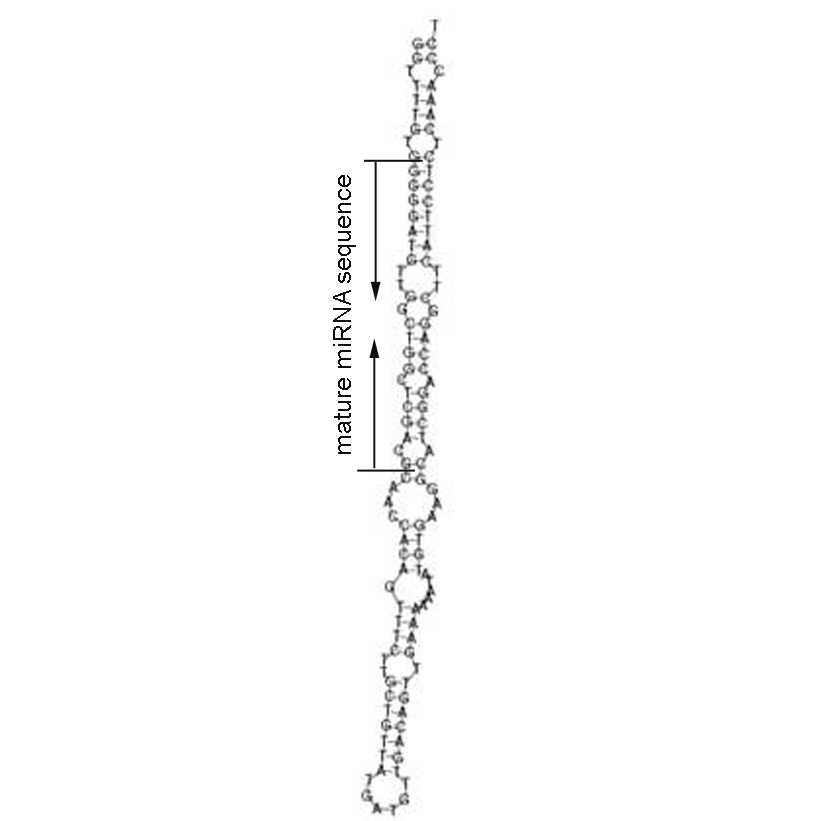

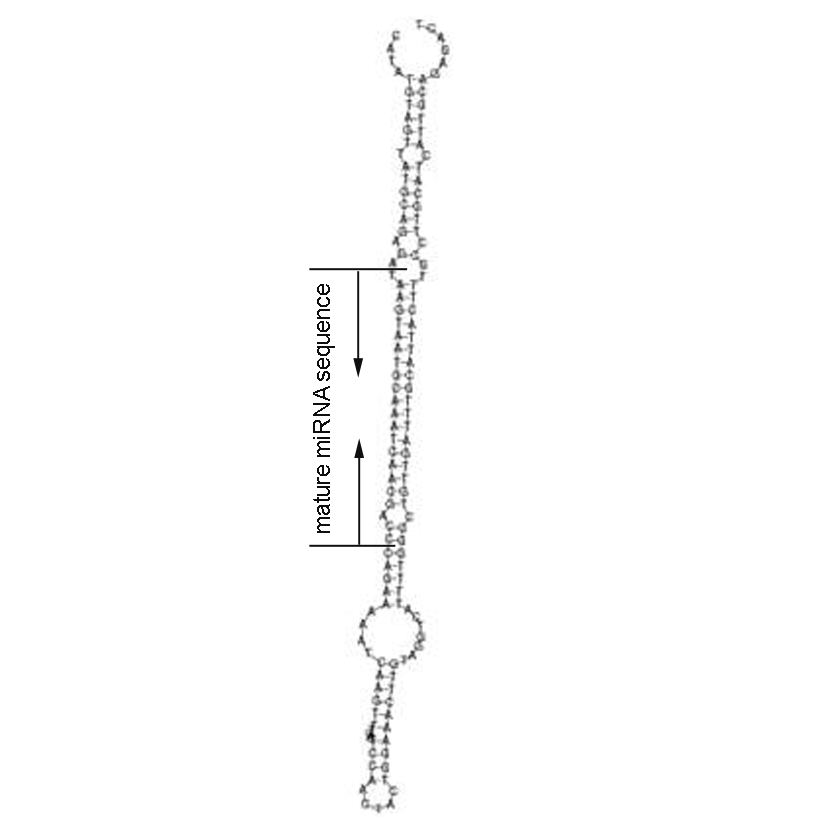


Pau-mR12 Pau-mR13


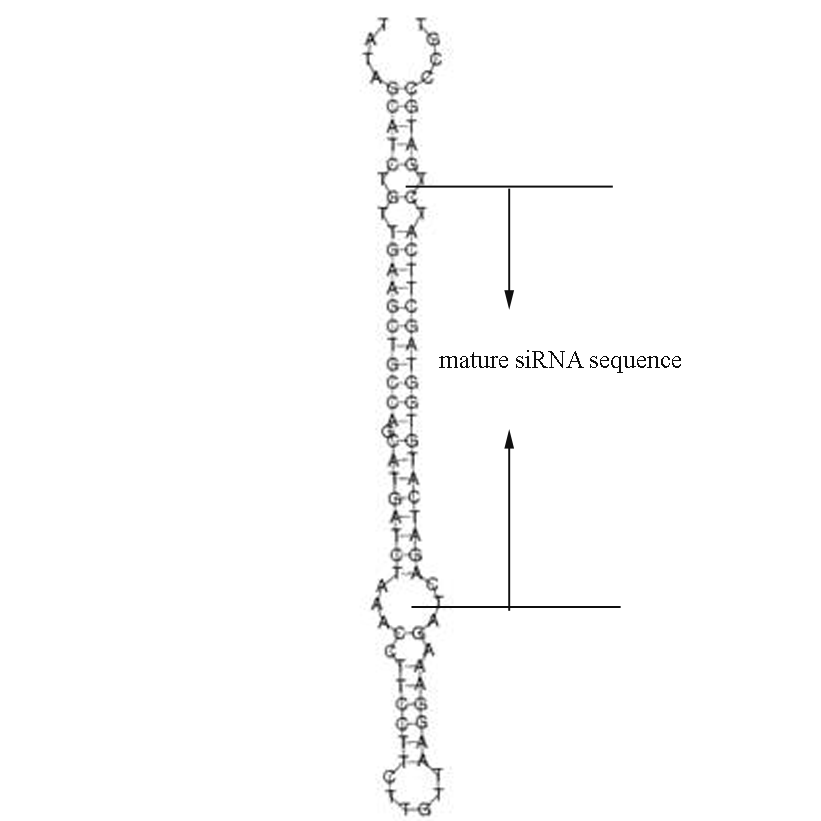

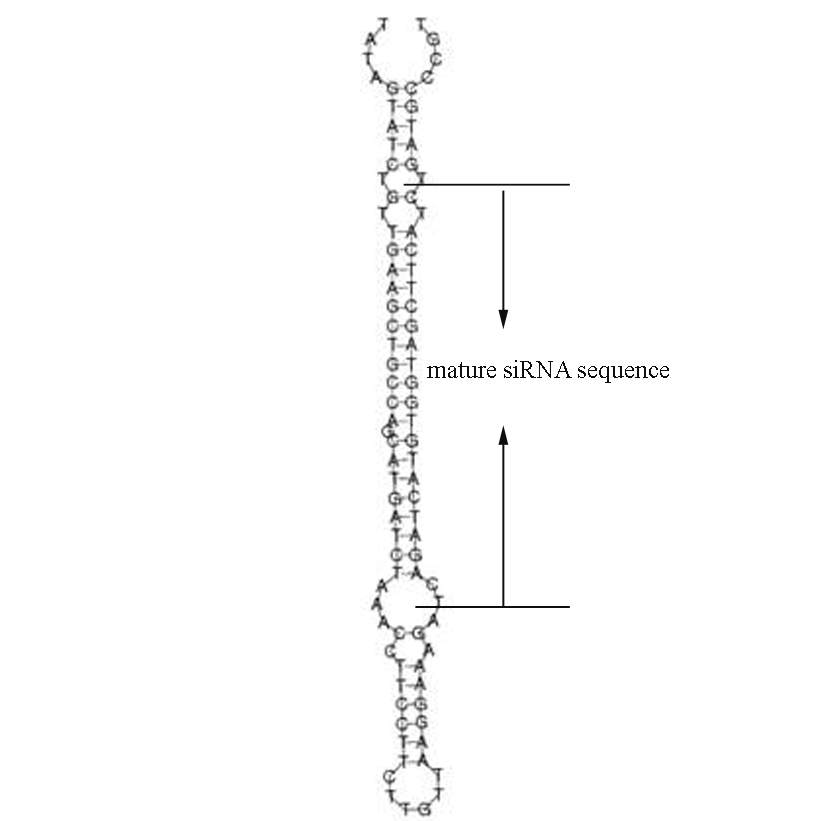


Pau-mR14a Pau-mR14b


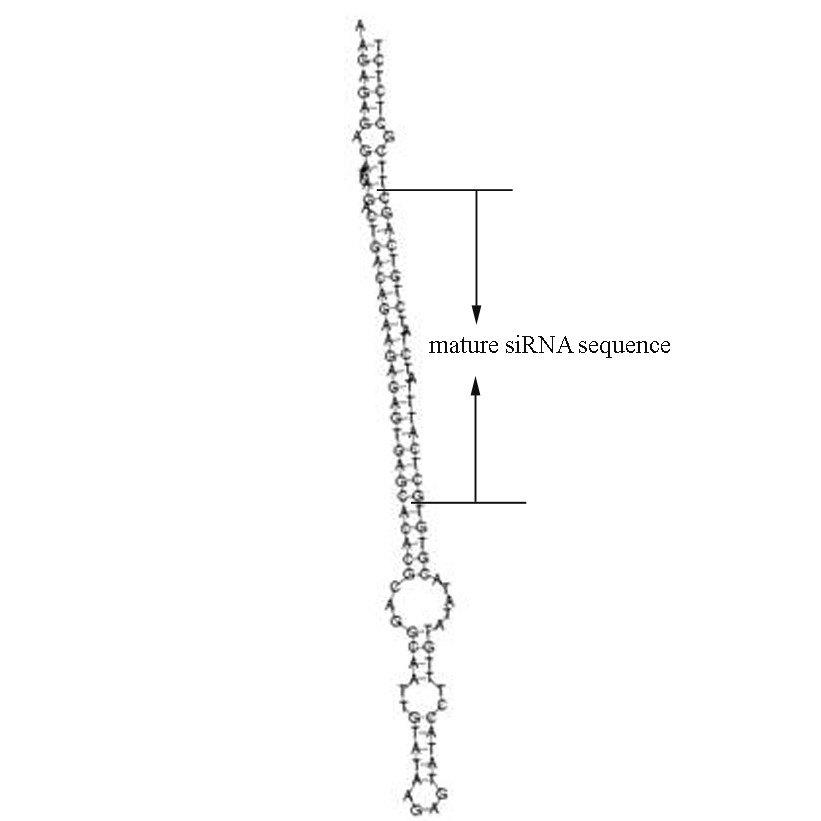

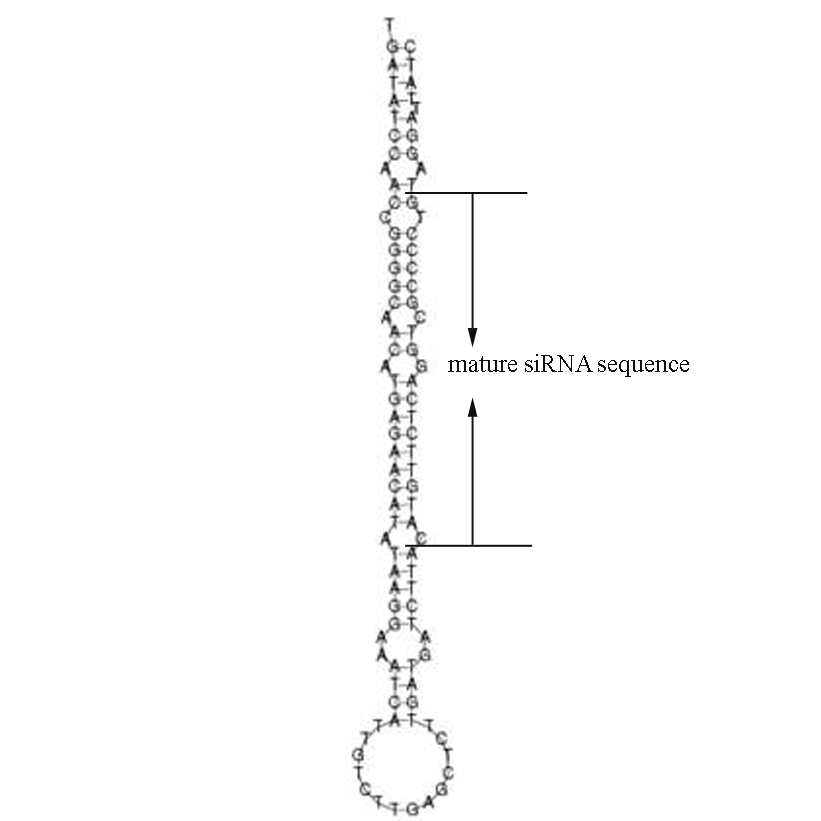


Pau-mR15 Pau-mR16a


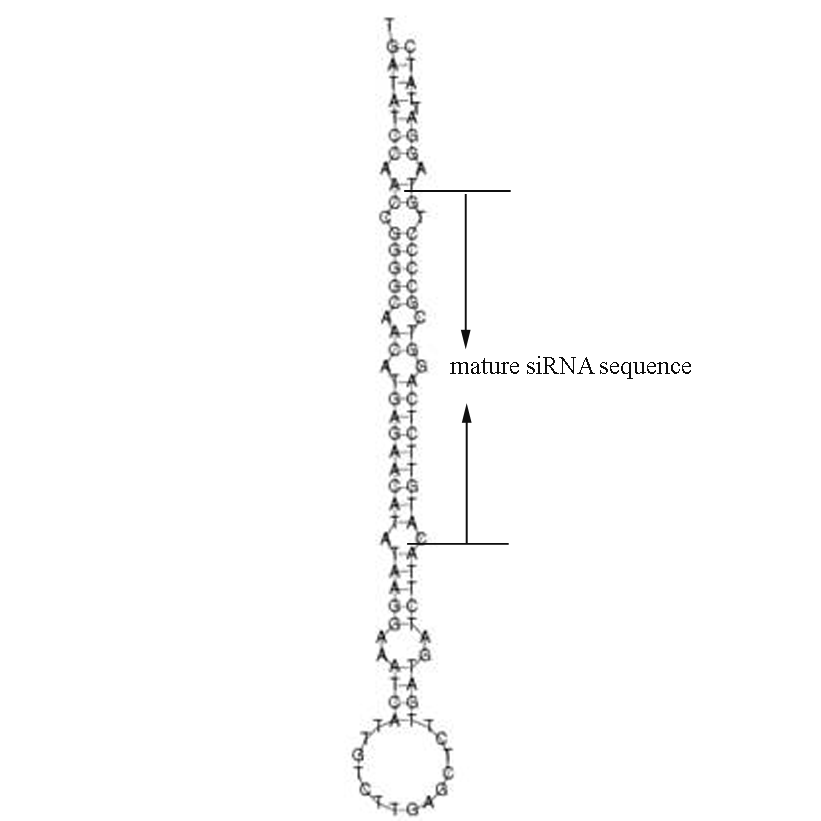

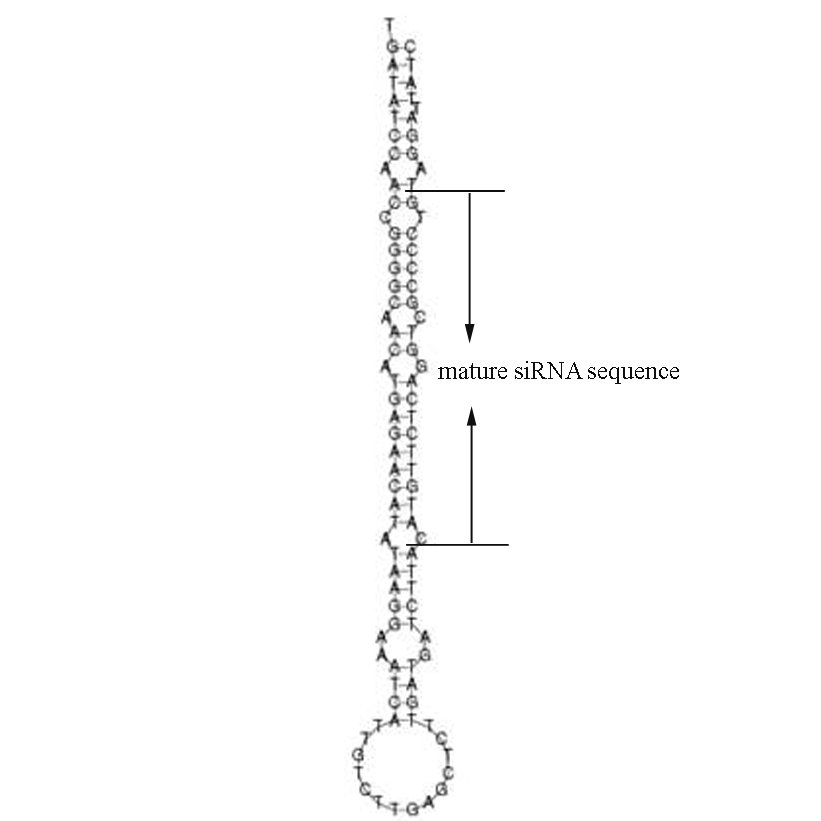


Pau-mR16b Pau-mR16c


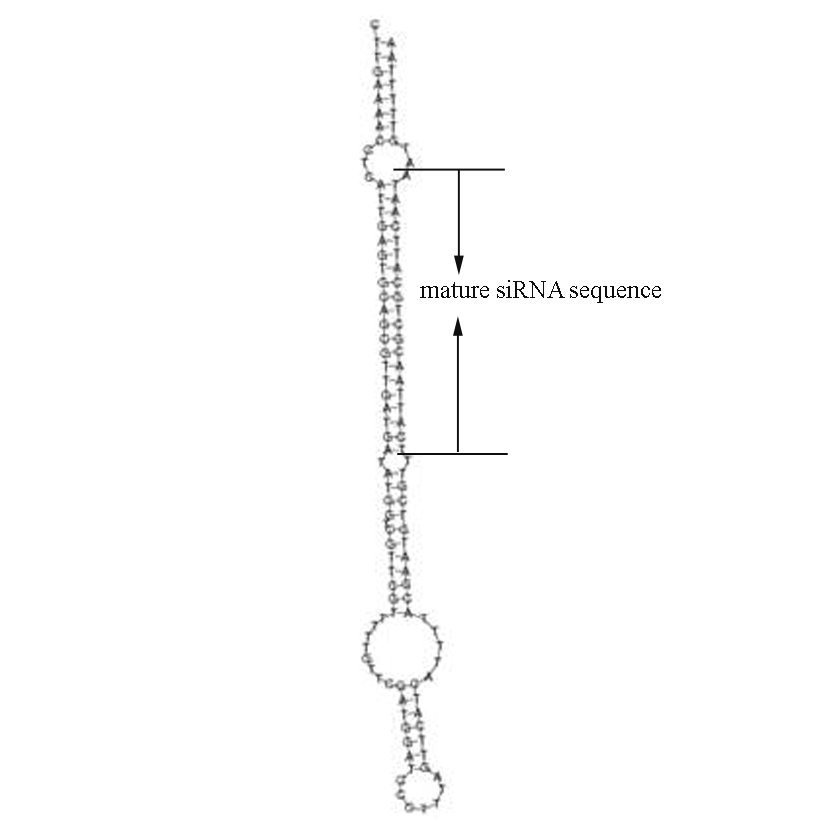

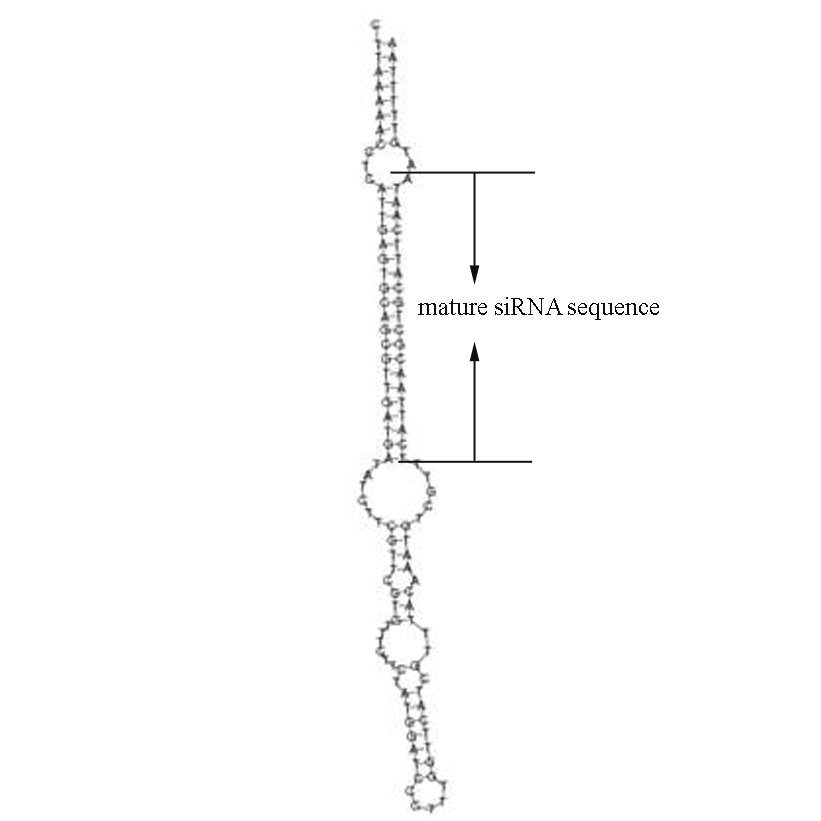


Pau-mR17a Pau-mR17b


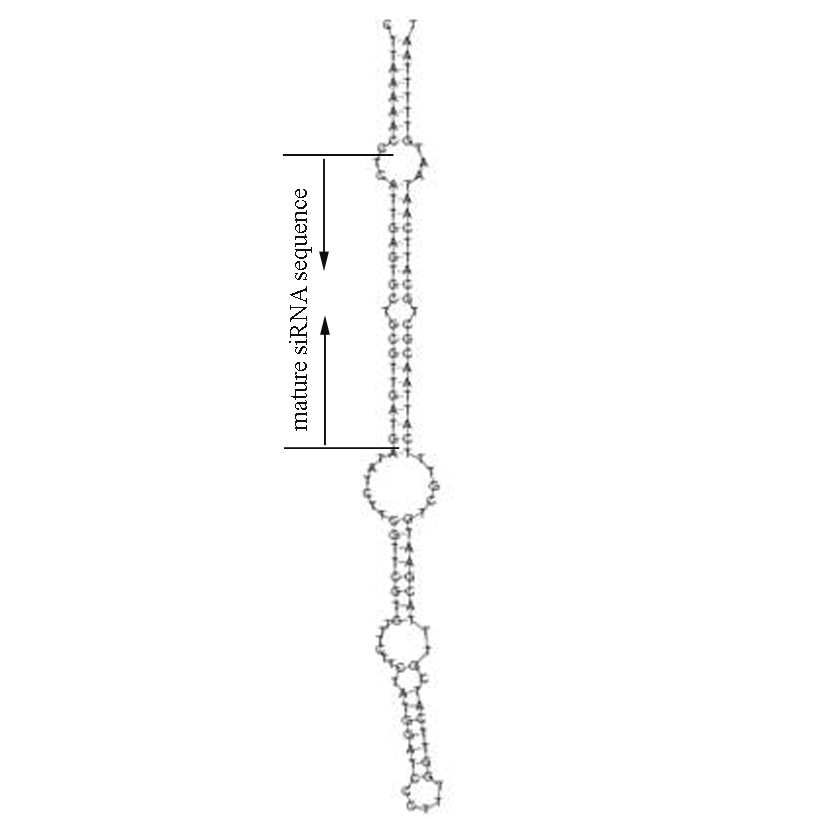

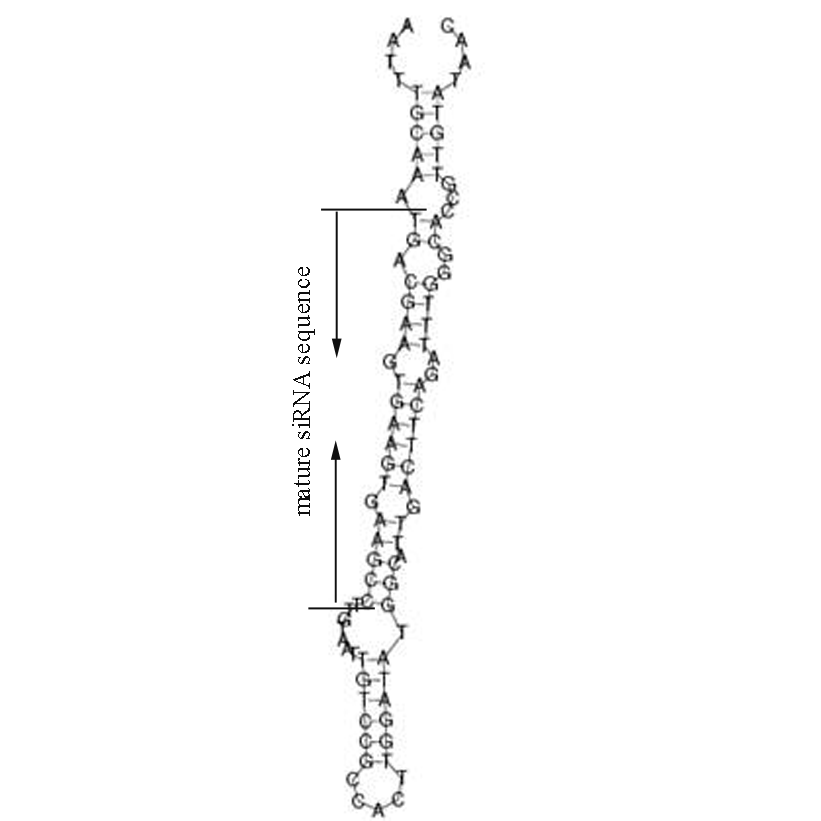


Pau-mR18 Pau-mR19


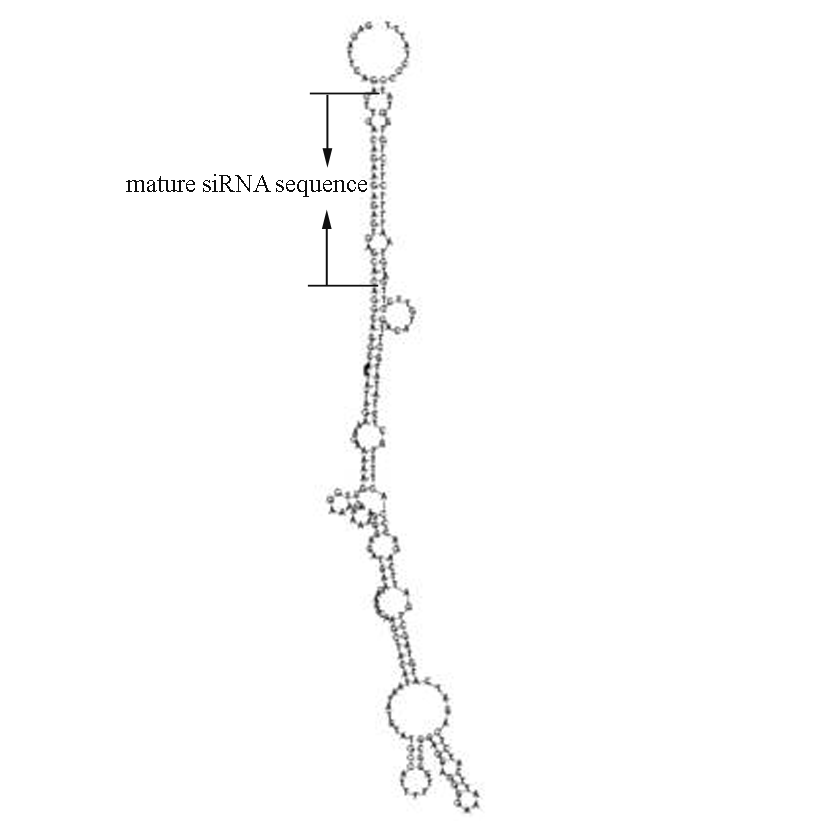

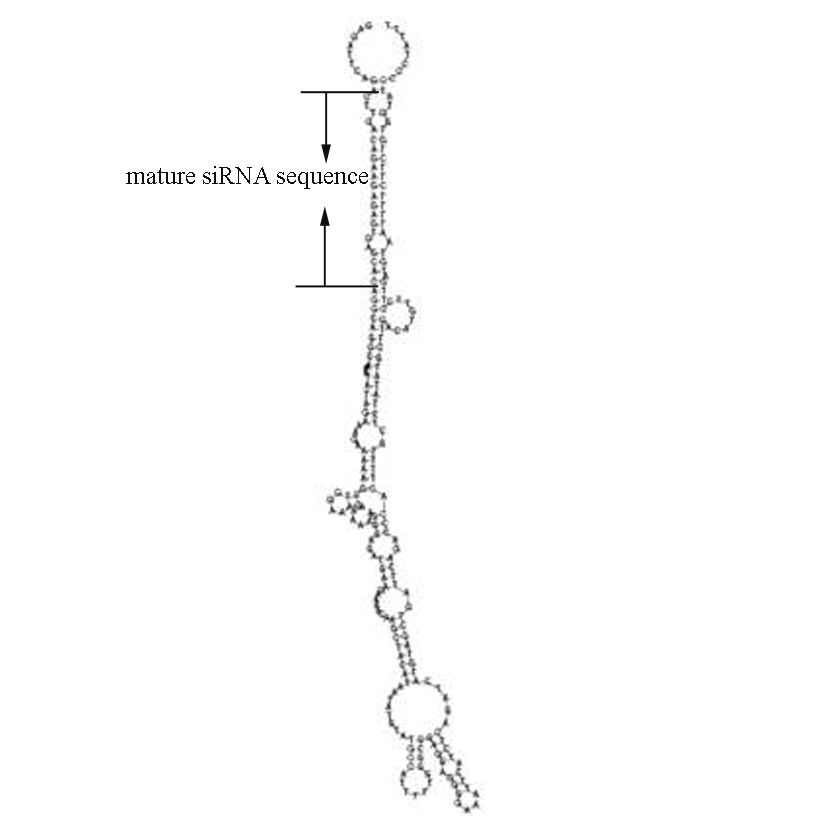


Pau-mR20a Pau-mR20b


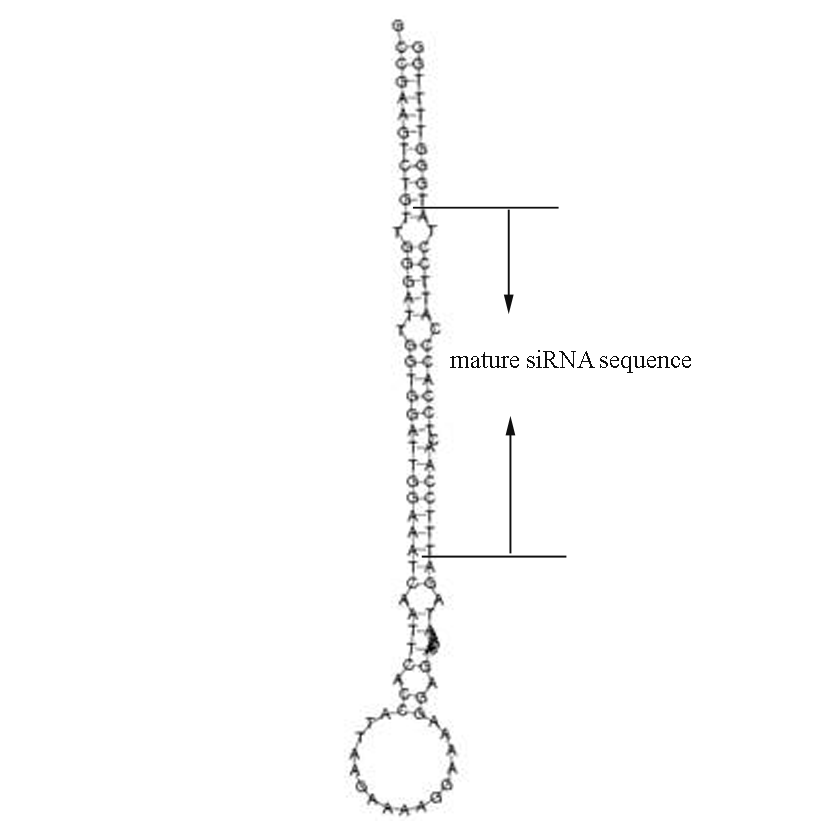


Pau-mR21
